# Characterization of the genomic features and expressed fusion genes in micropapillary carcinomas of the breast

**DOI:** 10.1002/path.4325

**Published:** 2014-02-05

**Authors:** Rachael Natrajan, Paul M Wilkerson, Caterina Marchiò, Salvatore Piscuoglio, Charlotte KY Ng, Patty Wai, Maryou B Lambros, Eleftherios P Samartzis, Konstantin J Dedes, Jessica Frankum, Ilirjana Bajrami, Alicja Kopec, Alan Mackay, Roger A'hern, Kerry Fenwick, Iwanka Kozarewa, Jarle Hakas, Costas Mitsopoulos, David Hardisson, Christopher J Lord, Chandan Kumar-Sinha, Alan Ashworth, Britta Weigelt, Anna Sapino, Arul M Chinnaiyan, Christopher A Maher, Jorge S Reis-Filho

**Affiliations:** 1 The Breakthrough Breast Cancer Research Centre, The Institute of Cancer ResearchLondon, UK; 2 Department of Medical Sciences, University of TurinTurin, Italy; 3 Department of Pathology, Memorial Sloan-Kettering Cancer CenterNew York, NY, USA; 4 Department of Gynecology, University Hospital ZurichZurich, Switzerland; 5 Cancer Research UK Clinical Trials Unit, The Institute of Cancer ResearchSutton, UK; 6 Department of Pathology, Hospital Universitario La Paz, Universidad Autonoma de Madrid, Hospital La Paz Institute for Health Research (IdiPAZ)Madrid, Spain; 7 Michigan Center for Translational Pathology (MCTP), Department of Pathology, University of MichiganAnn Arbor, MI, USA; 8 Washington University Genome Institute, Washington UniversitySt Louis, MO, USA

**Keywords:** breast cancer, micropapillary, RNA sequencing, fusion transcripts, somatic mutation profiling, CDK12, PARP inhibitors

## Abstract

Micropapillary carcinoma (MPC) is a rare histological special type of breast cancer, characterized by an aggressive clinical behaviour and a pattern of copy number aberrations (CNAs) distinct from that of grade- and oestrogen receptor (ER)-matched invasive carcinomas of no special type (IC-NSTs). The aims of this study were to determine whether MPCs are underpinned by a recurrent fusion gene(s) or mutations in 273 genes recurrently mutated in breast cancer. Sixteen MPCs were subjected to microarray-based comparative genomic hybridization (aCGH) analysis and Sequenom OncoCarta mutation analysis. Eight and five MPCs were subjected to targeted capture and RNA sequencing, respectively. aCGH analysis confirmed our previous observations about the repertoire of CNAs of MPCs. Sequencing analysis revealed a spectrum of mutations similar to those of luminal B IC-NSTs, and recurrent mutations affecting mitogen-activated protein kinase family genes and *NBPF10*. RNA-sequencing analysis identified 17 high-confidence fusion genes, eight of which were validated and two of which were in-frame. No recurrent fusions were identified in an independent series of MPCs and IC-NSTs. Forced expression of in-frame fusion genes (*SLC2A1–FAF1* and *BCAS4–AURKA*) resulted in increased viability of breast cancer cells. In addition, genomic disruption of *CDK12* caused by out-of-frame rearrangements was found in one MPC and in 13% of HER2-positive breast cancers, identified through a re-analysis of publicly available massively parallel sequencing data. *In vitro* analyses revealed that *CDK12* gene disruption results in sensitivity to PARP inhibition, and forced expression of wild-type CDK12 in a CDK12-null cell line model resulted in relative resistance to PARP inhibition. Our findings demonstrate that MPCs are neither defined by highly recurrent mutations in the 273 genes tested, nor underpinned by a recurrent fusion gene. Although seemingly private genetic events, some of the fusion transcripts found in MPCs may play a role in maintenance of a malignant phenotype and potentially offer therapeutic opportunities.

## Introduction

Micropapillary carcinomas (MPCs) are a histological special type of breast cancer accounting for up to 7% of all invasive breast carcinomas 1. These tumours display a unique growth pattern featuring clusters of tumour cells displaying inverted polarity immersed in a spongy stroma, and often display extensive vascular invasion 1. Microarray-based comparative genomic hybridization (aCGH) analysis carried out by our group has revealed that pure and mixed MPCs are remarkably similar at the genetic level 2 and harbour a constellation of genetic aberrations that is distinct from that of grade- and oestrogen receptor (ER)-matched invasive carcinomas of no special type (IC-NSTs, also known as invasive ductal carcinomas of no special type) 2,3. Our genomic analysis, however, did not identify any specific genomic aberration that may explain the distinctive morphology and clinical behaviour of MPCs.

Some histological special types of breast cancer have been shown to be underpinned by highly recurrent fusion genes or somatic mutations (reviewed in refs 4 and 5). For instance, adenoid cystic carcinomas and secretory carcinomas of the breast have been shown to be characterized by recurrent specific chromosomal translocations that lead to the formation of the recurrent fusion genes *MYB–NFIB* 6 and *ETV6–NTRK3* 7, respectively, while lobular carcinomas are underpinned by E-cadherin loss of function 4,8,9.

Massively parallel sequencing studies are providing a comprehensive characterization of the repertoire of mutations and fusion genes in different types of cancer 10–16. In breast cancer, RNA-sequencing studies have identified a panoply of expressed fusion genes 12–14,17, although the majority of these appear to exist at very low prevalence or represent private events (ie identified only in the index case) 12,17,18. It is currently believed that most of these fusion genes may be mere passenger events 12,19. Recently, however, RNA sequencing has identified two classes of recurrent gene rearrangements in IC-NSTs, involving genes encoding microtubule-associated serine-threonine kinase (MAST) and members of the Notch family 17. Furthermore, the presence of a chromosomal rearrangement resulting in the formation of a potentially functionally relevant *MAGI3–AKT3* chimeric protein has been described in a subset of breast cancers 15.

Given the previous identification of pathognomonic fusion genes and somatic mutations in histological special types of breast cancer and the recent identification of recurrent expressed fusion genes in breast cancer, the aims of this study were to determine (i) if MPCs harbour recurrent mutations affecting 273 genes, either recurrently mutated in breast cancer or DNA repair-related, or (ii) if MPCs are underpinned by highly-recurrent expressed fusion genes. Furthermore, we sought to determine the biological significance of selected fusion genes identified in MPCs *in vitro*.

## Materials and methods

### Tumour samples

The study design is illustrated in Supplementary Figure 1 and power calculations are described in the Supplementary methods. In brief, two cohorts of MPCs were analysed: 16 formalin-fixed, paraffin-embedded (FFPE) MPCs (Table1), five of which had matched frozen material, and 14 additional FFPE MPCs, employed as a validation cohort. In addition, control groups of 16 IC-NSTs and 14 IC-NSTs grade- and ER-matched to the two series of MPCs were employed. Finally, an additional 48 grade 3 IC-NSTs were retrieved from the authors' institutions and surveyed for the presence of specific fusion transcripts (Supplementary methods and Supplementary Table 1). This study was approved by the authors' local research ethics committees.

**Figure 1:**
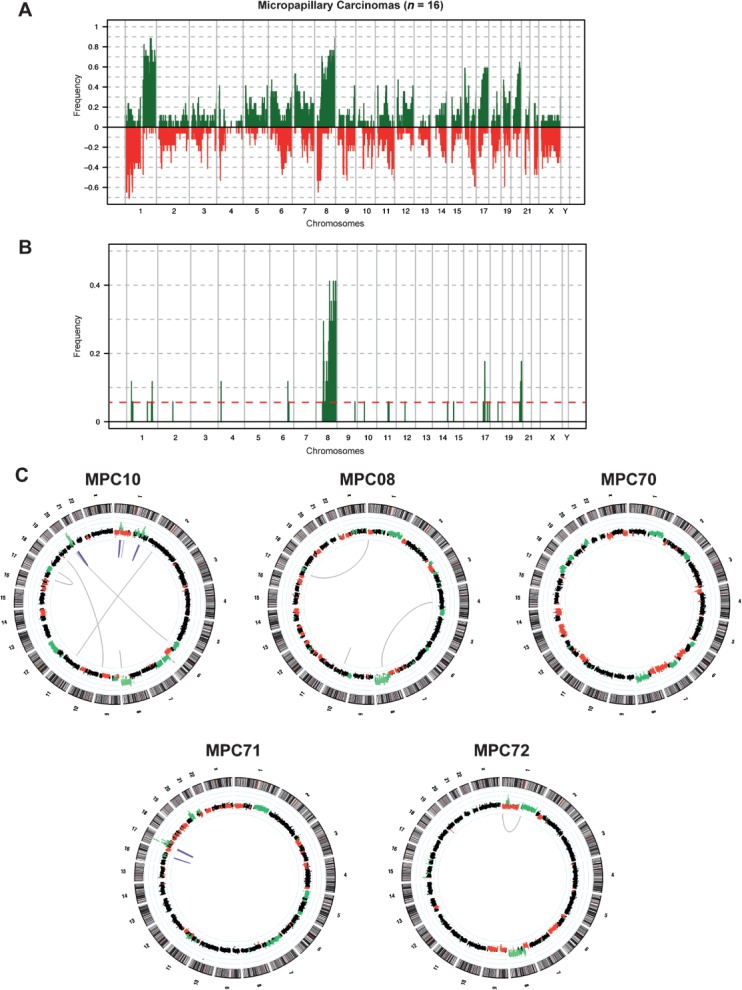
Microarray-based comparative genomic hybridization frequency plots and RNA-sequencing circos plots of micropapillary carcinomas of the breast. (A) Frequency plot of copy number gains and losses in 16 micropapillary carcinomas of the breast. The proportion of tumours in which each bacterial artificial chromosome (BAC) clone is gained (green bars) or lost (red bars) is plotted (*y*-axis) for each BAC clone according to its genomic position (*x*-axis). (B) Frequency plot of amplifications in 16 micropapillary carcinomas of the breast. The proportion of tumours in which each BAC clone is amplified (green bars) is plotted (*y*-axis) for each BAC clone according to its genomic position (*x*-axis). The red dashed line indicates the threshold for recurrent events. (C) High-confidence validated expressed fusion genes are plotted in purple and high-confidence non-validated fusions in grey, linking the genomic locus of each partner of the fusion genes. Genome plots, based on the 32K aCGH data, are plotted in the inner circle (green, copy number gains; red, copy number losses; black, no copy number changes). MPC, micropapillary carcinoma of the breast.

**Table 1:** Main cohort of 16 microdissected MPCs subjected to microarray-based comparative genomic hybridization and Sequenom MassARRAY OncoCarta analysis. Five of the cases were subjected to RNA massively parallel sequencing due to the availability of a frozen specimen

Sample	Pure/mixed	Specimen type	Grade	ER	PR	HER2	RNA-seq	Mutation*
MPC10	Pure	Frozen	3	Positive	Positive	Negative	Y	None
MPC08	Pure	Frozen	2	Positive	Positive	Negative	Y	None
MPC70	Pure	Frozen	2	Positive	Positive	Negative	Y	None
MPC72	Pure	Frozen	3	Positive	Negative	Negative	Y	None
MPC71	Pure	Frozen	3	Positive	Positive	Positive	Y	None
MPC37	Mixed	Frozen	3	Positive	Positive	Negative	NP	None
MPC06	Pure	FFPE	2	Positive	Positive	Negative	NP	None
MPC11	Pure	FFPE	2	Positive	Positive	Negative	NP	None
MPC02	Pure	FFPE	2	Positive	Positive	Negative	NP	None
MPC04	Pure	FFPE	3	Positive	Negative	Negative	NP	None
MPC01	Pure	FFPE	2	Positive	Positive	Negative	NP	None
MPC23	Pure	FFPE	3	Positive	Negative	Negative	NP	None
MPC25	Mixed	FFPE	2	Positive	Positive	Negative	NP	*PIK3CA* H1047R
MPC45	Mixed	FFPE	3	Positive	Positive	Positive	NP	None
MPC38	Mixed	FFPE	3	Positive	Positive	Negative	NP	None
MPC53	Mixed	FFPE	3	Negative	Negative	Positive	NP	None

ER, oestrogen receptor; FFPE, formalin-fixed, paraffin-embedded; NP, not performed; PR, progesterone receptor; RNA-seq, paired-end massively parallel mRNA sequencing; Y, yes.

* Mutations in the hotspot regions of 19 genes assessed by the Sequenom OncoCarta v1.0.

### Immunohistochemistry

Representative sections of each case were subjected to immunohistochemical assessment using antibodies against epithelial membrane antigen, ER, progesterone receptor (PR), and HER2 as previously described 2,3 and were reviewed by at least two pathologists (CM, AS, and/or JSR-F), using previously defined scoring systems and cut-offs 2,3,20 (Supplementary methods and Supplementary Table 2).

### Microdissection, DNA extraction, and RNA extraction

Representative 8-µm-thick sections of the MPCs and IC-NSTs were subjected to microdissection with a sterile needle under a stereomicroscope (Olympus SZ61, Tokyo, Japan) to ensure a percentage of tumour cells greater than 90%, as previously described 3,21. DNA extraction, quantification, and quality control assessment were performed as previously described 3,21 (Supplementary methods). From eight of the 16 MPCs microdissected, adjacent normal breast tissue was successfully microdissected.

### Microarray comparative genomic hybridization (aCGH)

Sixteen MPCs and 16 grade-, ER-, and HER2-matched IC-NSTs were subjected to aCGH analysis using a platform that comprised ∼32 000 BAC clones tiled across the genome 22. This platform has been shown to be as robust as, and to have comparable resolution to high-density oligonucleotide arrays 23–25. DNA labelling, array hybridization, image acquisition, and data analysis were performed as previously described 3,22,26,27 (Supplementary methods). Data, the analysis history, script, and code are available at http://rock.icr.ac.uk/collaborations/Mackay/Micropapillary.

### Mutation screening and validation

Sixteen MPCs and 16 grade-, ER-, and HER2-matched IC-NSTs were subjected to hotspot mutation screening of 19 known cancer genes using the OncoCarta Panel v 1.0 (Sequenom, San Diego, CA, USA) and validated using Sanger sequencing as previously described 22,27. For eight of these cases, sufficient DNA from tumour and normal breast was available for targeted capture massively parallel sequencing analysis using a bait library targeting 273 genes either recurrently mutated in breast cancer or DNA repair-related genes as previously described 2,28–35 (Supplementary methods and Supplementary Table 3).

### Paired-end massively parallel RNA sequencing

Five pure frozen MPCs were subjected to mRNA massively parallel sequencing, which was performed according to the standard Illumina mRNA paired-end library protocol (Illumina Inc, San Diego, CA, USA) as previously described 36. Paired-end sequencing was performed using 2 × 54 bp cycles on the Genome Analyser IIx (Illumina; Supplementary methods). Data were aligned to the genome and transcriptome using Bowtie 37. Mate-pairs supporting novel chimeric transcripts were identified using ChimeraScan version 4.0.3.0 as previously described 17,37. High-confidence chimeric transcripts were nominated subsequent to further filtering to remove multi-mapping reads and to exclude false-positive nominations 37.

### Reverse-transcription PCR (RT-PCR), PCR, and Sanger sequencing validation

Nominated fusion genes were validated in five index cases by RT-PCR and Sanger sequencing as previously described 18 (Supplementary methods). Validated in-frame and out-of-frame fusion genes with recurrent partners found in independent datasets 12,15–17 were screened at the cDNA level in an independent cohort of MPCs (*n =* 14), grade- and ER-matched IC-NSTs (*n =* 14), and grade 3 IC-NSTs (*n =* 48) using RT-PCR or quantitative real-time PCR (qRT-PCR; Supplementary methods).

### Constructs for functional analyses

The *SLC2A1–FAF1* and *BCAS4–AURKA* fusion open reading frames (ORFs) were PCR-amplified from the index tumour (MPC10) and cloned into a mammalian expression vector pCMVentry, with a C-terminal DDK tag (OriGene, Rockville, MD, USA). Full-length expression constructs of *FAF1*, *AURKA*, and *RAE1* with DDK tags were obtained from OriGene. Expression of the fusion and wild-type protein constructs was detected with the anti-DDK monoclonal antibody 4C5 (Origene) by western blotting of 20 µg of whole cell protein lysate as previously described 38. Sequences of the ORFs are available at http://rock.icr.ac.uk/collaborations/Mackay/Micropapillary.

### Cell line models

The ER-positive breast cancer cell lines, MCF7, BT474, T47D, and ZR75.1, whose phenotypic characteristics and patterns of copy number aberrations are consistent with those of the MPCs (Supplementary Table 4), and 12 *HER2*-amplified cell lines (JIMT1, UACC812, UACC893, VP229, SKBR3, ZR75.30, HCC1569, HCC1954, MDA-MB-453, MDA-MB-361, SUM225, and SUM190) were included in this study. The sources, growth conditions, and authentication methods are described in the Supplementary methods.

### Functional assessment of in-frame fusion genes

Cloned *SLC2A1-FAF1* and *BCAS4-AURKA* fusions, full-length 3′ partner gene constructs or empty vectors were transfected into four ER-positive breast cancer cell lines (MCF7, BT474, T47D, and ZR75.1) using Lipofectamine 2000 (Invitrogen, Carlsbad, CA, USA). Antibiotic selection was performed as previously described 38, and cell populations were assessed every 24 h, for 9 days, using the CellTiter-Glo® cell viability assay (Promega, Madison, WI, USA) 38, with each reading normalized to day 1 to determine the fold change (Supplementary methods).

### Functional assessment of out-of-frame fusion genes

Short-interfering RNA (siRNA) silencing of *CDK12* was performed as previously described 38,39. Briefly, MCF7, T47D, and SUM149 breast cancer cells were transfected with siGENOME SMARTpools (each containing four distinct siRNAs targeting each gene), a non-targeting negative control siRNA (siCON), and a positive control siRNA targeting *PLK1* (siCDK12: M-004031-03; siControl: D-001206-14; siPLK1: M-003290-01; Fermentas, Germany). Transfection mixes contained 50 nm siRNA in a final volume of 100 µl, together with Lipofectamine RNAiMAX reagent (Invitrogen). CDK12 silencing was confirmed at the protein level by western blotting using an anti-CDK12 antibody (ab37914; Abcam, Cambridge, MA, USA).

### Long-term survival assay

Long-term 14-day survival assays were performed using six-well plates in triplicate as previously described 38,39. The sulforhodamine B (SRB) (Sigma) assay was employed as readout as described previously 38,39 (Supplementary methods). The PARP inhibitor BMN673 was a kind gift from BioMarin Pharmaceuticals (San Rafael, CA, USA), and olaparib (AZD2281/KU0058948) was obtained from SelleckBio (Munich, Germany).

### Analysis of RAD51 foci formation

Nuclear RAD51 and phospho-γ-H2AX foci were visualized and quantified as previously described 38,39 and used as surrogate markers for induction of DNA double-strand breaks (DSBs) and competent homologous recombination (HR) DNA repair, respectively (Supplementary methods).

### Statistical analyses

The statistical analysis methods employed are described in the Supplementary methods.

## Results

### The landscape of gene copy number aberrations and somatic mutations in MPCs

Genome-wide aCGH profiling of 16 MPCs revealed extensive changes in the genome, ranging from 10.4% to 54.3% of BACs showing either gains or losses (mean: 28.5%; median: 28.9%). Consistent with the results of our previous studies 2,3,40, the most frequent recurrent changes included gains of 1q, 8q, 17q, and 20q, and losses of 1p 8p, 13q, 16q, and 22q (Figure 1 and Supplementary Table 5). The majority of MPCs (10/16 cases; 62.5%) showed high-level gain of the whole arm of chromosome 8q, regardless of histological grade, as previously reported 3. Recurrent (*n* > 1) focal (< 2 Mb) amplifications containing known genes were identified on chromosomes 1p34.3–p34.2, 8p12–p11.21, 17q11.1–q21.2, and 20q13.13–q13.2 (Supplementary Table 5).

To determine whether MPCs would be underpinned by a pathognomonic somatic mutation, eight samples were subjected to targeted massively parallel sequencing comprising 273 genes, and 16 samples were subjected to Sequenom mutation profiling using the OncoCarta panel 22,27. We identified recurrent mutations affecting genes of the mitogen-activated protein kinase family (*MAP3K1* in three cases, and *MAP2K6* and *MAP3K4* in one case each) and recurrent splice-site mutations of *NBPF10* (*n =* 2) (Table2). A significant enrichment in genes mutated in luminal B breast cancers was observed in the constellation of mutations found in MPCs; out of the 119 genes most frequently mutated (ie ≥ 4 cases – 3.5%) in luminal B IC-NSTs from The Cancer Genome Atlas (TCGA) study 16, *TP53* (W53^*^), *PIK3CA* (H1047R), *CSMD2* (R3608S), *MAP3K1* (insertions and deletions in three cases), *ATRX* (P667T), *HMCN1* (A537G), *MLL2* (A946E), *SPEN* (ESS2280A), and *ZFHX4* (A2896S) were mutated in MPC samples (representation factor = 2.3; hypergeometric test *p* value < 0.01; Table2). Sequenom analysis confirmed the results of the massively parallel sequencing analysis; of the hotspot mutations included in the OncoCarta panel, the *PIK3CA* H1047R mutation was identified in the MPC reported to have this mutation by massively parallel sequencing (MPC25). This mutation was also validated by Sanger sequencing (Supplementary Figure 2). In addition, *MAP3K1*, *MAP3K4*, *PIK3CA*, *NBPF10*, *CECR2*, and *CSMD2*, which were mutated in the MPCs analysed here, were also shown to be mutated in at least one of the four cases of pure and mixed MPC included in the TCGA study (http://www.cbioportal.org/public-portal/study.do?cancer_study_id=brca_tcga_pub, assessed on 3 December 2013). Taken together, our results suggest that MPCs are not driven by a pathognomonic mutation affecting any of the 273 genes included in our targeted capture massively parallel sequencing panel, and that genes mutated in luminal B IC-NSTs are also mutated in MPCs.

**Table 2:** Somatic mutations identified by massively parallel sequencing in eight micropapillary carcinomas of the breast

Gene	Mutation	Mutation type	MAF	Depth	Tumour sample
*AGFG2*	A443T	Non-synonymous coding	35.70%	366	MPC02T
*AKT1*	E17K	Non-synonymous coding	31.70%	253	MPC06T
*APC*	R2714L	Non-synonymous coding	4.80%	156	MPC25T
*ATRX*	P667T	Non-synonymous coding	9.43%	156	MPC37T
*CDC25B*	R137W	Non-synonymous coding	18.49%	799	MPC53T
*CHD4*	N789I	Non-synonymous coding	6.90%	284	MPC06T
*CSMD2*	R3608S	Non-synonymous coding	5.81%	149	MPC25T
*CUBN*	N3576K	Non-synonymous coding	7.46%	204	MPC37T
*DCHS2*	R434S	Non-synonymous coding	10.42%	147	MPC37T
*DOCK11*	H833D	Non-synonymous coding	30.80%	132	MPC08T
*DST*	P5601Q	Non-synonymous coding	7.81%	130	MPC10T
*FBN1*	P83S	Non-synonymous coding	16.00%	273	MPC53T
*FOXA1*	Y259D	Non-synonymous coding	23.80%	179	MPC06T
*FOXA1*	P248S	Non-synonymous coding	10.30%	246	MPC06T
*GPR98*	L3724F	Non-synonymous coding	23.74%	330	MPC53T
*HMCN1*	A537G	Non-synonymous coding	13.60%	76	MPC10T
*MAP1A*	S604^*^	Stop gained	38.70%	47	MPC10T
*MAP2K6*	S35P	Non-synonymous coding	18.40%	273	MPC53T
*MAP3K4*	G743R	Non-synonymous coding	29.90%	459	MPC53T
*MLL2*	A946E	Non-synonymous coding	6.10%	207	MPC08T
*MST1L*	W378G	Non-synonymous coding	23.28%	354	MPC25T
*PIK3CA*	H1047R	Non-synonymous coding	64.80%	111	MPC25T
*RBBP8*	R805L	Non-synonymous coding	4.40%	216	MPC08T
*NBPF10*	–	Splice site donor	37.50%	43	MPC02T
*NBPF10*	–	Splice site donor	28.10%	50	MPC25T
*SHROOM4*	P769S	Non-synonymous coding	21.05%	398	MPC53T
*SRCAP*	V2835M	Non-synonymous coding	20.59%	137	MPC37T
*TP53*	W53^*^	Stop gained	55.30%	647	MPC53T
*ZFHX4*	A2896S	Non-synonymous coding	6.20%	237	MPC08T
*MAP3K1*	R271fs	Indel – frameshift – exon	31.58%	312	MPC02T
*MAP3K1*	K1037fs	Indel – frameshift	40.00%	168	MPC02T
	L1491LQP	Indel – codon insertion	32.14%	263	MPC06T
*ATN1*	Q488QQ	Indel – codon insertion	26.67%	88	MPC10T
*SPEN*	ESS2280A	Indel – codon change and deletion	34.62%	91	MPC11T
*MAP3K1*	T918fs	Indel – frameshift	26.21%	183	MPC25T
*MAP3K1*	F884fs	Indel – frameshift	37.50%	147	MPC25T

MAF, mutant allelic frequency; –, splice site.

### Identification of expressed fusion transcripts in MPCs

**Figure 2:**
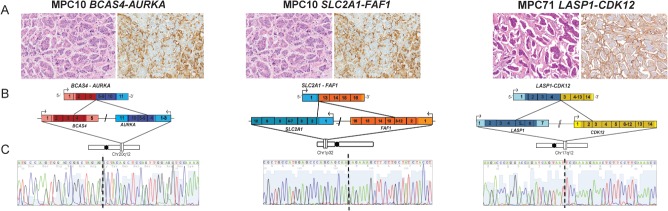
Structure of validated high-confidence fusion genes in micropapillary carcinomas. Sanger sequencing validation of functionally assessed fusion genes in the index cases of micropapillary carcinomas (MPCs) (*BCAS4–AURKA* and *SLC2A1–FAF1* in MPC10 and *LASP1-CDK12* in MPC71). (A) Haematoxylin and eosin (H&E) and epithelial membrane antigen (EMA) staining of representative areas of the MPCs harbouring the *BCAS4–AURKA*, *SLC2A1–FAF1*, and *LASP1–CDK12* fusion genes (10× original magnification). (B) Schematic representation of nominated fusion transcripts. Fusion junctions with respective exon numbers are shown, while paler colours indicate 3′ and 5′ UTRs. (C) cDNA level sequence chromatograms spanning the junction (dotted line) of the fusion transcript.

RNA sequencing of five frozen MPCs resulted in a total amount of sequencing per sample of 1.33–1.92 Gb per lane, median 1.37 Gb per lane (Supplementary Table 6). Fusion transcript detection using ChimeraScan 37 identified 18 high-confidence chimeric transcripts in four MPCs (MPC10, MPC08, MPC71, and MPC72; Figure 1 and Table3). In MPCs harbouring expressed fusion genes, the number of nominated high-confidence chimeras ranged from 11 in MPC10 to one in MPC72. MPC70 had no nominated high-confidence chimeric transcripts; no differences in the total number of aligned reads were found between cases that expressed fusion genes and those that did not (data not shown). Nominated chimeric transcripts were subsequently validated in each index case by RT-PCR and Sanger sequencing (Figure 2), of which eight of the 17 fusions were validated (Table3). qRT-PCR was used to confirm expression of in-frame fusion genes (Supplementary Figure 3). One or both of the partner genes involved in seven of the eight fusion genes were amplified as defined by aCGH analysis (Figure 1 and Table3). Of the eight validated fusions, two were predicted to be in-frame (*SLC2A1–FAF1* and *BCAS4–AURKA*) and were both present in a single tumour (ie MPC10; Figure 2 and Supplementary Figure 3). Both of these fusions were found to map to breakpoints of amplification (Table3).

**Table 3:** Identification of expressed chimeric transcripts in five micropapillary breast carcinomas

Sample ID	Gene 5′	Gene 3′	Mapping 5′	Mapping 3′	Total reads	Spanning reads	In frame	Validated	Copy number 5′	Copy number 3′
MPC10	*ELMO2*	*RAE1*	chr20:45014762	chr20:55943760	25	14	No	Y	Gain	Amp
MPC10	*BCAS4*	*AURKA*	chr20:49411466	chr20:54944444	24	1	Yes	Y	Gain	Amp
MPC10	*SLC2A1*	*FAF1*	chr1:43424304	chr1:50906934	12	5	Yes	Y	Amp	Gain
MPC10	*TSEN54*	*UNC84A*	chr17:73512608	chr7:934970	7	0	No	N	Gain	Gain
MPC10	*CD46*	*USH2A*	chr1:207925401	chr1:215796235	6	1	No	Y	Amp	Gain
MPC71	*LASP1*	*CDK12*	chr17:37026111	chr17:37646809	5	4	No	Y	Amp	Amp
MPC10	*DENND1C*	*SURF1*	chr19:6470305	chr9:136218665	4	0	Yes	N	No change	No change
MPC10	*UBE2V1*	*SULF2*	chr20:48713208	chr20:46286150	4	1	No	Y	Amp	Amp
MPC08	*BC018860*	*CDK5RAP3*	chr1:566461	chr17:46052524	3	1	No	N	No change	No change
MPC10	*LRRC6*	*HPRG1*	chr8:133584447	chr8:133572744	3	0	No	N	Amp	Amp
MPC08	*PDLIM5*	*TCEB1*	chr4:95373037	chr8:74858633	3	3	No	N	No change	Amp
MPC08	*TSPAN14*	*DYDC2*	chr10:82214037	chr10:82126443	3	1	No	N	Amp	No change
MPC10	*IFI44L*	*IFI44*	chr1:79086087	chr1:79115476	2	0	No	N	Loss	Loss
MPC10	*MRPL21*	*BX640963*	chr11:68660870	chr2:69686413	2	0	No	N	No change	No change
MPC10	*SYNGAP1*	*RALY*	chr6:33387846	chr20:32664833	2	0	No	N	Gain	Gain
MPC72	*MUC1*	*C1orf86*	chr1:155161501	chr1:2115916	2	0	No	N	Gain	Loss
MPC71	*CYB5B*	*CALB2*	chr16:69458497	chr16:71416621	2	2	No	Y	No change	No change
MPC71	*NSF*	*C17orf57*	chr17:44668037	chr17:45438743	2	2	No	Y	Amp	No change

5′, 5′ partner gene; 3′, 3′ partner gene; total reads, total number of reads supporting the nominated fusion; spanning reads, number of reads spanning the fusion junction.

Copy number derived from smoothed cbs ratios of aCGH data; loss, copy number loss; no change, no copy number change; gain, copy number gain, amp, amplification.

RT-PCR analysis of a series of 14 independent MPCs and 62 IC-NSTs (Supplementary Table 1 and Supplementary Figure 4) revealed no additional case harbouring these fusion transcripts. Of interest, however, both *SLC2A1–FAF1* and *BCAS4–AURKA* fusion genes were found to be present in the lymph-node metastasis of the index case MPC10 (Supplementary Figure 3). We next sought to determine whether one or both partner genes of all validated chimeric transcripts would be involved in fusion genes in other breast cancers. Publicly available next-generation sequencing breast cancer datasets (*n =* 185) 12,15–17,41–43 were interrogated and revealed that *BCAS4* is the 5′ partner gene in the validated fusion *BCAS4–BCAS3* in MCF7 cells 13,17 and that *FAF1* is involved in a DNA rearrangement in an ER-negative/HER2-negative primary breast cancer. None of the other genes of these in-frame fusions were found to be involved in previously reported fusion genes in breast cancers. Of the out-of-frame validated chimeras, we identified recurrent expressed chimeric transcripts involving *CDK12* (2.7%) and *RAE1* (1%), and DNA rearrangements involving *C17ORF57* (0.5%), *NSF* (0.5%), *USH2A* (0.5%), and *LASP1* (1%) from unselected breast cancers in external datasets 12,15–17,41–43 (Supplementary Table 7).

### Assessment of the biological relevance of selected fusion genes identified in MPCs

**Figure 3:**
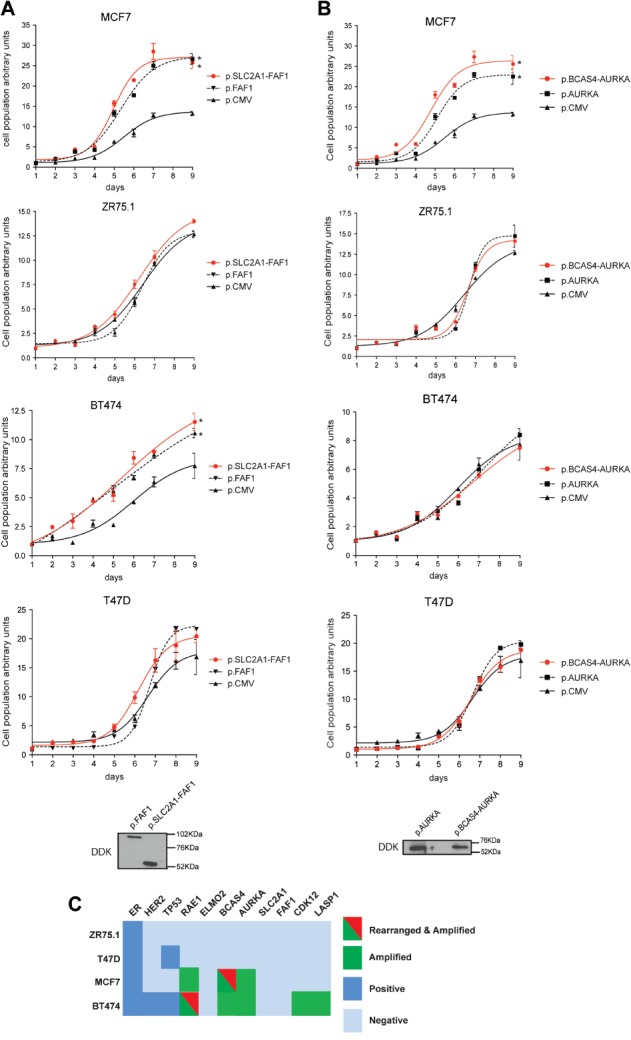
Functional assessment of in-frame expressed fusion genes. To determine the biological significance of the in-frame fusion genes identified in MPCs, forced expression of each fusion gene (*SLC2A1–FAF1*, A and *BCAS4–AURKA*, B), the 3′ partner gene, and an empty vector was performed in a panel of breast cancer cell lines. The fold change in cell population (*y*-axis) is plotted against growth time (days, *x*-axis). Red, solid black, and dashed black lines denote growth curves following transfection with fusion gene constructs (ie *SLC2A1–FAF1* or *BCAS4–AURKA*), full-length 3′ partner genes (ie *FAF1* or *AURKA*), and empty vector (pCMV), respectively. Western blotting using anti-DDK antibody was employed to confirm exogenous expression of cDNA constructs following transfection in MCF7 cells (A and B). In A and B, statistically significant differences are highlighted with an asterisk. (C) A matrix illustrating the pathological phenotype and aberrations affecting endogenous fusion gene partners in the cell lines employed, where dark blue squares represent positivity in a marker, light blue squares represent negativity in a marker, green squares denote amplification of a gene, and red denotes rearrangement of a gene.

Given that *SLC2A1–FAF1* and *BCAS4–AURKA* fusion genes were present in both the primary tumour and lymph node metastasis of MPC10, we hypothesized that, albeit restricted to a single case, these fusion genes could constitute driver events. Therefore, the chimeric transcript and each partner gene were transiently expressed in four ER-positive/HER2-negative breast cancer cell lines, including MCF7, which is reported to recapitulate many of the phenotypic characteristics of MPCs 44. Forced expression of *SLC2A1–FAF1* caused a significant increase in cell proliferation compared with the empty vector control (as measured by fold change in cell viability on day 9 in each cell line; Figure 3A) in two out of four cancer cell lines (ie MCF7 and BT474, *p* < 0.05, one-way ANOVA). Forced expression of full-length *FAF1* had a similar effect only in MCF7 and BT474 cells (Figure 3A). *BCAS4–AURKA* forced expression caused a significant increase in cell proliferation, compared with the empty vector, in one cell line (MCF7, *p* < 0.05, one-way ANOVA). Again, forced expression of the 3′ partner, *AURKA*, recapitulated these results only in MCF7 (Figure 3B). These data suggest that some private, or low-frequency, fusion genes may provide a selective advantage, which may also be dependent on the phenotype and genetic make-up of the cell harbouring them (Figure 3C).

**Figure 4:**
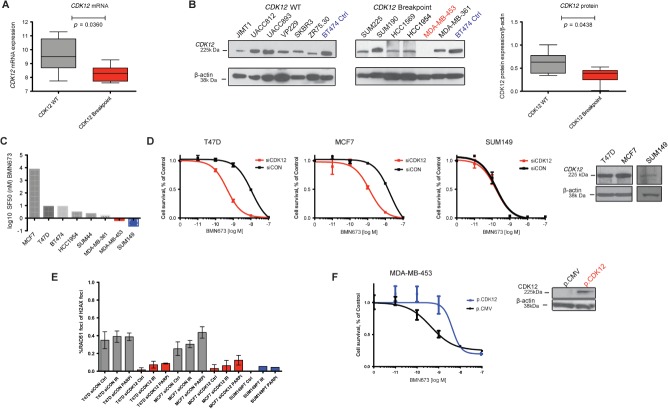
Disruption of *CDK12* leads to PARP inhibitor sensitivity. (A) mRNA expression of CDK12 in *HER2*-amplified cell lines with a breakpoint spanning the *CDK12* locus shows significantly lower levels of *CDK12* mRNA compared with cells without a breakpoint spanning *CDK12*. (B) Western blot analysis of endogenous CDK12 in a panel of *HER2*-amplified cell lines relative to β-actin loading control. BT474 (blue) was employed as a loading control for normalization of the results of the western blot. Note that MDA-MB-453 shows no CDK12 expression (red), and relative quantification of CDK12 protein expression relative to β-actin, showing a significantly lower level of expression in cell lines with a breakpoint spanning *CDK12* than those without (*p* = 0.0438, *t*-test). (C) Log_10_ SF50 of *HER2*-amplified cell lines to the PARP inhibitor BMN673. SUM149 in blue as a positive control, given that this cell line harbours a *BRCA1* mutation. (D) CDK12 silencing results in increased sensitivity to BMN673 in T47D and MCF7 ER-positive breast cancer cells. The *BRCA1* mutant SUM149 cells displayed no increase in sensitivity upon CDK12 silencing, suggesting that the PARP inhibitor sensitivity caused by silencing of this gene results from impaired homologous recombination DNA repair. Western blot showing expression of CDK12 in T47D, MCF7, and SUM149 cells relative to β-actin loading control. (E) Percentage of cells with RAD51 positive foci normalized to phospho-γ-H2AX foci formation without treatment (Ctrl), 6 h after 10 Gy (IR) of irradiation, and with 1 mm BMN673 for 24 h (PARPi), with (red) and without (grey) CDK12 silencing. (F) Reconstitution of full-length CDK12 (blue) and empty vector (p.CMV) (black) in MDA-MB-453 cells treated with BMN673. Western blot showing expression of CDK12 in MDA-MB-453 cells transfected with full-length CDK12.

Out-of-frame fusion genes may provide a selective advantage to cancer cells harbouring them by disrupting the expression of genes with tumour suppressor roles 45,46. Given that *CDK12* maps to the smallest region of amplification of the *HER2* amplicon, and recurrent fusions were identified in 13% (6/47) *HER2*-amplified tumours (Supplementary Table 7), we hypothesized that disruptions involving *CDK12* were due to a copy number breakpoint within the amplicon. By mining aCGH and matched gene expression data from *HER2*-amplified cells lines 47, we identified 7/14 *HER2*-amplified cells where a breakpoint within the *HER2* amplicon mapped to *CDK12* (Supplementary Table 4). A significant reduction in *CDK12* mRNA expression was observed in these cell lines relative to *HER2*-amplified breast cancer cell lines that did not harbour a breakpoint involving *CDK12* (Figure 4A; *p* = 0.0360, *t*-test). Assessment of CDK12 protein expression in this panel of cells revealed a significantly lower level of protein expression (*p* = 0.0438, *t*-test; Figure 4B), with MDA-MB-453 showing no detectable levels of CDK12 protein expression. CDK12 has recently been reported to be involved in the maintenance of genomic stability through regulation of genes involved in the DNA damage response pathway including *BRCA1*, *ATR*, *FANCI*, and *FANCD2*, and cells devoid of CDK12 have been reported to be sensitive to DNA-damaging agents 48. To determine whether *HER2*-amplified cells with breakpoints in *CDK12* were sensitive to PARP inhibitors, we investigated the sensitivity of *HER2*-amplified and ER-positive breast cancer cell lines to BMN673 (Figure 4C) and olaparib (Supplementary Figure 5). Although there was no difference in sensitivity comparing the SF50 to cell lines with and without CDK12 disruption (*p* = 0.207, BMN673 and *p* = 0.375, olaparib, *t*-test), MDA-MB-453 cells that are null for CDK12 expression were sensitive to both BMN673 and olaparib. Silencing of CDK12 mediated by siRNA in ER-positive cell lines MCF7 and T47D resulted in increased sensitivity to the PARP inhibitor BMN673 (Figure 4D). Furthermore, the ability to elicit RAD51 foci formation upon treatment with ionizing radiation or the PARP inhibitor BMN673 was diminished upon CDK12 silencing (Figure 4E and Supplementary Figure 6). Reconstitution of wild-type CDK12 in MDA-MB-453 CDK12 null cells rendered cells more resistant to both BMN673 (Figure 4F) and olaparib (Supplementary Figure 5).

Taken together, our results demonstrate that MPCs do not harbour highly-recurrent fusion genes; however, some of the in-frame private fusion genes identified in this study have a biological impact that is likely to be context-dependent and may be part of a convergent phenotype. Loss of CDK12 due to breakpoints in the *HER2* amplicon results in sensitivity to PARP inhibitors, suggesting that some out-of-frame fusion genes may represent bona fide loss-of-function genomic events, and potentially targetable somatic genetic aberrations.

## Discussion

In this analysis of breast MPCs, not only have we confirmed the patterns of gene copy number aberrations previously reported by our group 2,3 in this special type of breast cancer, but we have also provided direct evidence to rule out two potential mechanisms that result in breast cancers displaying a micropapillary phenotype: (i) pathognomonic mutations in genes recurrently mutated in breast cancer and DNA repair-related genes and (ii) highly-recurrent expressed fusion genes. In fact, our analysis revealed recurrent mutations affecting MAPK genes. In addition, mutations affecting genes often mutated in luminal B IC-NSTs have also been found in individual MPC samples (Table2). This finding confirms our previous observation that MPCs have a constellation of genetic aberrations similar to those of luminal B breast cancers 2,3 and rules out the possibility that the MPC phenotype is driven by pathognomonic mutations affecting any of the 273 recurrently mutated breast cancer and DNA repair-related genes included in the targeted capture sequencing platform employed (Supplementary Table 3) and in the Sequenom OncoCarta panel. Furthermore, paired-end massively parallel RNA-sequencing revealed that although fusion genes are present in a proportion of MPCs, these constitute private genetic events (ie restricted to the index case) and often map to regions of amplification.

Two of the in-frame expressed fusion genes identified in this study, *SLC2A1–FAF1* and *BCAS4–AURKA*, were present both in the primary tumour MPC10 and in the corresponding synchronous lymph-node metastasis (MPC10LND). Although this observation may merely indicate that these fusion genes were present in the modal clonal population of MPCs, it is possible that their maintenance in the metastatic deposit was due to a selective advantage conferred by the expression of the chimeric fusion genes. To test whether these fusion genes would confer a growth/survival advantage, we forced the expression of the chimeric fusion transcripts and each 3′ partner in four ER-positive, HER2-negative breast cancer cell lines. Similar to driver fusion genes identified by RNA sequencing analysis of breast cancer 17, ectopic overexpression of these in-frame fusion genes resulted in increased proliferation of multiple breast cancer cell lines, which was similar to the effect of the ectopic expression of the 3′ partner genes. It should be noted, however, that different ER-positive breast cancer cell lines responded differently to the forced expression of the fusion genes *SLC2A1–FAF1* and *BCAS4–AURKA*, and their wild-type fusion gene partners (Figure 3). The *SLC2A1–FAF1* fusion involves a promoter swap of the major glucose transporter *SLC2A1* exon 1 fused to exons 13–16 of *FAF1. FAF1* (FAS associate factor 1) encodes for a protein that binds to FAS antigen and initiates apoptosis; its down-regulation may contribute to tumourigenesis, through the regulation of apoptosis and NFκB activity, as well as in ubiquitination and proteasomal degradation 49. The fusion gene found in MPC10 contains the ubiquitin association and ubiquitin-like regulatory X domain 49. The *BCAS4–AURKA* fusion gene results in the preservation of the AURKA kinase domain being driven by the promoter sequences of *BCAS4*. The loci of *BCAS4* and *AURKA* (ie 20q13) are amplified in MPC10 and in MCF7 cells. *AURKA* encodes for Aurora Kinase A, a serine-threonine kinase mainly involved in centrosome duplication, mitotic entry, and spindle assembly 50. Although *AURKA* gene amplification is a common genetic aberration in breast cancer 16, its role as a therapeutic target for breast cancers harbouring amplification of this locus remains to be fully established. Taken together, these findings suggest that some private fusion genes may provide a growth/survival advantage to cancer cells and that this advantage is context-dependent and may be the product of epistatic interactions.

Some of the partner genes of the validated chimeric transcripts found in the present study were shown to be involved in other somatic rearrangements in breast cancers. *CDK12*, *LASP1*, *RAE1*, *C17orf57*, *NSF*, and *USH2A* were partners of out-of-frame, but not in-frame, fusion genes here and in other massively parallel sequencing analyses of breast cancers 12,15–17,41–43 (Supplementary Table 7). It is plausible that these out-of-frame rearrangements result in loss of function of one or both partners involved. Re-analysis of publicly available data revealed that *CDK12* may constitute a tumour suppresser gene as it is recurrently targeted not only by DNA rearrangements in breast (2.6% of unselected breast cancers; 13% of *HER2*-amplified breast cancers) and 1/17 (5%) *HER2*-amplified gastric cancers 51, but also by nonsense mutations in 1.5% of triple-negative breast cancers 43. Consistent with this notion, CDK12 has been shown to be related to DNA repair, given that it plays a role in the regulation of transcription. In addition, depletion of CDK12 results in decreased expression of predominantly long genes with high numbers of exons, in particular DNA damage response genes, including *BRCA1*, *ATR*, *FANCI*, and *FANCD2*. CDK12-deficient cells display spontaneous DNA damage and are sensitive to a variety of DNA-damaging agents 48. Here we have demonstrated that RNA-interference-mediated silencing of CDK12 in ER-positive cell lines resulted in increased sensitivity to PARP inhibition and a reduction in the ability to form RAD51 foci in the nucleus upon DNA damage through irradiation or PARP inhibition. Furthermore, MDA-MB-453 cells, which lack CDK12, displayed sensitivity to PARP inhibition, which was rescued upon re-expression of full-length CDK12. These observations suggest that the subset of *HER2*-amplified breast cancers harbouring a disruption of *CDK12* through somatic rearrangements may benefit from treatment with PARP inhibitors, and provide a molecular rationale for the testing of these agents in HER2-positive disease.

This study has several limitations. First, the small number of cases subjected to mRNA sequencing could have resulted in a type II/β-error in the search of a highly-recurrent pathognomonic event; however, by the sequencing of five samples we should have been able to identify a recurrent event (ie with a prevalence similar to *FOXL2* mutations in granulosa cell tumours of the ovary 52) with 97% statistical power. Second, although cell lines constitutively harbouring the in-frame fusion genes identified in primary MPCs were not available, our results demonstrate that forced expression of *SLC2A1–FAF1* and *BCAS4–AURKA* results in increased growth/survival in multiple breast cancer cells.

In conclusion, our findings demonstrate that the MPC phenotype in breast cancers is neither driven by pathognomonic mutations affecting the 273 recurrently mutated breast cancer and DNA repair-related genes tested in this study, nor is it underpinned by a highly-recurrent pathognomonic expressed fusion gene. Although the fusion genes identified in this study were private events, we have provided circumstantial evidence to suggest that at least some private in-frame expressed fusion genes may also be driver events and impact on cancer cell proliferation. Finally, some of the out-of-frame *CDK12* rearrangements in HER2-positive breast cancers were shown to lead to a potential loss of function and provide a rationale for treating a subset of *HER2*-amplified patients with PARP inhibitors.

## SUPPORTING INFORMATION ON THE INTERNET

The following supporting information may be found in the online version of this article.

Figure S1 Flow diagram of the study design. **Figure S2**. Identification and validation of *PIK3CA* mutations by the Sequenom OncoCarta v1.0 panel. **Figure S3**. Validation of high-confidence fusion genes. **Figure S4**. Recurrence RT-PCR screen for nominated fusions in 14 micropapillary and 14 grade- and ER-matched IC-NSTs. **Figure S5**. Reconstitution of CDK12 in MDA-MB-453 with olaparib. **Figure S6**. Assessment of RAD51 foci formation in MCF7 cells with CDK12 silencing. **Table S1**. Details of the validation cohort of breast cancers. **Table S2**. Summary of antibodies, clones, dilutions, antigen retrieval methods, scoring, and cut-offs used for immunohistochemistry. **Table S3**. List of 273 genes included in the targeted capture massively parallel sequencing platform. **Table S4**. Breast cancer cell line characteristics. **Table S5**. Chromosomal regions of recurrent copy number changes in 16 MPCs, and the protein coding genes, microRNAs, and copy number variations mapping to these regions. **Table S6**. Summary of mRNA-sequencing statistics. **Table S7**. Recurrent alterations identified through re-analysis of public datasets.
