# Supplementary material for: Characterization of the genomic features and expressed fusion genes in micropapillary carcinomas of the breast
Source: J Pathol. 2014 Feb 5;232(5):553–65. doi: 10.1002/path.4325 (PMC4013428; doi:10.1002/path.4325)
Supplement: Figure S1 — Flow diagram of the study design. [file path0232-0553-SD1.pdf]

SUPPLEMENTARY INFORMATION

(ONLINE SUPPORTING INFORMATION)

Characterization of the genomic features and expressed fusion genes in micropapillary carcinomas of the breast

Natrajan et al.

Supplementary Methods

Supplementary Figures S1-S6

Supplementary Tables S1-S7

SUPPLEMENTARY METHODS

Tumor samples

Two cohorts of micropapillary carcinomas (MPCs) were analyzed; the first cohort comprised 16 consecutive formalin fixed paraffin embedded (FFPE) MPCs, 11 pure and 5 mixed, which were retrieved from the authors' institutions (Table 1), and a second, validation cohort comprised 14 additional consecutive FFPE MPCs, retrieved from Molinette Hospital, Turin, Italy. Frozen samples were available from five out of the 16 cases from the first cohort of MPCs.

As a comparator for the results of the Sequenom mutation profiling, a cohort of 16 consecutive IC-NSTs matched to the first cohort of 16 MPCs according to ER and HER2 status and histological grade were retrieved from a series of breast cancers previously analyzed by aCGH[1]. In addition, 14 IC-NSTs matched according to grade, and ER and HER2 status to tumors from the second cohort of 14 MPCs, and 48 grade 3 IC-NSTs were retrieved from Hospital La Paz, Madrid, Spain[1] (Supplementary Table S1).

Power calculation

For power calculations, we have assumed that if MPCs were driven by a recurrent fusion gene in a way akin to secretory carcinomas (which harbor the *ETV6-NTRK3* fusion gene in >95% of cases[2-4]) or adenoid cystic carcinomas of the breast (which harbor the *MYB-NFIB* fusion gene in >90% of cases[5]), a 'pathognomonic' driver event would be present in at least ≥70% of cases (an estimate that is conservative). Therefore, based on a binomial distribution, by sequencing 5 samples we would have been able to identify a recurrent event (i.e. in two or more cases) with 97% statistical power. Furthermore, a 'pathognomonic' driver event present in 50% of cases would be detectable with 80% power.

Immunohistochemical analysis

Representative sections of each case were cut at 3µm and mounted on silane-coated slides. Immunohistochemistry was performed as previously described [6,7], using antibodies raised against estrogen receptor (ER), progesterone receptor (PR), HER2, and epithelial membrane antigen (EMA). Antibody clones, dilution, antigen retrieval methods, scoring systems, and cut-offs [8,9] are summarized in Supplementary Table 2.

Positive and negative controls (omission of the primary antibody and IgG-matched serum) were included for each immunohistochemical run. The scoring was performed by at least two pathologists (CM, AS and/ or JSR-F).

Microdissection, DNA extraction, RNA extraction

Representative 8μm-thick sections of the MPCs and IC-NSTs were subjected to microdissection with a sterile needle under a stereomicroscope (Olympus SZ61, Tokyo, Japan) to ensure a percentage of tumor cells greater than 90%, as previously described[7,10]. DNA was extracted from the 16 MPCs and 16 grade-, ER-, and HER2-matched IC-NSTs using the DNeasy Blood and Tissue Kit (Qiagen, Crawley, UK). In addition, for 8 MPCs matched adjacent normal breast tissue was microdissected and subjected to DNA extraction. Double-stranded DNA concentration and DNA quality were determined using a Qubit Fluorometer (Invitrogen, Paisley, UK) and a multiplex PCR, respectively, as previously described[6,10]. RNA was extracted from the microdissected MPCs and IC-NSTs FFPE samples using the RNeasy kit (Qiagen). Frozen samples from five MPCs and 48 grade 3 IC-NSTs were cut, microdissected, and subjected to RNA extraction with Trizol (Invitrogen), according to the manufacturer's protocol. RNA quantity and quality was assessed using Agilent 2100 Bioanalyzer with RNA Nano LabChip Kits (Agilent Biosystems, Stockport, UK).

Microarray comparative genomic hybridization (aCGH)

aCGH data were pre-processed and analyzed using the Base.R script in R version 2.14.0, as previously described[1,11]. Genomic DNA from each sample was hybridized against a pool of normal female DNA derived from peripheral blood. Raw Log_2 ratios of intensity between samples and pooled female genomic DNA were read without background subtraction and normalized in the LIMMA package in R using PrinTipLoess. Outliers were removed based upon their deviation from neighboring genomic probes, using an estimation of the genome-wide median absolute deviation of all probes. Log_2 ratios were rescaled using the genome wide median absolute deviation in each sample and then smoothed using circular binary segmentation (cbs) in the DNACopy package [12-15]. After filtering polymorphic BACs and BACs mapping to chromosome Y, a final dataset of 31,157 clones with unambiguous mapping information according to build hg19 of the human genome (http://www.ensembl.org). A categorical analysis was applied to the BACs after classifying them as representing amplification (>0.45), gain (>0.08 and

≤0.45), loss (<−0.08) or no change, according to their cbs-smoothed log2 ratio values [7,14]. Threshold values were determined and validated as previously described [13,14].

Mutation screening and validation

Sixteen MPCs and 16 grade-, ER- and HER2-matched IC-NSTs were subjected to mutation screening using the OncoCarta Panel v 1.0 (Sequenom, San Diego, CA, USA), detecting 238 mutations in 19 common cancer-related genes, as previously described[12,15]. The prevalence of mutant alleles was estimated by calculating the ratio of the area of the raw spectra of the mutant allele to its wild-type allele. Mutations were validated using Sanger sequencing as previously described[12,15]. Primer sequences are available at http://rock.icr.ac.uk/collaborations/Mackay/Micropapillary. Sequences were visualized using 4Peaks (http://4peaks.en.softonic.com/).

Eight MPCs, from which both primary tumor and normal breast tissue could be microdissected, were subjected to microdissection, and DNA was extracted as previously described[7,10]. Tumor and germline DNA were subjected to targeted capture massively parallel sequencing using a platform containing baits targeting all exons of 273 genes that were either recurrently mutated in breast cancer or are involved in DNA repair pathways (Supplementary Table S3). Custom oligonucleotides (NimblegenSeqCap) were designed for hybridization capture of all protein-coding exons of 273 genes (Supplementary Table S3). Barcoded sequence libraries were prepared (New England Biolabs, KapaBiosystems) using 50ng DNA and pooled at equimolar concentrations into a single exon capture reaction as previously described[16,17]. Sequencing was performed in a single lane of an Illumina HiSeq2000 (San Diego, CA), and reads were aligned to the reference human genome hg19 using the Burrows-Wheeler Aligner (BWA) [6,18]. Somatic single nucleotide variants were identified using a combination of muTect[19], MutationSeq[20], Haplotypecaller[21] and VarScan2[22]; only single nucleotide variations identified by at least two out of the four callers employed were considered as valid. This conservative approach has been employed to minimize false positive results obtained with high depth targeted massively parallel sequencing performed with DNA extracted from formalin-fixed, paraffin-embedded tissues. For small insertions and deletions (indels), Haplotype Caller and VarScan2 were employed. All candidate mutations were reviewed manually using the Integrative Genomics Viewer[23]. Mutations with allelic frequency of <1% and/ or supported by <5

reads were disregarded. Regions with loss of heterozygosity were identified using exomeCNV[24].

Paired-end massively parallel RNA sequencing

Briefly, messenger RNA was selected using oligo-dT magnetic beads from 3μg of total tumor RNA. RNA was fragmented at 94°C for 5 minutes in fragmentation buffer (Illumina) and converted to single stranded cDNA using SuperScript II reverse transcriptase, followed by second-strand cDNA synthesis using Escherichia coli DNA polymerase I. Double-stranded cDNA was end repaired by using T4 DNA polymerase and T4 polynucleotide kinase; monoadenylated using a Klenow DNA polymerase I (3'-5' exonucleotide activity), and adapters ligated using T4 DNA ligase. The adaptor-ligated cDNA library was then fractioned on a 2% agarose gel, and a smear corresponding to 200 nucleotides was excised, purified, and PCR amplified using high fidelity Phusion DNA polymerase (Finnenzymes, Vantaa, Finland). The library was quantified using the Agilent 1000 kit on the Agilent 2100 Bioanalyzer following the manufacturer's instructions. 3pM was then loaded onto each lane of a flowcell, and sequencing performed with and 2x 54bp cycles on the Genome Analyser II.

Reverse-transcription PCR (RT-PCR), PCR and Sanger sequencing validation

Breakpoints of validated in-frame fusion genes were verified at the genomic level using the Expand Long Template PCR system (Roche Diagnostics Ltd, Burgess Hill, UK). For each fusion gene pair, the maximum size of intervening intronic sequence was calculated and primers were designed for the amplicon to encompass the fusion junction. Reverse transcription was performed with Superscript III (Invitrogen) using 500ng of RNA per reaction, according to the manufacturer's instructions. RT-PCR was performed using AmpliTaq Gold (Applied Biosystems, Warrington, UK) as previously described [25]. Products were sequenced using a DNA Sequencing Kit BigDye Terminator v 1.1 Cycle Sequencing Ready Reaction Mix (Applied Biosystems), as previously described[13]. Sequences were visualized using 4Peaks (http://4peaks.en.softonic.com/).

Validated in-frame fusion genes (i.e. *SLC2A1-FAF1* and *BCAS4-AURKA*) and out-of-frame fusion genes with recurrent partners found in independent datasets [26-29](i.e. *ELMO2-RAE1* and *LASP1-CDK12*) were screened at the cDNA level in an independent

cohort of MPCs (n=14), grade- and ER-matched IC-NSTs (n=14) and grade 3 IC-NSTs (n=48) using RT-PCR. Quantitative real-time PCR (qRT-PCR) assays for *BCAS4-AURKA* and *SLC2A1-FAF1* were performed as previously described[27], using SYBR green master mix (Applied Biosystems) and run on the 7900 Real time PCR device (Applied Biosystems). Relative mRNA levels were normalized to the expression of β-actin as previously described[27].

Cell line models

The ER-positive breast cancer cell lines, MCF7, BT474, T47D and ZR75.1 were obtained from ATCC (LGC Standards, Teddington, UK). The *HER2*-amplified cell lines JIMT1, UACC812, UACC893, VP229, SKBR3, ZR75.30, HCC1569, HCC1954, MDA-MB-453, MDA-MB-361 were obtained from ATCC and the cell lines SUM225, SUM190 were obtained from Asterand, Herts, UK). All cell lines were and maintained as previously described[30,31]. Cell lines were authenticated using short tandem repeat (STR) genotyping using the PowerPlex 1.2 STR system (Promega UK, Southampton, UK), according to the manufacturer's instructions.

Functional assessment of in-frame fusion genes

The *SLC2A1-FAF1* and *BCAS4-AURKA* fusion open reading frames (ORFs) were PCR amplified from primary tumor MPC10 and cloned into a mammalian expression vector pCMVentry, with a C-terminal DDK tag (Origene). Cloned fusions, full-length 3-prime partner gene constructs or empty vectors were transfected into in four ER-positive breast cancer cell lines (MCF7, BT474, T47D and ZR75.1) using Lipofectamine 2000 (Invitrogen). Antibiotic selection using geneticin (Invitrogen) was performed 48 hours after transfection for 5-7 days, before cells were then seeded in 96 well plates for growth assays. Cell population was estimated every 24 hours, for 9 days, using the CellTiter-Glo® cell viability assay (Promega), as previously described[32], with each reading normalized to day 1 to determine the fold change. Western blotting was performed to confirm expression of DDK in transfected cell lines prior to performing growth assays as previously described[32].

Analysis of RAD51 foci formation

In brief, cells were grown on coverslips (12mm, Thermo Scientific) and exposed to 10 Gray (Gy) of ionizing radiation. After 4-6 hours recovery, cells were fixed, permeabilized,

and immunostained with primary antibodies targeting RAD51 (polyclonal rabbit antibody, Santa Cruz) and detected with alexa fluor 488 (Invitrogen). Nuclei were counterstained with 4′,6-diamidino-2- phenylindole (DAPI). The presence of RAD51 foci was evaluated in a minimum of 100 cells in three independent experiments with an automated inverted fluorescence microscope (LEICA – DMI6000 B). RAD51 foci formation was scored as a percentage of cells with phospho-γ-H2AX foci formation without treatment, 6 hours after 10 Gy of irradiation, and with 1nM BMN673 for 24 hours. A minimum of 100 cells was counted to determine RAD51 focus formation frequency in three independent experiments.

Long-term survival assay

Long-term survival assays were performed as previously described[32,33]. In brief, cells were seeded in six-well plates in triplicates at a concentration of 1000-2000 per well and treated with the inhibitor after 24 hours, being continuously exposed to the drug (10^{-11} to 10^{-7} M) dissolved in dimethyl sulfoxide (DMSO) (control: DMSO). Media and drug were replaced every 3 days. After 14 days, cells were fixed with 10% tri-chloro-acetic acid (TCA) and stained with sulforhodamine B (SRB) (Sigma) and a colorimetric assay was performed as described previously[32,33]. The PARP inhibitor BMN673 was a kind gift from BioMarin Pharmaceuticals, USA, and olaparib (AZD2281/KU0058948) was obtained from SelleckBio (Munich, Germany).

Statistical analyses

Growth curves and drug sensitivity curves were constructed using non-linear regression (curve fit) analyses in Prism v5.04 (Graphpad Software Inc, La Jolla, CA, USA). Mann-Whitney U test was employed to compare the mean survival fractions of cell lines transfected with siRNAs targeting an out-of-frame fusion gene partner with those of cell lines transfected with control siRNA. One-way ANOVA tests, with a Bonferroni correction for multiple testing, were used to compare the fold-change in cell population on the final day of each growth assay for cancer cells transfected with a fusion gene construct, full length 3’ partner or empty vector. For all tests, two-tailed p values <0.05 were considered significant.

REFERENCES

1. Natrajan R, Weigelt B, Mackay A*, et al.* An integrative genomic and transcriptomic analysis reveals molecular pathways and networks regulated by copy number aberrations in basal-like, HER2 and luminal cancers. *Breast Cancer Res Treat* 2010; **121**: 575-589.
2. Tognon C, Knezevich SR, Huntsman D*, et al.* Expression of the ETV6-NTRK3 gene fusion as a primary event in human secretory breast carcinoma. *Cancer Cell* 2002; **2**: 367-376.
3. Weigelt B, Geyer FC, Reis-Filho JS. Histological types of breast cancer: how special are they? *Mol Oncol* 2010; **4**: 192-208.
4. Weigelt B, Reis-Filho JS. Histological and molecular types of breast cancer: is there a unifying taxonomy? *Nat Rev Clin Oncol* 2009; **6**: 718-730.
5. Wetterskog D, Lopez-Garcia MA, Lambros MB*, et al.* Adenoid cystic carcinomas constitute a genomically distinct subgroup of triple-negative and basal-like breast cancers. *J Pathol* 2012; **226**: 84-96.
6. Marchio C, Iravani M, Natrajan R*, et al.* Mixed micropapillary-ductal carcinomas of the breast: a genomic and immunohistochemical analysis of morphologically distinct components. *J Pathol* 2009; **218**: 301-315.
7. Marchio C, Iravani M, Natrajan R*, et al.* Genomic and immunophenotypical characterization of pure micropapillary carcinomas of the breast. *J Pathol* 2008; **215**: 398-410.
8. Harvey JM, Clark GM, Osborne CK*, et al.* Estrogen receptor status by immunohistochemistry is superior to the ligand-binding assay for predicting response to adjuvant endocrine therapy in breast cancer. *J Clin Oncol* 1999; **17**: 1474-1481.
9. Jacobs TW, Gown AM, Yaziji H*, et al.* Specificity of HercepTest in determining HER-2/neu status of breast cancers using the United States Food and Drug Administration-approved scoring system. *J Clin Oncol* 1999; **17**: 1983-1987.
10. Marchio C, Natrajan R, Shiu KK*, et al.* The genomic profile of HER2-amplified breast cancers: the influence of ER status. *J Pathol* 2008; **216**: 399-407.
11. Mackay A, Tamber N, Fenwick K*, et al.* A high-resolution integrated analysis of genetic and expression profiles of breast cancer cell lines. *Breast Cancer Res Treat* 2009; **118**: 481-498.

12. Duprez R, Wilkerson PM, Lacroix-Triki M*, et al.* Immunophenotypic and genomic characterization of papillary carcinomas of the breast. *J Pathol* 2012; **226**: 427-441.

13. Geyer FC, Weigelt B, Natrajan R*, et al.* Molecular analysis reveals a genetic basis for the phenotypic diversity of metaplastic breast carcinomas. *J Pathol* 2010; **220**: 562-573.

14. Natrajan R, Lambros MB, Rodriguez-Pinilla SM*, et al.* Tiling path genomic profiling of grade 3 invasive ductal breast cancers. *Clin Cancer Res* 2009; **15**: 2711-2722.

15. Hernandez L, Wilkerson PM, Lambros MB*, et al.* Genomic and mutational profiling of ductal carcinomas in situ and matched adjacent invasive breast cancers reveals intra-tumour genetic heterogeneity and clonal selection. *J Pathol* 2012.

16. Wagle N, Berger MF, Davis MJ*, et al.* High-throughput detection of actionable genomic alterations in clinical tumor samples by targeted, massively parallel sequencing. *Cancer Discov* 2012; **2**: 82-93.

17. De Mattos-Arruda L, Bidard FC, Won HH*, et al.* Establishing the origin of metastatic deposits in the setting of multiple primary malignancies: The role of massively parallel sequencing. *Mol Oncol* 2013.

18. DePristo MA, Banks E, Poplin R*, et al.* A framework for variation discovery and genotyping using next-generation DNA sequencing data. *Nat Genet* 2011; **43**: 491-498.

19. Cibulskis K, Lawrence MS, Carter SL*, et al.* Sensitive detection of somatic point mutations in impure and heterogeneous cancer samples. *Nat Biotechnol* 2013; **31**: 213-219.

20. Ding J, Bashashati A, Roth A*, et al.* Feature-based classifiers for somatic mutation detection in tumour-normal paired sequencing data. *Bioinformatics* 2012; **28**: 167-175.

21. McKenna A, Hanna M, Banks E*, et al.* The Genome Analysis Toolkit: a MapReduce framework for analyzing next-generation DNA sequencing data. *Genome Res* 2010; **20**: 1297-1303.

22. Koboldt DC, Zhang Q, Larson DE*, et al.* VarScan 2: somatic mutation and copy number alteration discovery in cancer by exome sequencing. *Genome Res* 2012; **22**: 568-576.

23. Robinson JT, Thorvaldsdottir H, Winckler W*, et al.* Integrative genomics viewer. *Nat Biotechnol* 2011; **29**: 24-26.

24. Sathirapongsasuti JF, Lee H, Horst BA*, et al.* Exome sequencing-based copy-number variation and loss of heterozygosity detection: ExomeCNV. *Bioinformatics* 2011; **27**: 2648-2654.

25. Ha KC, Lalonde E, Li L*, et al.* Identification of gene fusion transcripts by transcriptome sequencing in BRCA1-mutated breast cancers and cell lines. *BMC Med Genomics* 2011; **4**: 75.

26. Banerji S, Cibulskis K, Rangel-Escareno C*, et al.* Sequence analysis of mutations and translocations across breast cancer subtypes. *Nature* 2012; **486**: 405-409.

27. Robinson DR, Kalyana-Sundaram S, Wu YM*, et al.* Functionally recurrent rearrangements of the MAST kinase and Notch gene families in breast cancer. *Nat Med* 2011; **17**: 1646-1651.

28. Stephens PJ, McBride DJ, Lin ML*, et al.* Complex landscapes of somatic rearrangement in human breast cancer genomes. *Nature* 2009; **462**: 1005-1010.

29. Network TCGA. Comprehensive molecular portraits of human breast tumours. *Nature* 2012; **490**: 61-70.

30. Brough R, Frankum JR, Sims D*, et al.* Functional Viability Profiles of Breast Cancer. *Cancer Discov* 2011; **1**: 260-273.

31. Shiu KK, Wetterskog D, Mackay A*, et al.* Integrative molecular and functional profiling of ERBB2-amplified breast cancers identifies new genetic dependencies. *Oncogene* 2013.

32. Wilkerson PM, Dedes KJ, Wetterskog D*, et al.* Functional characterization of EMSY gene amplification in human cancers. *J Pathol* 2011; **225**: 29-42.

33. Dedes KJ, Wetterskog D, Mendes-Pereira AM*, et al.* PTEN deficiency in endometrioid endometrial adenocarcinomas predicts sensitivity to PARP inhibitors. *Sci Transl Med* 2010; **2**: 53ra75.

SUPPLEMENTARY FIGURES

Supplementary Figure S1.

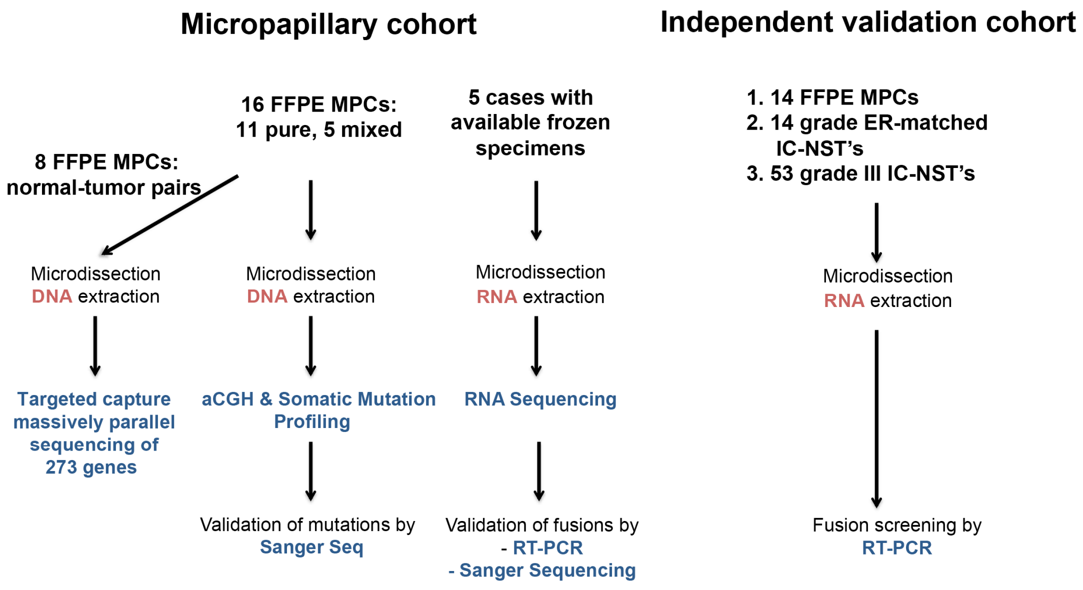

Supplementary Figure S1. Flow diagram of the study design.

Supplementary Figure S2.

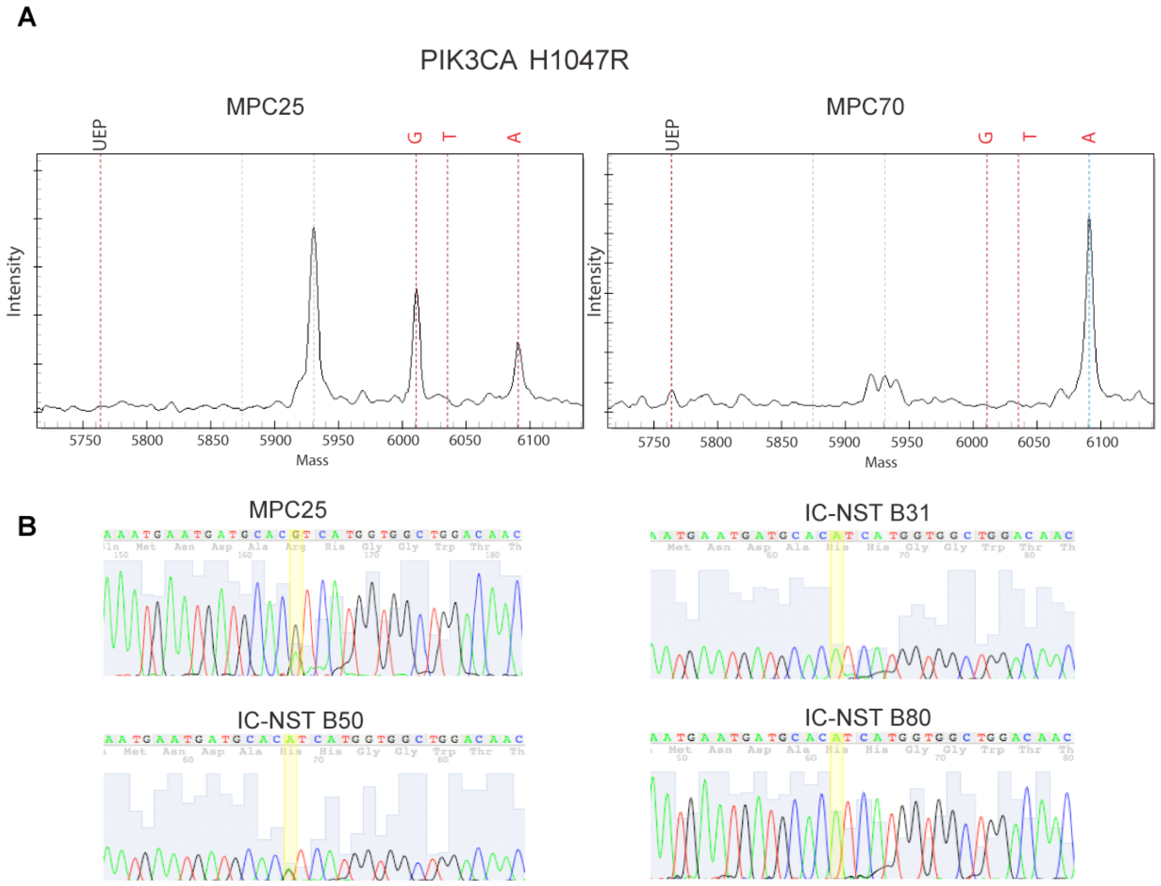

Supplementary Figure S2. Identification and validation of *PIK3CA* mutations by the Sequenom Oncocarta v1.0 panel. Using the Oncocarta v1.0 panel on the Sequenom platform, mutations were identified in a single micropapillary carcinoma (MPC25). **A**) Mass spectrometry profile illustrating the allele called in MPC25 and in the *PIK3CA* wild-type (WT) MPC70. **B**) Validation and identification of *PIK3CA* H1047R using conventional Sanger sequencing in MPC25 and in B50, the only case of the series of 16 grade-, ER-, and HER2-matched invasive ductal carcinomas of no special type (IC-NST) harboring a *PIK3CA* gene mutation. By comparison no mutations were identified in IC-NSTs B31 and B80.

Supplementary Figure S3

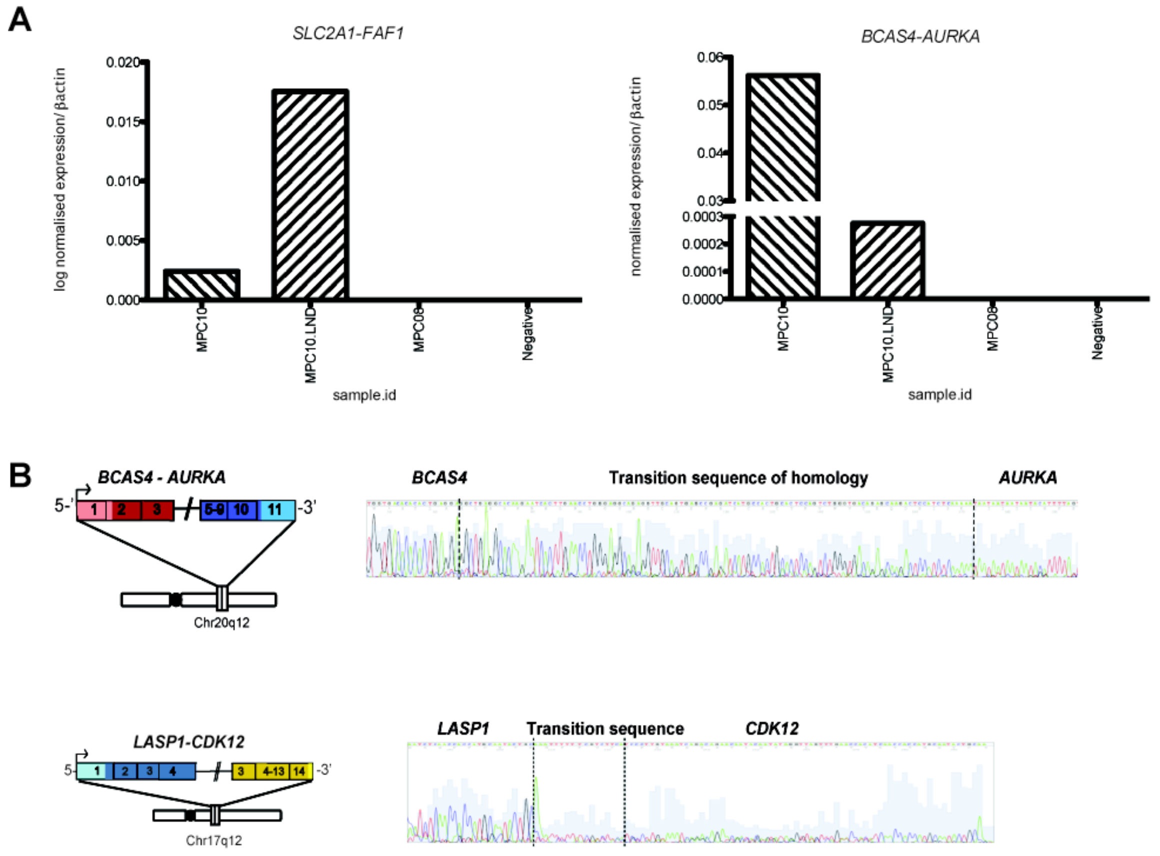

Supplementary Figure S3. Validation of high-confidence fusion genes.

A) Real-time reverse transcription PCR (qRT-PCR) analysis of validated in-frame fusion genes in selected cases. qRT-PCR of *SLC2A1-FAF1* and *BCAS4-AURKA*. MPCLND = lymph node metastasis of MPC10. Expression of fusion transcripts are normalized to the expression of β-actin. **B**) Long-range PCR validation of the DNA breakpoints of *BCAS4-AURKA*, *ELMO2-RAE1* and *LASP1-CDK12* in the index cases. Sanger sequencing validation of the DNA breakpoints of *BCAS4-AURKA* in MPC10 and *LASP1-CDK12* in MPC70. A schematic representation of nominated fusion transcripts is shown on the left. Fusion junctions with respective exon numbers are shown. Light colors represent 3' and 5' UTRs. Sequence chromatograms spanning the junction (dotted line) of the fusion transcript are shown on the right.

Supplementary Figure S4

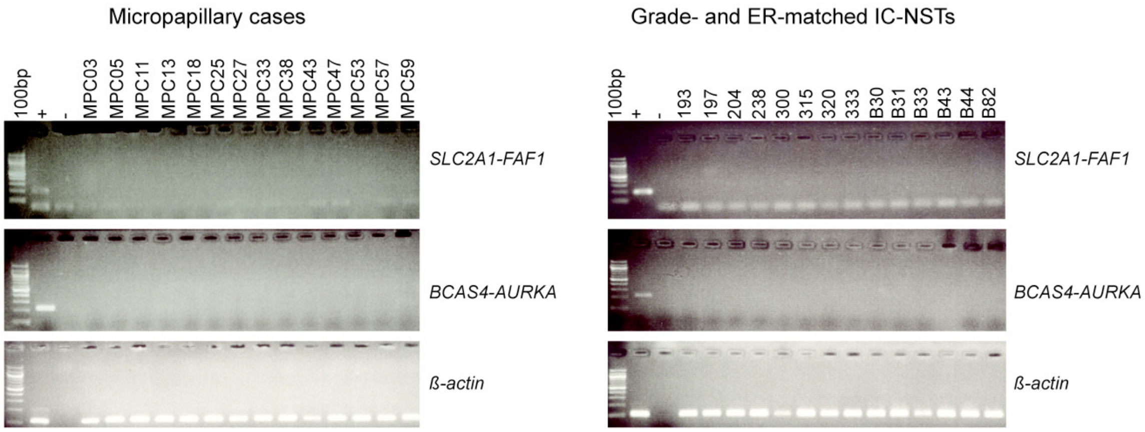

Supplementary Figure S4. Recurrence RT-PCR screen for nominated fusions in 14 micropapillary and 14 grade- and ER-matched IC-NST.

To determine if the nominated fusion genes (in-frame fusions) were recurrent, 14 micropapillary (MPCs) and 14 grade- and ER-matched invasive carcinomas of no special type (IC-NSTs) were screened with primers for *SLC2A1-FAF1*, *BCAS4-AURKA*, and β-actin as a control. Positive (index case) and negative (no template) controls were included in each run.

Supplementary Figure S5

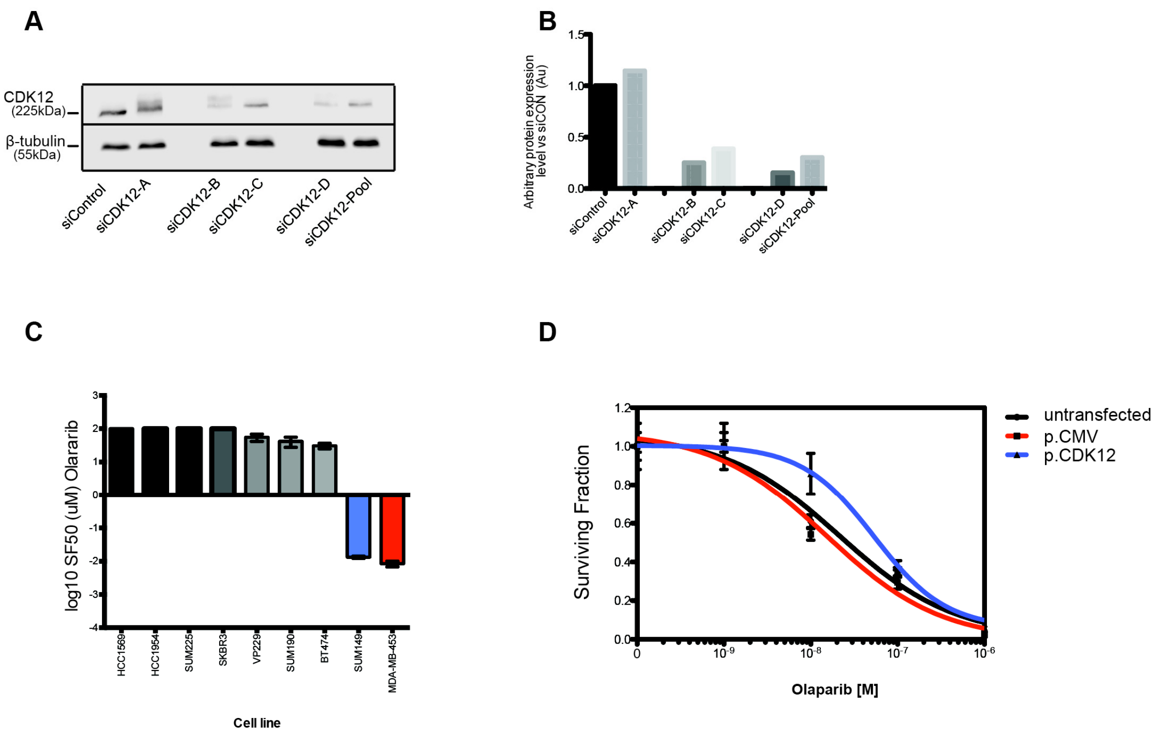

Supplementary Figure S5. Reconstitution of CDK12 in MDA-MB-453 with olararib. **A**) Western blot analysis of siRNA against CDK12 and **B**) quantification, showing 3 out of 4 oligos and the pool reduce CDK12 protein expression. **C**) Bar plot of $\log_{10}$ (SF50) of *HER2*-amplified cell lines to the PARP inhibitor olaparib. SUM149 (BRCA1 mutant) was used as a positive control (blue). MDA-MB-453 are sensitive to olaparib (red). **D**) Reconstitution of full length CDK12 (p.CDK12) in MDA-MB-453 results in increased resistance to olaparib (blue) relative to empty vector (red) (p.CMV) and untransfected cells (black).

Supplementary Figure S6

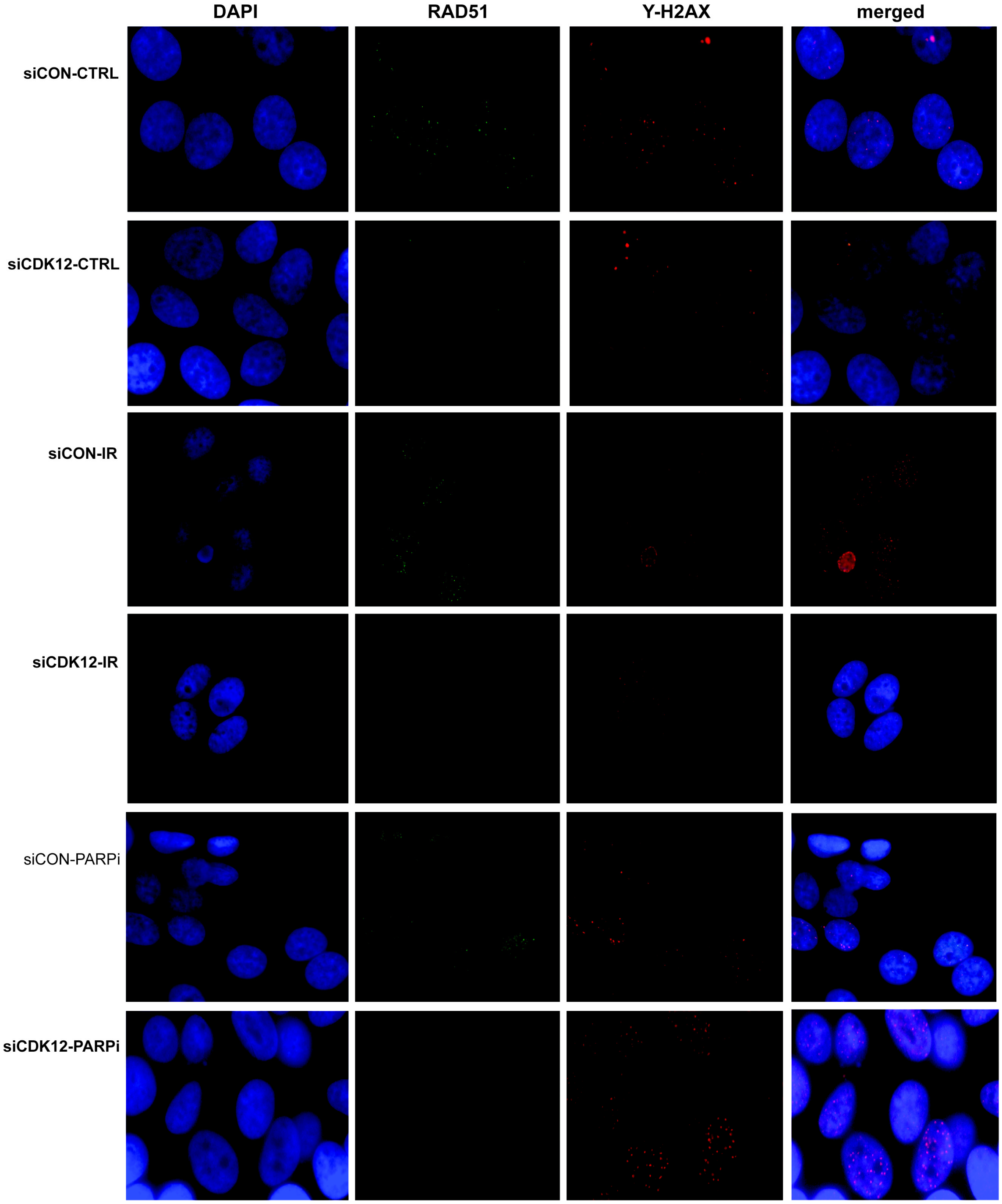

Supplementary Figure S6. Assessment of RAD51 foci formation in MCF7 cells with CDK12 silencing. Immunofluorescence images of representative fields of MCF7 cells with si-control (siCON) cells or CDK12-silenced (siCDK12) cells using a pool of four individual oligos showing DAPI-stained nuclei (blue), γ-H2AX (red), and RAD51 foci (green) after no treatment (CTRL), 10 Gy of irradiation (IR) or 24 hour treatment with 1μM of the PARP inhibitor BMN673.

Supplementary Table S1. Details of the complete cohort of breast cancers

Sample ID	Histology	Pure/ Mixed	Histological grade	ER	PR	HER2	aCGH	Sequenom	PIK3CA H1047R mutation screen	RNA-sequencing	RT-PCR fusion screen screen				
											Tested	*BCAS4-AURKA*	*SLC2A1-FAF1*	*ELMO2-RAE1*	*LASP1-CDK12*
MPC10	Micropapillary	pure	3	Positive	Positive	Negative	Y	Y	WT	Y	N	Y	Y	Y	N
MPC08	Micropapillary	pure	2	Positive	Positive	Negative	Y	Y	WT	Y	N	N	N	N	N
MPC70	Micropapillary	pure	2	Positive	Positive	Negative	Y	Y	WT	Y	N	N	N	N	N
MPC72	Micropapillary	pure	3	Positive	Negative	Negative	Y	Y	WT	Y	N	N	N	N	N
MPC71	Micropapillary	pure	3	Positive	Positive	Positive	Y	Y	WT	Y	N	N	N	N	Y
MPC37	Micropapillary	mixed	3	Positive	Positive	Negative	Y	Y	WT	N	N	NA	NA	NA	NA
MPC59	Micropapillary	mixed	2	Positive	Positive	Negative	N	N	NA	N	Y	N	N	N	N
MPC27	Micropapillary	mixed	2	Positive	Positive	Negative	N	N	NA	N	Y	N	N	N	N
MPC33	Micropapillary	mixed	2	Positive	Positive	Positive	N	N	NA	N	Y	N	N	N	N
MPC03	Micropapillary	pure	2	Positive	Positive	Negative	N	N	NA	N	Y	N	N	N	N
MPC13	Micropapillary	pure	2	Positive	Positive	Negative	N	N	NA	N	Y	N	N	N	N
MPC43	Micropapillary	mixed	2	Positive	Positive	Negative	N	N	NA	N	Y	N	N	N	N
MPC11	Micropapillary	pure	2	Positive	Positive	Negative	Y	Y	WT	N	Y	N	N	N	N
MPC25	Micropapillary	mixed	2	Positive	Positive	Negative	Y	Y	H1047R	N	Y	N	N	N	N
MPC57	Micropapillary	mixed	3	Positive	Positive	Negative	N	N	NA	N	Y	N	N	N	N
MPC05	Micropapillary	pure	3	Positive	Positive	Negative	N	N	NA	N	Y	N	N	N	N
MPC47	Micropapillary	mixed	3	Positive	Positive	Negative	N	N	NA	N	Y	N	N	N	N
MPC18	Micropapillary	pure	3	Positive	Positive	Negative	N	N	NA	N	Y	N	N	N	N
MPC38	Micropapillary	mixed	3	Positive	Positive	Negative	Y	Y	WT	N	Y	N	N	N	N
MPC53	Micropapillary	mixed	3	Negative	Negative	Positive	Y	Y	WT	N	Y	N	N	N	N
MPC07	Micropapillary	pure	3	Positive	Negative	Positive	N	N	NA	N	N	NA	NA	NA	NA
MPC19	Micropapillary	pure	3	Positive	Positive	Negative	N	N	NA	N	N	NA	NA	NA	NA
MPC16	Micropapillary	pure	3	Positive	Positive	Negative	N	N	NA	N	N	NA	NA	NA	NA
MPC06	Micropapillary	pure	2	Positive	Positive	Negative	Y	Y	WT	N	N	NA	NA	NA	NA
MPC02	Micropapillary	pure	2	Positive	Positive	Negative	Y	Y	WT	N	N	NA	NA	NA	NA
MPC04	Micropapillary	pure	3	Positive	Negative	Negative	Y	Y	WT	N	N	NA	NA	NA	NA
MPC01	Micropapillary	pure	2	Positive	Positive	Negative	Y	Y	WT	N	N	NA	NA	NA	NA
MPC23	Micropapillary	pure	3	Positive	Negative	Negative	Y	Y	WT	N	N	NA	NA	NA	NA
MPC45	Micropapillary	mixed	3	Positive	Positive	Positive	Y	Y	WT	N	N	NA	NA	NA	NA
BC2468	IC-NST	NA	2	Positive	Positive	Negative	N	Y	WT	N	N	NA	NA	NA	NA
BC2472	IC-NST	NA	2	Positive	Positive	Negative	N	Y	WT	N	N	NA	NA	NA	NA
BC2522	IC-NST	NA	2	Positive	Positive	Negative	N	Y	WT	N	N	NA	NA	NA	NA
BC2512	IC-NST	NA	2	Positive	Negative	Negative	N	Y	WT	N	N	NA	NA	NA	NA
BC2469	IC-NST	NA	2	Positive	Positive	Negative	N	Y	WT	N	N	NA	NA	NA	NA
BC2473	IC-NST	NA	2	Positive	Positive	Negative	N	Y	WT	N	N	NA	NA	NA	NA
BC2700	IC-NST	NA	2	Positive	Positive	Negative	N	N	NA	N	N	NA	NA	NA	NA
B1	IC-NST	NA	3	Positive	Negative	Negative	N	Y	WT	N	Y	N	N	N	N
B11	IC-NST	NA	3	Negative	Positive	Negative	N	N	NA	N	Y	N	N	N	N
B12	IC-NST	NA	3	Negative	Negative	Positive	N	N	NA	N	Y	N	N	N	N
B15	IC-NST	NA	3	Positive	Positive	Negative	N	N	NA	N	Y	N	N	N	N
B16	IC-NST	NA	3	Positive	Positive	Negative	N	Y	WT	N	Y	N	N	N	N
B17	IC-NST	NA	3	Negative	Negative	Negative	N	N	NA	N	Y	N	N	N	N
B2	IC-NST	NA	3	Negative	Negative	Negative	N	N	NA	N	Y	N	N	N	N
B20	IC-NST	NA	3	Negative	Negative	Positive	N	N	NA	N	Y	N	N	N	N
B21	IC-NST	NA	3	Positive	Positive	Negative	N	Y	WT	N	Y	N	N	N	N
B24	IC-NST	NA	3	Positive	Negative	Negative	N	Y	WT	N	Y	N	N	N	N
B26	IC-NST	NA	3	Positive	Negative	Positive	N	N	NA	N	Y	N	N	N	N
B27	IC-NST	NA	3	Positive	Positive	Negative	N	Y	WT	N	Y	N	N	N	N
B29	IC-NST	NA	3	Negative	Negative	Negative	N	N	NA	N	Y	N	N	N	N
B32	IC-NST	NA	3	Negative	Negative	Positive	N	N	NA	N	Y	N	N	N	N
B34	IC-NST	NA	3	Negative	Negative	Positive	N	N	NA	N	Y	N	N	N	N
B35	IC-NST	NA	3	Negative	Negative	Positive	N	N	NA	N	Y	N	N	N	N
B37	IC-NST	NA	3	Positive	Positive	Negative	N	N	NA	N	Y	N	N	N	N
B39	IC-NST	NA	3	Positive	Positive	Negative	N	N	NA	N	Y	N	N	N	N
B40	IC-NST	NA	3	Negative	Positive	Positive	N	N	NA	N	Y	N	N	N	N
B41	IC-NST	NA	3	Negative	Negative	Negative	N	N	NA	N	Y	N	N	N	N
B45	IC-NST	NA	3	Negative	Negative	Negative	N	N	NA	N	Y	N	N	N	N
B46	IC-NST	NA	3	Negative	Negative	Positive	N	N	NA	N	Y	N	N	N	N
B47	IC-NST	NA	3	Positive	Negative	Negative	N	N	NA	N	Y	N	N	N	N
B49	IC-NST	NA	3	Positive	Positive	Negative	N	N	NA	N	Y	N	N	N	N
B5	IC-NST	NA	3	Positive	Positive	Negative	N	N	NA	N	Y	N	N	N	N
B50	IC-NST	NA	3	Positive	Positive	Negative	N	Y	H1047R	N	N	NA	NA	NA	NA
B6	IC-NST	NA	3	Negative	Negative	Negative	N	N	NA	N	Y	N	N	N	N
B60	IC-NST	NA	3	Positive	Positive	Negative	N	Y	WT	N	Y	N	N	N	N
B61	IC-NST	NA	3	Positive	Positive	Negative	N	Y	WT	N	Y	N	N	N	N
B62	IC-NST	NA	3	Negative	Negative	Negative	N	N	NA	N	Y	N	N	N	N
B64	IC-NST	NA	3	Negative	Negative	Negative	N	N	NA	N	Y	N	N	N	N
B65	IC-NST	NA	3	Negative	Negative	Negative	N	N	NA	N	Y	N	N	N	N
B66	IC-NST	NA	3	Negative	Negative	Positive	N	N	NA	N	Y	N	N	N	N
B67	IC-NST	NA	3	Negative	Negative	Negative	N	N	NA	N	Y	N	N	N	N
B69	IC-NST	NA	3	Negative	Negative	Negative	N	N	NA	N	Y	N	N	N	N
B7	IC-NST	NA	3	Positive	Negative	Negative	N	N	NA	N	Y	N	N	N	N
B72	IC-NST	NA	3	Positive	Positive	Negative	N	N	NA	N	Y	N	N	N	N
B74	IC-NST	NA	3	Positive	Negative	Negative	N	Y	WT	N	Y	N	N	N	N
B76	IC-NST	NA	3	Positive	Positive	Positive	N	N	NA	N	Y	N	N	N	N
B77	IC-NST	NA	3	Positive	Positive	Positive	N	N	NA	N	Y	N	N	N	N
B78	IC-NST	NA	3	Negative	Negative	Negative	N	N	NA	N	Y	N	N	N	N
B79	IC-NST	NA	3	Negative	Negative	Negative	N	N	NA	N	Y	N	N	N	N
B80	IC-NST	NA	3	Negative	Negative	Positive	N	N	NA	N	Y	N	N	N	N
B81	IC-NST	NA	3	Negative	Negative	Negative	N	N	NA	N	Y	N	N	N	N
B84	IC-NST	NA	3	Negative	Negative	Negative	N	N	NA	N	Y	N	N	N	N
B86	IC-NST	NA	3	Negative	Negative	Negative	N	N	NA	N	Y	N	N	N	N
B87	IC-NST	NA	3	Negative	Negative	Negative	N	N	NA	N	Y	N	N	N	N
B9	IC-NST	NA	3	Positive	Negative	Negative	N	Y	WT	N	Y	N	N	N	N
193	IC-NST	NA	2	Positive	Positive	Negative	N	N	NA	N	Y	N	N	N	N
197	IC-NST	NA	2	Positive	Positive	Negative	N	N	NA	N	Y	N	N	N	N
204	IC-NST	NA	2	Positive	Positive	Negative	N	N	NA	N	Y	N	N	N	N
238	IC-NST	NA	2	Positive	Positive	Negative	N	N	NA	N	Y	N	N	N	N
315	IC-NST	NA	2	Positive	Positive	Negative	N	N	NA	N	Y	N	N	N	N
320	IC-NST	NA	2	Positive	Positive	Negative	N	N	NA	N	Y	N	N	N	N
333	IC-NST	NA	2	Positive	Positive	Negative	N	N	NA	N	Y	N	N	N	N
300	IC-NST	NA	2	Positive	Positive	Positive	N	N	NA	N	Y	N	N	N	N
B30	IC-NST	NA	3	Positive	Positive	Negative	N	N	NA	N	Y	N	N	N	N
B31	IC-NST	NA	3	Positive	Positive	Negative	N	N	NA	N	Y	N	N	N	N
B33	IC-NST	NA	3	Positive	Positive	Negative	N	N	NA	N	Y	N	N	N	N
B44	IC-NST	NA	3	Positive	Positive	Negative	N	N	NA	N	Y	N	N	N	N
B82	IC-NST	NA	3	Positive	Positive	Negative	N	N	NA	N	Y	N	N	N	N
B43	IC-NST	NA	3	Negative	Negative	Positive	N	N	NA	N	Y	N	N	N	N

ER: estrogen receptor; PR: progesterone receptor; NA: not applicable; N: no; Y: yes.

Supplementary Table S2. Summary of antibodies, clones, dilutions, antigen retrieval methods, scoring and cut-offs used

Marker	Antibody clone	Dilution	Antigen retrival	Company	Scoring system	Cut-off
EMA	E29	1:100	None	DAKO, Glostrup, Denmark	Qualitative assessment of the positivity: membranous or cytoplasmic; lining the luminal or stroma-facing border of the cells ('inside out' pattern)	-
ER	ID5	1:200	2 min, PC citrate, pH 6.0	DAKO, Glostrup, Denmark	Allred scoring system	Positive: >2 (ref 3)
PR	Pgr 636	1:200	3 min, PC citrate, pH 6.0	DAKO, Glostrup, Denmark	Allred scoring system	Positive: >2 (3)
HER2	Polyclonal	1:1200	41 min water bath, DAKO antigen retrieval solution, pH 6.0	DAKO, Glostrup, Denmark	Score 0, 1, 2, 3 according to Hercep Test kit scoring guidelines	Positive ≥2 (4)

PC=Pressure cooker

Supplementary Table S3: List of 273 genes included in the targeted capture massively parallel sequencing platform.

Gene_name	Number of cases with mutation in the TCGA breast cancer study	% of cases with mutation in the TCGA breast cancer study	Number of cases with amplification of homozygous deletion in the TCGA breast cancer study	% of cases with amplification of homozygous deletion in the TCGA breast cancer study
ABCA13	14	2.80%	2	0.40%
ABCB1	3	0.60%	1	0.20%
ADAMTSL1	3	0.60%	4	0.80%
AGFG2	3	0.60%	4	0.80%
AHNAK2	9	1.80%	6	1.20%
AK9	9	1.80%	2	0.40%
AKAP9	4	0.80%	16	3.20%
AKT1	12	2.40%	6	1.20%
AKT2	1	0.20%	7	1.40%
AKT3	3	0.60%	24	4.70%
ANK3	10	2.00%	9	1.80%
AOAH	0	0.00%	1	0.20%
APC	3	0.60%	0	0.00%
APOB	15	3.00%	0	0.00%
APOBEC1	1	0.20%	7	1.40%
APOBEC2	1	0.20%	5	1.00%
APOBEC3A	1	0.20%	1	0.20%
APOBEC3C	0	0.00%	1	0.20%
APOBEC3D	0	0.00%	2	0.40%
APOBEC3F	0	0.00%	2	0.40%
APOBEC3G	1	0.20%	2	0.40%
APOBEC3H	0	0.00%	2	0.40%
APOBEC4	1	0.20%	20	3.90%
ARAF	1	0.20%	0	0.00%
ARID1A	11	2.20%	2	0.40%
ATM	16	3.20%	5	1.00%
ATN1	8	1.60%	8	1.60%
ATR	2	0.40%	4	0.80%
ATRX	9	1.80%	0	0.00%
AURKA	1	0.20%	20	3.90%
AURKB	0	0.00%	2	0.40%
AURKC	1	0.20%	12	2.40%
BIRC5	0	0.00%	21	4.10%
BIRC6	6	1.20%	2	0.40%
BRAF	3	0.60%	3	0.60%
BRCA1	15	3.00%	5	1.00%
BRCA2	22	4.30%	8	1.60%
BRIP1	8	1.60%	36	7.10%
CACNA1A	6	1.20%	6	1.20%
CACNA1C	6	1.20%	7	1.40%
CACNA1E	13	2.60%	19	3.70%
CBFB	8	1.60%	8	1.60%
CDC25A	1	0.20%	0	0.00%
CDC25B	3	0.60%	5	1.00%
CDC25C	1	0.20%	1	0.20%
CDH1	33	6.50%	10	2.00%
CDK1	0	0.00%	9	1.80%
CDK4	0	0.00%	10	2.00%
CDK6	1	0.20%	3	0.60%
CDKN1A	0	0.00%	3	0.60%
CDKN1B	5	1.00%	2	0.40%
CDKN2A	0	0.00%	23	4.50%
CDKN2B	0	0.00%	24	4.70%
CEP164	4	0.80%	0	0.00%
CFH	8	1.60%	23	4.50%
CHD4	7	1.40%	8	1.60%
CHD6	7	1.40%	6	1.20%
CHEK1	0	0.00%	2	0.40%
CHEK2	4	0.80%	3	0.60%
COL12A1	10	2.00%	3	0.60%
CROCCP2	0	0.00%	0	0.00%
CSMD1	15	3.00%	23	4.50%
CSMD2	10	2.00%	0	0.00%
CSMD3	9	1.80%	57	11.20%
CTCF	13	2.60%	8	1.60%
CTNNB1	0	0.00%	1	0.20%
CUBN	7	1.40%	6	1.20%
DCHS2	8	1.60%	3	0.60%
DEPTOR	0	0.00%	56	11.00%
DMC1	2	0.40%	2	0.40%
DOCK11	9	1.80%	0	0.00%
DSPP	7	1.40%	4	0.80%
DST	7	1.40%	6	1.20%
EGFR	4	0.80%	8	1.60%
EME1	0	0.00%	34	6.70%

EME2	0	0.00%	12	2.40%
EPPK1	1	0.20%	35	6.90%
ERBB2	7	1.40%	66	13.00%
ERBB3	8	1.60%	2	0.40%
ERBB4	6	1.20%	3	0.60%
ERCC1	1	0.20%	6	1.20%
ERCC2	0	0.00%	6	1.20%
ERCC3	2	0.40%	1	0.20%
ERCC5	4	0.80%	8	1.60%
ESR1	2	0.40%	10	2.00%
ESR2	2	0.40%	4	0.80%
FAM157B	1	0.20%	2	0.40%
FAM186A	4	0.80%	1	0.20%
FANCA	7	1.40%	13	2.60%
FANCB	0	0.00%	0	0.00%
FANCC	1	0.20%	2	0.40%
FANCD2	3	0.60%	7	1.40%
FANCE	1	0.20%	1	0.20%
FANCF	1	0.20%	6	1.20%
FANCG	1	0.20%	4	0.80%
FANCI	3	0.60%	10	2.00%
FANCL	1	0.20%	3	0.60%
FANCM	2	0.40%	3	0.60%
FAT3	20	3.90%	8	1.60%
FBN1	7	1.40%	3	0.60%
FCGBP	6	1.20%	9	1.80%
FGFR1	0	0.00%	54	10.70%
FGFR2	4	0.80%	9	1.80%
FGFR3	1	0.20%	5	1.00%
FGFR4	3	0.60%	5	1.00%
FLG	22	4.30%	34	6.70%
FMN2	8	1.60%	24	4.70%
FOXA1	8	1.60%	8	1.60%
FOXC2	0	0.00%	12	2.40%
FRG1B	0	0.00%	2	0.40%
FRMPD4	7	1.40%	0	0.00%
GATA3	54	10.70%	12	2.40%
GATA4	0	0.00%	16	3.20%
GPR98	11	2.20%	2	0.40%
GRB2	1	0.20%	20	3.90%
GRIN2A	10	2.00%	10	2.00%
GRIN2B	8	1.60%	3	0.60%
HECW1	8	1.60%	1	0.20%
HERC2	6	1.20%	5	1.00%
HIF1A	1	0.20%	4	0.80%
HMCN1	19	3.70%	20	3.90%
HRAS	0	0.00%	4	0.80%
HRNR	9	1.80%	34	6.70%
HSP90AA1	2	0.40%	4	0.80%
HSP90AB1	2	0.40%	6	1.20%
HUWE1	9	1.80%	0	0.00%
HYDIN	13	2.60%	10	2.00%
IGF1R	2	0.40%	21	4.10%
INPP4B	4	0.80%	6	1.20%
IRS1	1	0.20%	1	0.20%
JAK1	2	0.40%	6	1.20%
JAK2	5	1.00%	4	0.80%
KIT	5	1.00%	0	0.00%
KRAS	4	0.80%	7	1.40%
LAMA1	7	1.40%	5	1.00%
LAMA5	7	1.40%	20	3.90%
LRP2	17	3.40%	2	0.40%
MACF1	11	2.20%	1	0.20%
MAP1A	10	2.00%	1	0.20%
MAP2K1	1	0.20%	4	0.80%
MAP2K2	1	0.20%	7	1.40%
MAP2K3	1	0.20%	7	1.40%
MAP2K4	21	4.10%	11	2.20%
MAP2K6	0	0.00%	23	4.50%
MAP3K1	39	7.70%	4	0.80%
MAP3K10	4	0.80%	7	1.40%
MAP3K4	4	0.80%	4	0.80%
MAP4K4	1	0.20%	3	0.60%
MAPK1	1	0.20%	3	0.60%
MAPK8	2	0.40%	1	0.20%
MAPK9	0	0.00%	3	0.60%
MDM2	2	0.40%	19	3.70%
MDN1	11	2.20%	4	0.80%
MED12	8	1.60%	0	0.00%
MET	3	0.60%	0	0.00%
MGAM	6	1.20%	3	0.60%

MGMT	2	0.40%	6	1.20%
MLH1	1	0.20%	0	0.00%
MLH3	3	0.60%	3	0.60%
MLL2	8	1.60%	2	0.40%
MLL3	37	7.30%	1	0.20%
MRE11A	1	0.20%	9	1.80%
MSH2	2	0.40%	2	0.40%
MSH3	2	0.40%	1	0.20%
MSH4	1	0.20%	2	0.40%
MSH5	1	0.20%	1	0.20%
MSH6	4	0.80%	2	0.40%
MST1L	5	1.00%	0	0.00%
MTOR	8	1.60%	2	0.40%
MUTYH	2	0.40%	2	0.40%
MXRA5	7	1.40%	0	0.00%
MYH8	5	1.00%	2	0.40%
NBEAL2	6	1.20%	2	0.40%
NBN	2	0.40%	37	7.30%
NBPF1	1	0.20%	0	0.00%
NCOA3	6	1.20%	12	2.40%
NCOR1	17	3.40%	4	0.80%
NCOR2	3	0.60%	5	1.00%
NEB	19	3.70%	0	0.00%
NF1	14	2.80%	12	2.40%
NF2	2	0.40%	3	0.60%
NR1H2	3	0.60%	10	2.00%
NRAS	0	0.00%	2	0.40%
OBSCN	16	3.20%	21	4.10%
PALB2	0	0.00%	9	1.80%
PARP1	1	0.20%	22	4.30%
PARP2	1	0.20%	1	0.20%
PARP3	2	0.40%	1	0.20%
PCLO	6	1.20%	1	0.20%
PCNXL2	9	1.80%	24	4.70%
PDGFRA	3	0.60%	1	0.20%
PDGFRB	2	0.40%	2	0.40%
PGR	2	0.40%	7	1.40%
PIK3CA	178	35.10%	22	4.30%
PIK3CB	4	0.80%	5	1.00%
PIK3R1	13	2.60%	2	0.40%
PKHD1L1	12	2.40%	53	10.50%
PLEC	7	1.40%	35	6.90%
PLK1	2	0.40%	9	1.80%
PLXNA4	8	1.60%	1	0.20%
PMS1	5	1.00%	3	0.60%
PMS2	1	0.20%	3	0.60%
POLB	1	0.20%	37	7.30%
POLD1	0	0.00%	11	2.20%
POLE	5	1.00%	1	0.20%
POLH	2	0.40%	5	1.00%
POLQ	6	1.20%	2	0.40%
PRKCA	0	0.00%	36	7.10%
PRKCB	3	0.60%	9	1.80%
PRKCD	0	0.00%	1	0.20%
PRKCG	2	0.40%	11	2.20%
PTCH1	6	1.20%	2	0.40%
PTEN	18	3.60%	9	1.80%
PTK2	3	0.60%	37	7.30%
RAD50	2	0.40%	0	0.00%
RAD51	1	0.20%	3	0.60%
RAD51B	0	0.00%	1	0.20%
RAD51C	2	0.40%	33	6.50%
RAD51D	1	0.20%	10	2.00%
RAD52	0	0.00%	13	2.60%
RAD54B	4	0.80%	38	7.50%
RAD54L	2	0.40%	3	0.60%
RAF1	1	0.20%	4	0.80%
RB1	9	1.80%	7	1.40%
RBBP8	0	0.00%	3	0.60%
RELN	13	2.60%	1	0.20%
RICTOR	3	0.60%	6	1.20%
RPS6KB1	0	0.00%	47	9.30%
RPTOR	2	0.40%	14	2.80%
RUNX1	17	3.40%	3	0.60%
SHC1	1	0.20%	32	6.30%
SHROOM4	5	1.00%	0	0.00%
SMO	2	0.40%	3	0.60%
SOS1	4	0.80%	2	0.40%
SPEN	13	2.60%	0	0.00%
SPRY1	0	0.00%	4	0.80%
SPTA1	14	2.80%	24	4.70%

SRCAP	9	1.80%	15	3.00%
SSPO	15	3.00%	5	1.00%
STAT1	1	0.20%	4	0.80%
STAT3	1	0.20%	10	2.00%
SVEP1	11	2.20%	3	0.60%
SYNE1	17	3.40%	11	2.20%
SYNE2	17	3.40%	4	0.80%
TBX3	13	2.60%	1	0.20%
TENM1	10	2.00%	0	0.00%
TEX15	10	2.00%	14	2.80%
TGFBR1	2	0.40%	3	0.60%
TGFBR2	3	0.60%	0	0.00%
TGFBR3	2	0.40%	2	0.40%
TMC2	4	0.80%	2	0.40%
TOP2A	1	0.20%	24	4.70%
TP53	187	36.90%	3	0.60%
TP53BP1	3	0.60%	1	0.20%
TSC1	3	0.60%	2	0.40%
TSC2	2	0.40%	12	2.40%
TYK2	4	0.80%	6	1.20%
UBR4	12	2.40%	0	0.00%
USH2A	27	5.30%	21	4.10%
USP36	3	0.60%	19	3.70%
VWF	8	1.60%	7	1.40%
WDFY3	9	1.80%	3	0.60%
XBP1	2	0.40%	3	0.60%
XIRP2	12	2.40%	0	0.00%
XPA	0	0.00%	1	0.20%
XPC	0	0.00%	5	1.00%
XRCC1	1	0.20%	4	0.80%
XRCC2	0	0.00%	1	0.20%
XRCC3	0	0.00%	6	1.20%
ZFHX3	10	2.00%	9	1.80%
ZFHX4	16	3.20%	28	5.50%
ZNF384	0	0.00%	8	1.60%
ZNF703	0	0.00%	61	12.00%

Supplementary Table S4. Breast cancer cell line characteristics

	MCF7	BT474	T47D	ZR75.1	MDA-MB-361	HCC1954	MDA-MB-453	JIMT1	UACC812	UACC893	VP229	SKBR3	ZR75.30	SUM225	SUM190	HCC1569	SUM149
**ER**	Positive	Positive	Positive	Positive	Positive	Negative	Negative	Negative	Positive	Positive	Positive	Negative	Positive	Negative	Negative	Negative	Negative
**HER2**	Negative	Positive	Negative	Negative	Positive	Positive	Positive	Positive	Positive	Positive	Positive	Positive	Positive	Positive	Positive	Positive	Negative
**TP53**	Negative	Positive/ Mutant	Positive/ Mutant	Negative	Negative/ WT	Negative/ Mutant	Negative/ WT	NA	Negative/ WT	Mutant	Mutant	Positive/ Mutant	Negative/ WT	Positive/ Mutant	Negative/ Mutant	Negative/ Mutant	Positive/ Mutant
**1q gain**	Gain	Gain	Gain	Gain	NC	Gain	Gain	Gain	Gain	Gain	NC	Gain	Gain	Gain	Gain	Gain	Gain
**8q gain**	Gain	Gain	Gain	Gain	Gain	Gain	Gain	Gain	Gain	Gain	Gain	Gain	Gain	Gain	Gain	Gain	Gain
**17q gain**	Gain	Gain	Gain	Gain	Gain	Gain	Gain	Gain	Gain	Gain	Gain	Gain	Gain	Gain	Gain	NC	NC
**20q gain**	Gain	Gain	Gain	Gain	Gain	Gain	Gain	Gain	Gain	Gain	Gain	Gain	NC	Gain	NC	Gain	Gain
**1p loss**	Loss	Loss	Loss	Loss	Loss	Loss	NC	Loss	Loss	Loss	Loss	Loss	Loss	NC	NC	NC	NC
**8p loss**	Loss	Loss	Loss	Loss	Loss	Loss	Loss	Loss	Loss	Loss	Loss	Loss	Loss	Loss	Loss	Loss	Loss
**13q loss**	Loss	NA	Loss	NA	NC	NC	Loss	NC	Loss	Loss	Loss	NC	NC	NC	Loss	Loss	NC
**16q loss**	Loss	Loss	Loss	Loss	NC	Gain	Loss	Loss	Loss	Loss	NC	Loss	Loss	NC	NC	NC	Loss
**22q loss**	Loss	Loss	Loss	Loss	Loss	Loss	Gain	Loss	Loss	Loss	NC	Loss	NC	Loss	Loss	NC	Loss
RAE1	Amp	Amp/fusion	NC	NC	NC	Amp	NC	Amp	Amp	NC	Amp	Amp	NC	NC	NC	NC	Amp
ELMO2	NC	NC	NC	NC	NC	NC	NC	Amp	NC	NC	Amp	Amp	NC	NC	NC	Amp	Amp
BCAS4	Amp/fusion	Amp	NC	NC	NC	NC	NC	Amp	Amp	NC	Amp	NC	NC	NC	NC	NC	NC
AURKA	Amp	Amp	NC	NC	NC	Amp	NC	Amp	Amp	NC	Amp	Amp	NC	NC	NC	NC	Amp
SLC2A1	NC	NC	NC	NC	NC	NC	NC	NC	NC	NC	NC	NC	NC	NC	NC	NC	NC
FAF1	NC	NC	NC	NC	NC	NC	NC	NC	NC	NC	NC	NC	NC	NC	NC	NC	NC
CDK12	NC	Amp	NC	NC	Fusion /breakpoint	Fusion/ breakpoint	Breakpoint	No breakpoint	No breakpoint	No breakpoint	No breakpoint	No breakpoint	No breakpoint	Breakpoint	Breakpoint	Breakpoint	NC
LASP1	NC	Amp	NC	NC													
BRCA1	WT	WT	WT	WT	WT	WT	WT	WT	WT	WT	WT	WT	WT	WT	WT	WT	Mutant

Amp: amplification; fusion: fusion gene detected involving this gene; gain: chromosomal arm gain; loss: chromosomal arm loss; NC: no copy number change/alteration, not assessed breakpoint; breakpoint identified in gene from aCGH analysis.

Supplementary Table S5. Chromosomal regions of recurrent copy number changes in 16 MPCs, and the protein coding genes, microRNAs and copy number variations mapping to these regions mapping to these regions.

GAIN (in 3 or more cases)																
**Chrom**	**Start**	**End**	**Start MB**	**End MB**	**Cytobands**	**Start Probe**	**End Prob**	**Probes**	**Length MB**	**maxM**	**max.overlap**	**Cases**	**Genes**	**miRNAs**	**aCGH CNVs**	**Full CNVs**
1	1365313	3679535	1:37	0.17222222	p36.33-p36.32	TP0124F03	TP0114H0	25	2,314,222	0.385086463316099	5	C10, C11, C17 , C18, C24	VWA1, ATAD3C, ATAD3B, ATAD3A, C1orf70, SSU72, MIB2, MMP23B, CDC2L2, SLC35E2, NADK, GNB1, CALML6, TMEM52, C1orf222, KIAA1751, GABRD, PRKCZ, C1orf86, SKI, MORN1, RER1, PEX10, PLCH2, PANK4, HES5, TNFRSF14, C1orf93, MMEL1, TTC34, ACTRT2, PRDM16, ARHGEF16, MEGF6, TPRG1L, WDR8, TP73, CCDC27	hsa-mir-551a	[illegible]	[illegible]
1	23806982	24417619	1.01458333	24:42:00	p36.12-p36.11	TP0124E05	TP0125B0	8	0.610637	0.37804448288153	4	C11, C13, C17 , C22	ASAP3, E2F2, ID3, MDS2, RPL11, TCEB3, C1orf128, LYPLA2, GALE, HMGCL, FUCA1, CNR2, PNRC2, FUSIP1, MYOM3		V_63279, V_58372, V_84129, V_84130	V_58372, V_5477, V_5478, V_84129, V_63279, V_106702, V_84130, V_23358, V_97319
1	27334076	28315498	27:33:00	28:32:00	p36.11-p35.3	TP0125C06	TP0121A0	11	0.981422	0.327032743907021	4	C11, C13, C17 , C18	FAM46B, SLC9A1, WDTC1, TMEM222, SYTL1, MAP3K6, FCN3, CD164L2, GPR3, WASF2, AHDC1, FGR, IFI6, FAM76A, STX12, PPP1R8, C1orf38, RPA2, SMPDL3B, XKR8, EYA3		V_63340, V_84149, V_84150, V_84152, V_53092, V_48306	V_63340, V_48306, V_110512, V_53092, V_97325, V_84149, V_84150, V_84152
1	35377305	35698671	35:38:00	35:07:00	p34.3	TP0105A10	TP0101E0	3	0.321366	0.376507533322556	3	C4, C11, MPC960	DLGAP3, ZMYM6, ZMYM1, SFPQ		V_74443, V_84174, V_84175, V_74444, V_74445	V_74443, V_84174, V_84175, V_74444, V_74445, V_97331, V_23365
1	38415142	44418424	38:42:00	44:42:00	p34.3-p34.1	TP0125A04	TP0124E1	64	6,003,282	114,815,243,545,795	3	C4, C4LN, MPC960	SF3A3, FHL3, UTP11L, POU3F1, RRAGC, MYCBP, GJA9, RHBDL2, AKIRIN1, NDUFS5, MACF1, BMP8A, PABPC4, HEYL, NT5C1A, HPCAL4, PPIE, BMP8B, OXCT2, TRIT1, MYCL1, MFSD2, CAP1, PPT1, RLF, TMCO2, ZMPSTE24, COL9A2, SMAP2, ZNF643, ZNF642, DEM1, ZNF684, RIMS3, NFYC, , KCNQ4, CITED4, CTPS, SLFNL1, SCMH1, FOXO6, EDN2, HIVEP3, GUCA2B, GUCA2A, FOXJ3, RIMKLA, ZMYND12, PPCS, PPIH, YBX1, CLDN19, LEPRE1, C1orf50, CCDC23, ERMAP, ZNF691, SLC2A1, FAM183A, EBNA1BP2, WDR65, TIE1, MPL, CDC20, ELOVL1, MED8, C1orf84, KIAA0467, HYI, PTPRF, KDM4A, ST3GAL3, ARTN, IPO13	hsa-mir-30e, hsa-mir-30c-1	[illegible]	[illegible]
1	49072679	50582651	49:07:00	50:58:00	p33	TP0103E11	TP0112C1	10	1,509,972	0.731558965079183	3	C4, C4LN, C5	AGBL4, BEND5, ELAVL4		[illegible]	[illegible]
1	120449470	120650514	120:45:00	120.65	p12	TP0117B05	TP0117H1	4	0.201044	0.538518726074713	5	C9, C10, C21, C22, MPC960	NOTCH2		[illegible]	[illegible]
1	143510356	248876423	143:51:00	248.88	q21.1-q44	TP0113F03	TP0125B0	1081	105,366,067	149,470,120,465,415	15	C4, C4LN, C5, C8, C9, C10, C11, C13, C17 , C18, C21, C22, C24, MPC600, MPC298, MPC960	PPIAL4G, FAM72D, SRGAP2P2, PPIAL4B, NBPF9, PDE4DIP, SEC22B, NOTCH2NL, HFE2, TXNIP, POLR3GL, ANKRD34A, LIX1L, RBM8A, GNRHR2, PEX11B, ITGA10, ANKRD35, PIAS3, NUDT17, POLR3C, RNF115, CD160, PDZK1, GPR89A, GPR89C, NBPF8, NBPF8, NBPF12, PRKAB2, FMO5, CHD1L, BCL9, ACP6, GJA5, GJA8, GPR89B, NBPF11, FAM108A2, , PPIAL4A, NBPF14, NBPF10, NBPF16, FCGR1C, HIST2H3PS2, FAM72C, PPIAL4C, FAM91A2, FCGR1A, HIST2H2BF, HIST2H3D, HIST2H4A, HIST2H3C, HIST2H2AA3, HIST2H2AA4, HIST2H3A, HIST2H4B, HIST2H2BE, BOLA1, HIST2H2AC, HIST2H2AB, SV2A, SF3B4, MTMR11, OTUD7B, VPS45, PLEKHO1, ANP32E, CA14, APH1A, C1orf54, C1orf51, MRPS21, PRPF3, RPRD2, TARS2, ECM1, ADAMTSL4, C1orf138, MCL1, ENSA, GOLPH3L, HORMAD1, CTSS, CTSK, ARNT, SETDB1, LASS2, ANXA9, FAM63A, PRUNE, BNIPL, C1orf56, CDC42SE1, MLLT11, GABPB2, SEMA6C, TNFAIP8L2, LYSMD1, SCNM1, TMOD4, VPS72, PIP5K1A, PSMD4, ZNF687, PI4KB, RFX5, SELENBP1, PSMB4, POGZ, CGN, TUFT1, SNX27, TNRC4, MRPL9, OAZ3, TDRKH, LINGO4, RORC, THEM5, THEM4, S100A10, S100A11, TCHHL1, TCHH, RPTN, HRNR, FLG, FLG2, CRNN, LCE5A, , CRCT1, LCE3E, LCE3D, LCE3B, LCE3A, LCE2D, LCE2C, LCE2B, LCE2A, LCE4A, C1orf68, KPRP, LCE1E, LCE1D, LCE1C, LCE1B, LCE1A, LCE6A, SMCP, IVL, SPRR4,	hsa-mir-554, hsa-mir-190b, hsa-mir-92b, hsa-mir-555, hsa-mir-9-1, hsa-mir-765, hsa-mir-556, hsa-mir-921, hsa-mir-1255b-2, hsa-mir-557, hsa-mir-1295, hsa-mir-214, hsa-mir-199a-2, hsa-mir-488, hsa-mir-1278, hsa-mir-181b-1, hsa-mir-181a-1, hsa-mir-1231, hsa-mir-135b, hsa-mir-29c, hsa-mir-29b-2, hsa-mir-205, hsa-mir-215, hsa-mir-194-1, hsa-mir-320b-2, hsa-mir-1182, hsa-mir-1537	[illegible]	[illegible]
2	196119668	196641923	196:12:00	196.64	q32.3	TP0206B01	TP0222C0	5	0.522255	0.324358117583938	3	C9, C17 , MPC298	SLC39A10, DNAH7		[illegible]	[illegible]
2	212235402	213737112	212:24:00	213.74	q34	TP0209E07	TP0203A0	14	150,171	0.380250882858708	4	C4LN, C9, MPC298, MPC960	ERBB4	hsa-mir-548f-2	[illegible]	[illegible]
2	241415713	242721830	241:42:00	242.72	q37.3	TP0202B05	TP0222B0	17	1,306,117	0.398607750938161	7	C10, C11, C13, C17 , C18, C22, C24	ANKMY1, DUSP28, RNPEPL1, CAPN10, GPR35, AQP12B, AQP12A, KIF1A, AGXT, C2orf54, SNED1, MTERFD2, PASK, PPP1R7, ANO7, HDLBP, Sep-02, FARP2, STK25, BOK, THAP4, ATG4B, DTYMK, ING5, D2HGDH, GAL3ST2		[illegible]	[illegible]
3	9421022	10540466	9:42	10:54	p25.3	TP0317F09	TP0322A0	12	1,119,444	0.396549770186094	4	C5, C17 , C18, C22	THUMPD3, SETD5, LHFPL4, MTMR14, CPNE9, BRPF1, OGG1, CAMK1, TADA3L, TTLL3, RPUSD3, CIDEC, JAGN1, IL17RE, IL17RC, CRELD1, PRRT3, TMEM111, FANCD2, VHL, IRAK2, TATDN2, GHRL, SEC13, ATP2B2	hsa-mir-885	[illegible]	[illegible]
3	12361374	14528477	12:36	14:53	p25.2-p25.1	TP0316A01	TP0303H0	19	2,167,103	0.442559430621964	3	C5, C18, C22	PPARG, TSEN2, MKRN2, RAF1, TMEM40, CAND2, RPL32, IQSEC1, NUP210, HDAC11, FBLN2, WNT7A, TPRXL, CHCHD4, TMEM43, XPC, LSM3, SLC6A6		[illegible]	[illegible]
3	16862126	18929393	0.72638889	0.81458333	p24.3	TP0309B09	TP0319H0	23	2,067,267	0.333352404452367	3	C5, C10, C18	PLCL2, TBC1D5, SATB1		[illegible]	[illegible]
3	46562202	53308680	46:56:00	53:31:00	p21.31-p21.1	TP0307F05	TP0323D0	76	6,746,478	0.32535668994214	5	C11, C13, C22, C24, MPC960	LRRC2, LUZPP1, TDGF1, ALS2CL, TMIE, MYL3, PTH1R, CCDC12, NBEAL2, SETD2, KIF9, KLHL18, PTPN23, SCAP, C3orf75, CSPG5, SMARCC1, DHX30, MAP4, CDC25A, CAMP, ZNF589, NME6, SPINK8, FBXW12, PLXNB1, CCDC51, CCDC72, ATRIP, TREX1, SHISA5, PFKFB4, UCN2, COL7A1, UQCRC1, TMEM89, SLC26A6, CELSR3, NCKIPSD, IP6K2, PRKAR2A, SLC25A20, C3orf71, ARIH2, P4HTM, WDR6, DALRD3, NDUFAF3, IMPDH2, QRICH1, QARS, USP19, LAMB2, CCDC71, KLHDC8B, CCDC36, C3orf62, USP4, GPX1, RHOA, TCTA, AMT, NICN1, DAG1, BSN, APEH, MST1, RNF123, AMIGO3, GMPPB, IP6K1, CDH29, C3orf54, UBA7, TRAIP, CAMKV, MST1R, MON1A, RBM6, , RBM5, SEMA3F, GNAT1, GNAI2, SEMA3B, C3orf45, IFRD2, NAT6, HYAL1, HYAL2, TUSC2, RASSF1, ZMYND10, TUSC4, CYB561D2, TMEM115, CACNA2D2, C3orf18, HEMK1, CISH, MAPKAPK3, DOCK3, ARMET, RBM15B, VPRBP, RAD54L2, TEX264, GRM2, IQCF6, , IQCF3, IQCF2, IQCF5, IQCF1, RRP9, PARP3, GPR62, PCBP4, ABHD14B, ACY1, RPL29, DUSP7, C3orf74, WDR51A, ALAS1, TWF2, PPM1M, WDR82, GLYCTK, DNAH1, BAP1, PHF7, SEMA3G, TNNC1, NISCH, STAB1, NT5DC2, PBRM1, GNL3, GLT8D1, SPCS1, NEK4, ITIH1, ITIH3, ITIH4, TMEM110, SFMBT1, RFT1, PRKCD, TKT	hsa-mir-1226, hsa-mir-425, hsa-mir-191, hsa-mir-566, hsa-let-7g, hsa-mir-135a-1	[illegible]	[illegible]
3	96145806	97242413	96:15:00	97:24:00	q11.2	TP0319H08	TP0311B0	9	1,096,607	0.445679253176561	3	C4LN, C5, C9	EPHA6		[illegible]	[illegible]
3	182609690	184338903	182.61	184:34:00	q26.33-q27.1	TP0316C08	TP0310B0	16	1,729,213	0.271026423937201	3	C11, C18, C22	ATP11B, DCUN1D1, MCCC1, LAMP3, MCF2L2, B3GNT5, KLHL6, KLHL24, YEATS2, MAP6D1, PARL, ABCC5, HTR3D, HTR3C, HTR3E, EIF2B5, DVL3, AP2M1, ABCF3, VWA5B2, ALG3, ECE2, CAMK2N2, PSMD2, EIF4G1, FAM131A, CLCN2, POLR2H, THPO, CHRD, EPHB3	hsa-mir-1224	[illegible]	[illegible]
3	193646036	197791562	193.65	197.79	q29	TP0307G10	TP0319C0	44	4,145,526	0.327594935159035	5	C9, C11, C13, C18, C24	HES1, CPN2, LRRC15, GP5, ATP13A3, TMEM44, LSG1, FAM43A, C3orf21, ACAP2, PPP1R2, APOD, MUC20, MUC4, TNK2, TFRC, ZDHHC19, PCYT1A, TCTEX1D2, TM4SF19, UBXN7, RNF168, C3orf43, WDR53, FBXO45, PIGX, LRRC33, C3orf34, PAK2, SENP5, NCBP2, PIGZ, MFI2, DLG1, BDH1, KIAA0226, FYTTD1, LRCH3, IQCG, RPL35A, LMLN	hsa-mir-570, hsa-mir-922	[illegible]	[illegible]
4	369140	3962053	0:37	0.19166667	p16.3	TP0402G09	TP0410A1	48	3,592,913	186,435,397,501,789	7	C8, C9, C11, C13, C17 , C18, C22, C24	ZNF721, PIGG, PDE6B, ATP5I, MYL5, MFSD7, PCGF3, CPLX1, GAK, TMEM175, DGKQ, SLC26A1, IDUA, FGFRL1, RNF212, TMED11P, SPON2, CTBP1, C4orf42, MAEA, KIAA1530, CRIPAK, NKX1-1, FAM53A, SLBP, TMEM129, TACC3, FGFR3, LETM1, WHSC1, WHSC2, NAT8L, POLN, HAUS3, MXD4, ZFYVE28, RNF4, C4orf8, TNIP2, SH3BP2, ADD1, MFSD10, NOP14, GRK4, HTT, C4orf44, RGS12, HGFAC, DOK7, LRPAP1, ADRA2C	hsa-mir-943	[illegible]	[illegible]
4	6506548	8583657	6:51	8:58	p16.1	TP0404C04	TP0418E0	25	2,077,109	0.413680888609372	3	C11, C13, C24	MAN2B2, MRFAP1, S100P, MRFAP1L1, CNO, KIAA0232, TBC1D14, CCDC96, GRPEL1, SORCS2, PSAPL1, AFAP1, ABLIM2, SH3TC1, HTRA3, ACOX3, C4orf23, GPR78	hsa-mir-95	[illegible]	[illegible]
4	38594738	41243420	38:59:00	41:24:00	p14-p13	TP0420D02	TP0401A1	38	2,648,682	0.282332479037612	3	C11, C13, C17	KLF3, TLR10, TLR1, TLR6, FAM114A1, TMEM156, KLHL5, WDR19, RFC1, KLB, RPL9, LIAS, UGDH, C4orf34, UBE2K, PDS5A, N4BP2, RHOH, CHRNA9, RBM47, NSUN7, APBB2	hsa-mir-574	[illegible]	[illegible]
5	18263	17652178	0:02	0.75347222	p15.33-p15.1	TP0522B10	TP0521G0	243	17,633,915	0.835890447000148	7	C9, C11, C13, C17 , C18, C22, C24, MPC600	PLEKHG4B, CCDC127, SDHA, PDCD6, C5orf55, EXOC3, SLC9A3, CEP72, TPPP, ZDHHC11, ZDHHC11B, BRD9, TRIP13, NKD2, SLC12A7, SLC6A19, SLC6A18, TERT, CLPTM1L, SLC6A3, LPCAT1, MRPL36, NDUFS6, IRX4, IRX2, C5orf38, IRX1, ADAMTS16, MED10, NSUN2, SRD5A1, POLS, ADCY2, C5orf49, FASTKD3, MTRR, SEMA5A, TAS2R1, FAM173B, CCT5, CMBL, Mar-06, ROPN1L, ANKRD33B, DAP, CTNND2, DNAH5, TRIO, FAM105A, FAM105B, ANKH, FBXL7, Mar-11, ZNF622, FAM134B, MYO10, BASP1	hsa-mir-887	[illegible]	[illegible]
5	30780094	39839094	30.78	39.84	p13.3-p13.1	TP0501F07	TP0504C0	126	9,059	0.314326668140019	5	C11, C17 , C18, C22, MPC600	CDH6, RNASEN, C5orf22, PDZD2, GOLPH3, MTMR12, ZFR, SUB1, NPR3, C5orf23, TARS, ADAMTS12, RXFP3, SLC45A2, AMACR, RAI14, TTC23L, RAD1, BXDC2, DNAJC21, AGXT2, PRLR, SPEF2, IL7R, CAPSL, UGT3A1, UGT3A2, LMBRD2, SKP2, C5orf33, RANBP3L, SLC1A3, NIPBL, C5orf42, NUP155, WDR70, GDNF, EGFLAM, LIFR, OSMR, FYB, C9, DAB2	hsa-mir-579, hsa-mir-580	[illegible]	[illegible]
5	43319910	50256774	43:32:00	50:26:00	p12-q11.1	TP0526B03	TP0508F0	40	6,936,864	0.398434613764823	5	C9, C11, C13, C17 , MPC600	CCL28, C5orf28, C5orf34, PAIP1, NNT, FGF10, MRPS30, HCN1, EMB, PARP8		[illegible]	[illegible]
5	55573225	56228895	55:57:00	56:23:00	q11.2	TP0505E07	TP0527H1	10	0.65567	0.461865493528956	3	C9, C13, C17	MAP3K1, C5orf35, MIER3		[illegible]	[illegible]
5	65203593	65327823	65:02:00	65:33:00	q12.3	TP0521H11	TP0527E0	3	0.12423	0.324517149134078	3	C13, C17 , C18	ERBB2IP		[illegible]	[illegible]
5	68855238	70144801	68.86	70:14:00	q13.2	TP0527A09	TP0508H0	11	1,289,563	0.417957851693426	4	C4LN, C8, C17 , MPC298	GTF2H2B, SERF1B, SMN2, GUSBP1		[illegible]	[illegible]
5	74186633	74573937	74:19:00	74:57:00	q13.3	TP0120F09	TP0527A0	4	0.387304	0.237816807641674	3	C6, C17 , MPC600	GCNT4, ANKRD31		[illegible]	[illegible]
5	75339160	81542777	75:34:00	81:54:00	q13.3-q14.2	TP0527B02	TP0519B0	73	6,203,617	0.447545498842974	4	C9, C13, C17 , C22, MPC600	SV2C, IQGAP2, F2RL2, F2R, F2RL1, S100Z, CRHBP, AGGF1, ZBED3, PDE8B, WDR41, OTP, TBCA, AP3B1, SCAMP1, LHFPL2, ARSB, DMGDH, BHMT2, BHMT, JMY, HOMER1, PAPD4, CMYA5, MTX3, THBS4, SERINC5, SPZ1, ZFYVE16, FAM151B, ANKRD34B, DHFR, MSH3, RASGRF2, CKMT2, ZCCHC9, ACOT12, SSBP2, ATG10		[illegible]	[illegible]
5	131687191	134838807	131.69	134.84	q31.1	TP0511B01	TP0508F0	42	3,151,616	0.355697118115425	5	C10, C13, C17 , C18, C22	SLC22A5, C5orf56, IRF1, IL5, RAD50, IL13, IL4, KIF3A, CCNI2, Sep-08, ANKRD43, SHROOM1, GDF9, UQCRQ, LEAP2, AFF4, ZCCHC10, HSPA4, FSTL4, C5orf15, VDAC1, TCF7, SKP1, PPP2CA, CDKL3, UBE2B, PHF15, SAR1B, SEC24A, CAMLG, DDX46, C5orf24, TXNDC15, PCBD2, CATSPER3, PITX1, H2AFY, TIFAB, C5orf20	hsa-mir-1289-2, hsa-mir-1974	[illegible]	[illegible]
5	136600472	142272006	136:06:00	142:27:00	q31.2-q31.3	TP0505H11	TP0517G1	74	5,671,534	0.38803030379667	4	C13, C17 , C18, C22	SPOCK1, KLHL3, HNRNPA0, MYOT, PKD2L2, FAM13B, WNT8A, NME5, BRD8, KIF20A, CDC23, GFRA3, CDC25C, FAM53C, KDM3B, REEP2, EGR1, ETF1, HSPA9, CTNNA1, LRRTM2, SIL1, MATR3, , PAIP2, SLC23A1, DNAJC18, ECSCR, TMEM173, UBE2D2, CXXC5, PSD2, NRG2, PURA, C5orf53, C5orf32, PFDN1, HBEGF, SLC4A9, ANKHD1, SRA1, APBB3, SLC35A4, CD14, TMCO6, NDUFA2, WDR55, DND1, HARS, ZMAT2, PCDHA1, PCDHA2, PCDHA3, PCDHA4, PCDHA5, PCDHAC2, PCDHA7, PCDHA8, PCDHA9, PCDHB1, PCDHB2, PCDHB3, PCDHB4, PCDHB5, PCDHB6, PCDHB7, PCDHB8, PCDHB16, PCDHB10, PCDHB11, PCDHB12, PCDHB13, PCDHB14, PCDHB18, PCDHB15, SLC25A2, TAF7, PCDHGA1, PCDHGC4, DIAPH1, HDAC3, RELL2, FCHSD1, ARAP3, PCDH1, KIAA0141, PCDH12, RNF14, GNPDA1, NDFIP1, SPRY4, FGF1, ARHGAP26	hsa-mir-874	[illegible]	[illegible]
5	148593233	150093627	148:59:00	150:09:00	q32-q33.1	TP0505F12	TP0518D0	14	1,500,394	0.337164282129486	5	C11, C13, C17 , C22, C24	ABLIM3, AFAP1L1, GRPEL2, PCYOX1L, IL17B, PPARGC1B, PDE6A, SLC26A2, TIGD6, HMGXB3, CSF1R, PDGFRB, CDX1, SLC6A7, CAMK2A, ARSI, TCOF1, CD74, RPS14, NDST1, SYNPO, MYOZ3, RBM22, DCTN4	hsa-mir-143, hsa-mir-145, hsa-mir-378	[illegible]	[illegible]
5	160495477	162350004	160:05:00	162:35:00	q34	TP0516H12	TP0509D0	18	1,854,527	0.32189881750552	3	C4LN, C17 , MPC600	GABRB2, GABRA6, GLRXP3, GABRA1, GABRG2		[illegible]	[illegible]
5	170410834	180676573	170:41:00	180.68	q35.1-q35.3	TP0527C05	TP0507D1	151	10,265,739	0.412359211840646	8	C4LN, C9, C11, C13, C17 , C18, C22, C24, MPC600	RANBP17, TLX3, NPM1, FGF18, C5orf50, FBXW11, STK10, EFCAB9, UBTD2, SH3PXD2B, NEURL1B, DUSP1, ERGIC1, RPL26L1, ATP6V0E1, C5orf41, BNIP1, NKX2-5, STC2, BOD1, CPEB4, C5orf47, MSX2, DRD1, SFXN1, HRH2, CPLX2, THOC3, FAM153B, C5orf25, KIAA1191, ARL10, NOP16, HIGD2A, CLTB, FAF2, RNF44, PCDH24, GPRIN1, SNCB, EIF4E1B, TSPAN17, UNC5A, HK3, UIMC1, ZNF346, FGFR4, NSD1, RAB24, PRELID1, MXD3, LMAN2, RGS14, SLC34A1, PFN3, F12, GRK6, PRR7, DBN1, PDLIM7, DOK3, DDX41, TMED9, B4GALT7, FAM153A, PROP1, FAM153C, RMND5B, NHP2, HNRNPAB, AGXT2L2, COL23A1, CLK4, ZNF354A, ZNF354B, ZFP2, ZNF454, GRM6, ZNF354C, ADAMTS2, RUFY1, HNRNPH1, CBY3, CANX, MAML1, LTC4S, MGAT4B, SQSTM1, C5orf45, TBC1D9B, RNF130, RASGEF1C, RPS8P7, MAPK9, GFPT2, CNOT6, SCGB3A1, FLT4, OR2Y1, MGAT1, ZFP62, BTNL8, , BTNL3, BTNL9, OR2V1, OR2V2, TRIM7, TRIM41, GNB2L1	hsa-mir-1271, hsa-mir-1229, hsa-mir-340	[illegible]	[illegible]
6	129242	37853446	0:13	37.85	p25.3-p21.2	TP0619G09	TP0616C0	451	37,724,204	0.746312499222747	8	C4, C4LN, C8, C10, C11, C13, C17 , C18, C24, MPC298, MPC960	DUSP22, IRF4, EXOC2, HUS1B, FOXQ1, FOXF2, FOXC1, GMDS, C6orf195, MYLK4, WRNIP1, SERPINB1, SERPINB9, SERPINB6, NQO2, RIPK1, BPHL, TUBB2A, TUBB2B, PSMG4, SLC22A23, C6orf145, FAM50B, C6orf50, PRPF4B, C6orf146, C6orf201, PECI, CDYL, RPP40, PPP1R3G, FARS2, C6orf202, NRN1, F13A1, LY86, RREB1, SSR1, CAGE1, RIOK1, DSP, SNRNP48, BMP6, TXNDC5, EEF1E1, SLC35B3, OFCC1, TFAP2A, C6orf218, GCNT2, GCNT6, C6orf52, PAK1IP1, TMEM14C, TMEM14B, MAK, GCM2, SYCP2L, ELOVL2, NEDD9, TMEM170B, C6orf105, HIVEP1, EDN1, PHACTR1, TBC1D7, GFOD1, C6orf114, SIRT5, NOL7, RANBP9, CCDC90A, CD83, JARID2, DTNBP1, MYLIP, GMPR, ATXN1, RBM24, CAP2, FAM8A1, NUP153, KIF13A, NHLRC1, TPMT, AOF1, DEK, RNF144B, ID4, MBOAT1, E2F3, CDKAL1, SOX4, PRL, HDGFL1, NRSN1, DCDC2, KAAG1, MRS2, GPLD1, ALDH5A1, KIAA0319, TTRAP, ACOT13, C6orf62, GMNN, , FAM65B, CMAH, LRRC16A, SCGN, HIST1H2AA, HIST1H2BA, SLC17A4, SLC17A1, SLC17A3, SLC17A2, TRIM38, HIST1H1A, HIST1H3A, HIST1H4A, HIST1H4B, HIST1H3B, HIST1H2AB, HIST1H2BB, HIST1H3C, HIST1H1C, HFE, HIST1H4C, HIST1H1T, HIST1H2BC, HIST1H2AC, HIST1H1E, HIST1H2BD, HIST1H2BE, HIST1H4D, HIST1H3D, HIST1H2AD, HIST1H2BF, HIST1H4E, HIST1H2BG, HIST1H2AE, HIST1H3E, HIST1H1D, HIST1H4F, HIST1H4G, HIST1H3F, HIST1H2BH,	hsa-mir-548a-1, hsa-mir-877, hsa-mir-1236, hsa-mir-219-1, hsa-mir-1275	[illegible]	[illegible]
6	41249867	56418599	41:25:00	56:42:00	p21.1-p12.1	TP0608C08	TP0606C0	176	15,168,732	0.82671583714761	6	C4, C4LN, C5, C8, C11, C13, C22, C24	TREM1, NCR2, FOXP4, MDFI, TFEB, PGC, FRS3, PRICKLE4, TOMM6, USP49, MED20, BYSL, CCND3, TAF8, C6orf132, GUCA1A, GUCA1B, MRPS10, TRERF1, UBR2, PRPH2, TBCC, KIAA0240, RPL7L1, C6orf226, PTCRA, CNPY3, GNMT, PEX6, PPP2R5D, MEA1, KLHDC3, C6orf153, CUL7, MRPL2, KLC4, PTK7, SRF, CUL9, C6orf108, TTBK1, SLC22A7, CRIP3, ZNF318, ABCC10, DLK2, TJAP1, C6orf154, YIPF3, POLR1C, XPO5, POLH, GTPBP2, MAD2L1BP, RSPH9, MRPS18A, VEGFA, MRPL14, TMEM63B, CAPN11, SLC29A1, HSP90AB1, SLC35B2, NFKBIE, TMEM151B, TCTE1, AARS2, CDC5L, SUPT3H, RUNX2, CLIC5, ENPP4, ENPP5, RCAN2, CYP39A1, SLC25A27, TDRD6, PLA2G7, MEP1A, GPR116, GPR110, TNFRSF21, CD2AP, GPR111, GPR115, OPN5, C6orf138, MUT, CENPQ, C6orf140, RHAG, CRISP2, CRISP3, PGK2, CRISP1, DEFB133, DEFB114, DEFB113, DEFB111, DEFB112, TFAP2D, TFAP2B, RPS17P5, PKHD1, IL17A, IL17F, MCM3, PAQR8, EFHC1, TRAM2, TMEM14A, GSTA2, GSTA1, GSTA5, GSTA3, GSTA4, ICK, FBXO9, GCM1, ELOVL5, GCLC, KLHL31, LRRC1, C6orf142, TINAG, FAM83B, HCRTR2, GFRAL, HMGCLL1, BMP5, COL21A1, DST	hsa-mir-586, hsa-mir-206, hsa-mir-133b	[illegible]	[illegible]
6	65350799	68239058	65:35:00	68:24:00	q12	TP0615D12	TP0621B0	29	2,888,259	0.343763583751471	3	C5, C8, MPC600	EYS		[illegible]	[illegible]
6	122930895	165669953	122.93	165.67	q22.31-q27	TP0610G11	TP0601E0	482	42,739,058	122,421,648,427,578	5	C4, C4LN, C8, C11, C21, C24	PKIB, FABP7, SMPDL3A, RLBP1L2, TRDN, NKAIN2, RNF217, TPD52L1, HDDC2, HEY2, NCOA7, HINT3, TRMT11, C6orf173, RSPO3, RNF146, ECHDC1, C6orf174, C6orf58, TSEPA, PTPRK, LAMA2, ARHGAP18, C6orf191, L3MBTL3, SAMD3, TMEM200A, EPB41L2, AKAP7, ARG1, MED23, ENPP3, OR2A4, CTAGE4, ENPP1, CTGF, MOXD1, STX7, TAAR9, TAAR8, TAAR6, TAAR5, TAAR2, TAAR1, VNN1, VNN3, VNN2, C6orf192, RPS12, EYA4, TCF21, TBPL1, SLC2A12, SGK1, ALDH8A1, HBS1L, MYB, AHI1, PDE7B, FAM54A, BCLAF1, MAP7, MAP3K5, PEX7, SLC35D3, IL20RA, IL22RA2, IFNGR1, OLIG3, TNFAIP3, PERP, KIAA1244, PBOV1, HEBP2, NHSL1, CCDC28A, ECT2L, REPS1, C6orf115, HECA, TXLNB, CITED2, NMBR, VTA1, GPR126, HIVEP2, AIG1, ADAT2, PEX3, FUCA2, PHACTR2, LTV1, FAM164B, PLAGL1, SF3B5, STX11, UTRN, EPM2A, FBXO30, SHPRH, GRM1, RAB32, C6orf103, STXBP5, SAMD5, SASH1, UST, MAP3K7IP2, SUMO4, ZC3H12D, PPIL4, C6orf72, KATNA1, LATS1, NUP43, PCMT1, LRP11, RAET1E, RAET1G, ULBP2, ULBP1, RAET1L, ULBP3, PPP1R14C, IYD, PLEKHG1, MTHFD1L, AKAP12, ZBTB2, RMND1, C6orf211, C6orf97, ESR1, SYNE1, MYCT1, VIP, FBXO5, MTRF1L, RGS17, OPRM1, IPCEF1, MAGI1, RBM16, TIAM2, TFB1M, CLDN20, NOX3, ARID1B, C6orf35, ZDHHC14, SNX9, SYNJ2, SERAC1, GTF2H5, TULP4, TMEM181, DYNLT1, SYTL3, EZR, C6orf99,	hsa-mir-588, hsa-mir-548a-2, hsa-mir-1202	[illegible]	[illegible]
6	165919402	171008924	165.92	171:01:00	q27	TP0615C11	TP0603G0	54	5,089,522	126,976,044,078,506	6	C4, C4LN, C8, C11, C22, C24, MPC298	PDE10A, T, PRR18, SFT2D1, BRP44L, RPS6KA2, RNASET2, FGFR1OP, CCR6, GPR31, TCP10L2, UNC93A, TTLL2, TCP10, C6orf123, C6orf124, MLLT4, C6orf54, KIF25, FRMD1, DACT2, SMOC2,THBS2, WDR27, C6orf120, PHF10, TCTE3, C6orf70, DLL1, FAM120B, PSMB1, TBP, PDCD2	hsa-mir-1913	[illegible]	[illegible]
7	758897	43545884	0.05277778	43:55:00	p22.3-p13	TP0701D10	TP0711A0	454	42,786,987	0.929383531810751	9	C4, C4LN, C5, C9, C10, C11, C13, C17 , C18, C22, C24, MPC600, MPC298, MPC960	PRKAR1B, HEATR2, UNC84A, ADAP1, CYP2W1, C7orf50, GPR146, GPER, ZFAND2A, UNCX, MICALL2, INTS1, MAFK, TMEM184A, PSMG3, MAD1L1, FTSJ2, NUDT1, SNX8, EIF3B, CHST12, LFNG, C7orf27, IQCE, TTYH3, AMZ1, GNA12, CARD11, SDK1, FOXK1, RADIL, PAPOLB, MMD2, RNF216L, RBAK, WIPI2, SLC29A4, TNRC18, FBXL18, ACTB, FSCN1, RNF216, OCM, C7orf28A, RSPH10B, PMS2, AIMP2, EIF2AK1, ANKRD61, USP42, CYTH3, C7orf70, RAC1, DAGLB, KDELR2, GRID2IP, ZDHHC4, C7orf26, ZNF12, RSPH10B2, C7orf28B, C1GALT1, COL28A1, MIOS, RPA3, GLCCI1, ICA1, NXPH1, NDUFA4, PHF14, THSD7A, TMEM106B, VWDE, SCIN, ARL4A, ETV1, DGKB, TMEM195, MEOX2, SOSTDC1, ANKMY2, BZW2, TSPAN13, AGR2, AGR3, AHR, SNX13, PRPS1L1, HDAC9, TWIST1, FERD3L, TWISTNB, TMEM196, MACC1, ITGB8, ABCB5, SP8, SP4, DNAH11, CDCA7L, RAPGEF5, IL6, TOMM7, FAM126A, KLHL7, NUPL2, GPNMB, C7orf30, IGF2BP3, TRA2A, CCDC126, C7orf46, STK31, NPY, MPP6, DFNA5, OSBPL3, CYCS, C7orf31, NPVF, NFE2L3, HNRNPA2B1, CBX3, SNX10, KIAA0087, SKAP2, HOXA1, HOXA2, HOXA3, HOXA4, HOXA5, HOXA6, HOXA7, HOXA9, HOXA10, HOXA11, HOXA13, EVX1, HIBADH, TAX1BP1, JAZF1, CREB5, CPVL, CHN2, PRR15, WIPF3, SCRN1, FKBP14, PLEKHA8, C7orf41, ZNRF2, NOD1, GGCT, GARS, CRHR2, INMT, GHRHR, ADCYAP1R1, NEUROD6,	hsa-mir-339, hsa-mir-589, hsa-mir-1302-6, hsa-mir-1183, hsa-mir-148a, hsa-mir-196b, hsa-mir-550-1, hsa-mir-550-2, hsa-mir-548n, hsa-mir-1200	[illegible]	[illegible]
7	44048777	45152091	44:05:00	45:15:00	p13	TP0710C09	TP0719E0	16	1,103,314	0.276443942487491	4	C10, C11, C22, MPC298	POLR2J4, SPDYE1, DBNL, PGAM2, POLM, AEBP1, POLD2, MYL7, GCK, YKT6, CAMK2B, NUDCD3,NPC1L1, DDX56, TMED4, OGDH, ZMIZ2, PPIA, H2AFV, PURB, MYO1G, C7orf40, CCM2, NACAD, TBRG4		[illegible]	[illegible]
7	45921643	49563171	45.92	49:56:00	p12.3-p12.2	TP0703C08	TP0716H0	42	3,641,528	0.976837723969196	3	C10, MPC600, MPC298	IGFBP1, IGFBP3, TNS3, C7orf65, HUS1, PKD1L1, C7orf69, SUNC1, C7orf57, UPP1, ABCA13, CDC14C		[illegible]	[illegible]
7	52875271	54199428	52.88	54:02:00	p12.1-p11.2	TP0708G06	TP0713C1	11	1,324,157	0.295371839092899	3	C10, MPC600, MPC298	POM121L12		[illegible]	[illegible]
7	54640605	55702142	54.64	55:07:00	p11.2	TP0707H12	TP0705A1	8	1,061,537	0.473430280590767	3	C10, C18, MPC600	SEC61G, EGFR, LANCL2		[illegible]	[illegible]
7	57191874	57557880	57:19:00	57:56:00	p11.2-p11.1	TP0716F11	TP0707C0	7	0.366006	0.518123748573589	3	C4, C4LN, C18	ZNF479, ZNF716		[illegible]	[illegible]
7	64571369	65190324	64:57:00	65:19:00	q11.21	TP0717G04	TP0701B1	26	0.618955	0.361636539973445	3	C9, MPC600, MPC960	ZNF92		[illegible]	[illegible]
7	72399774	76124370	72:04:00	76:12:00	q11.23	TP0713E12	TP0706D1	51	3,724,596	0.343451032167656	6	C10, C11, C13, C18, C22, C24	POM121, NSUN5C, TRIM74, STAG3L3, NSUN5, TRIM50, FKBP6, FZD9, BAZ1B, BCL7B, TBL2, MLXIPL, VPS37D, DNAJC30, WBSCR22, STX1A, ABHD11, CLDN3, CLDN4, WBSCR27, WBSCR28, ELN, LIMK1, EIF4H, LAT2, RFC2, CLIP2, GTF2IRD1, GTF2I, STAG3L2, NCF1, GTF2IRD2, PMS2L5, WBSCR16, GTF2IRD2B, NCF1C, GATSL1, STAG3L1, TRIM73, NSUN5B, POM121C, PMS2L3, HIP1, CCL26, CCL24, RHBDD2, POR, TMEM120A, STYXL1, MDH2, HSPB1, YWHAG, SRCRB4D, ZP3, UPK3B	hsa-mir-590	[illegible]	[illegible]
7	76287792	95471519	76:29:00	95:47:00	q11.23-q21.3	TP0707B02	TP0702E0	217	19,183,727	0.429661463677706	4	C5, C9, C10, C18, MPC600	PMS2L11, CCDC146, FGL2, PION, PTPN12, RSBN1L, TMEM60, PHTF2, MAGI2, GNAI1, CD36, GNAT3, SEMA3C, HGF, CACNA2D1, PCLO, SEMA3E, SEMA3A, SEMA3D, GRM3, KIAA1324L, DMTF1, C7orf23, CROT, ABCB4, ABCB1, RUNDC3B, SLC25A40, DBF4, ADAM22, SRI, STEAP4, ZNF804B, C7orf62, STEAP1, STEAP2, C7orf63, GTPBP10, CLDN12, PFTK1, FZD1, MTERF, AKAP9, CYP51A1, KRIT1, ANKIB1, GATAD1, PEX1, C7orf64, FAM133B, CDK6, SAMD9, SAMD9L, HEPACAM2, CCDC132, CALCR, GNGT1, TFPI2, GNG11, BET1, COL1A2, CASD1, SGCE, PPP1R9A, PON1, PON3, PON2, ASB4, PDK4, DYNC1I1	hsa-mir-1285-1, hsa-mir-653, hsa-mir-489	[illegible]	[illegible]
7	97741101	102185532	97.74	102:19:00	q21.3-q22.1	TP0704F11	TP0717H1	49	4,444,431	0.465121938252889	4	C10, C11, C13, C22	LMTK2, BHLHA15, TECPR1, BRI3, BAIAP2L1, NPTX2, TMEM130, TRRAP, SMURF1, KPNA7, ARPC1B, PDAP1, BUD31, PTCD1, CPSF4, ZNF789, ZNF394, ZKSCAN5, C7orf38, ZNF655, ZNF498, CYP3A5, CYP3A7, CYP3A5P1, CYP3A4, CYP3A43, TRIM4, GJC3, AZGP1, AZGP1P1, ZKSCAN1, ZSCAN21, ZNF3, COPS6, MCM7, AP4M1, TAF6, CNPY4, MBLAC1, C7orf59, C7orf43, GAL3ST4, GPC2, STAG3, GATS, PVRIG, PMS2L1, PILRB, PILRA, ZCWPW1, MEPCE, C7orf47, C7orf61, TSC22D4, C7orf51, AGFG2, LRCH4, FBXO24, PCOLCE, MOSPD3, TFR2, ACTL6B, GNB2, GIGYF1, POP7, EPO, ZAN, EPHB4, SLC12A9, TRIP6, SRRT, UFSP1, ACHE, MUC3B, MUC3A, MUC12, MUC17, TRIM56, SERPINE1, AP1S1, VGF, C7orf52, MOGAT3, PLOD3, ZNHIT1, CLDN15, FIS1, RABL5, EMID2, MYL10, CUX1, SH2B2, PRKRIP1, ORAI2, ALKBH4, LRWD1, POLR2J, RASA4B, POLR2J3	hsa-mir-25, hsa-mir-93, hsa-mir-106b, hsa-mir-548o	[illegible]	[illegible]
7	106489894	108246283	106:49:00	108:25:00	q22.3-q31.1	TP0708H11	TP0703D0	20	1,756,389	0.294168427501831	3	C10, C13, MPC600	PIK3CG, PRKAR2B, HBP1, COG5, GPR22, DUS4L, BCAP29, SLC26A4, CBLL1, SLC26A3, DLD, LAMB1, LAMB4, NRCAM, PNPLA8, THAP5, DNAJB9		[illegible]	[illegible]
7	119465059	126034819	119:47:00	126:03:00	q31.31-q31.33	TP0709B08	TP0706D0	62	656,976	0.499544862531796	3	C10, C18, MPC600	KCND2, TSPAN12, ING3, C7orf58, WNT16, FAM3C, PTPRZ1, AASS, FEZF1, CADPS2, RNF133, RNF148, TAS2R16, SLC13A1, IQUB, NDUFA5, ASB15, LMOD2, WASL, HYAL4, SPAM1, GPR37,POT1		[illegible]	[illegible]
7	127612119	130292156	127.61	130:29:00	q32.1-q32.2	TP0714B07	TP0717H0	30	2,680,037	0.413501276338488	5	C10, C11, C13, C18, C22	SND1, C7orf54, LRRC4, LEP, RBM28, IMPDH1, C7orf68, METTL2B, FAM71F2, FAM71F1, CALU, OPN1SW, CCDC136, FLNC, ATP6V1F, KCP, IRF5, TNPO3, TSPAN33, SMO, AHCYL2, FAM40B, NRF1, UBE2H, ZC3HC1, KLHDC10, TMEM209, C7orf45, CPA2, CPA4, CPA5, CPA1, TSGA14, MEST	hsa-mir-593, hsa-mir-129-1, hsa-mir-182, hsa-mir-96, hsa-mir-183, hsa-mir-335	[illegible]	[illegible]
7	137327576	140778447	137:33:00	140.78	q33-q34	TP0715H01	TP0707G0	41	3,450,871	0.335434772594161	4	C10, C13, C18, C22	DGKI, CREB3L2, AKR1D1, TRIM24, SVOPL, ATP6V0A4, TMEM213, KIAA1549, ZC3HAV1L, ZC3HAV1, TTC26, UBN2, LUC7L2, C7orf55, KLRG2, CLEC2L, HIPK2, TBXAS1, PARP12, JHDM1D, SLC37A3, RAB19, MKRN1, DENND2A, ADCK2, NDUFB2, BRAF, MRPS33		[illegible]	[illegible]
7	144215478	147585702	144:22:00	147:59:00	q35	TP0706C03	TP0705D0	34	3,370,224	0.454793015056337	4	C5, C6, C10, MPC600	TPK1, CNTNAP2	hsa-mir-548f-4	[illegible]	[illegible]
7	148491471	151956442	148:49:00	151.96	q36.1	TP0719F08	TP0703D1	36	3,464,971	0.371584360633918	4	C9, C10, C13, C22	CUL1, EZH2, PDIA4, ZNF786, ZNF398, ZNF425, ZNF282, ZNF212, ZNF783, ZNF777, ZNF746, ZNF767, KRBA1, ZNF467, SSPO, ZNF862, ATP6V0E2, LRRC61, C7orf29, RARRES2, REPIN1, ZNF775, GIMAP8, GIMAP7, GIMAP4, GIMAP6, GIMAP2, GIMAP1, GIMAP5, TMEM176B, TMEM176A, ABP1, KCNH2, NOS3, ATG9B, ABCB8, ACCN3, CDK5, SLC4A2, FASTK, TMUB1, AGAP3, GBX1, ASB10, ABCF2, SMARCD3, NUB1, WDR86, CRYGN, RHEB, PRKAG2, GALNTL5, GALNT11, MLL3	hsa-mir-1975, hsa-mir-671	[illegible]	[illegible]

7	154730409	159124355	154.73	159:12:00	q36.2-q36.3	TP0713H10	TP0720A1	56	4,393,946	0.856166871921664	6	C8, C9, C10, C11, C13, C22	[illegible]	hsa-mir-153-2, hsa-mir-595	[illegible]	[illegible]
8	21518123	23175482	21:52	23:18	p21.3	TP0816G06	TP0803A0	24	1,657,359	0.507270616306978	3	C11, C17 , C22	[illegible]	hsa-mir-320a	[illegible]	[illegible]
8	27269920	146236289	27:27:00	146:24:00	p21.2-q24.3	TP0811E04	TP0816E0	1152	118,966,369	175,338,821,929,454	15	C4, C4LN, C5, C6, C8, C9, C10, C11, C13, C17 , C18, C21, C22, C24, MPC600, MPC298, MPC960	[illegible]	hsa-mir-486, hsa-mir-124-2, hsa-mir-599, hsa-mir-875, hsa-mir-1273, hsa-mir-548a-3, hsa-mir-2053, hsa-mir-548d-1, hsa-mir-1204, hsa-mir-1205, hsa-mir-1207, hsa-mir-1208, hsa-mir-30b, hsa-mir-30d, hsa-mir-151, hsa-mir-937, hsa-mir-661, hsa-mir-939, hsa-mir-1234	[illegible]	[illegible]
9	8977928	10212882	0.40138889	10:21	p24.1-p23	TP0912E05	TP0904G0	12	1,234,954	0.319104903838161	3	C4, C4LN, C9	PTPRD		[illegible]	[illegible]
9	74930478	75210226	74.93	75:21:00	q21.13	TP0911E01	TP0912C0	5	0.279748	0.327847313498764	3	C18, C21, C22	ZFAND5, TMC1		[illegible]	[illegible]
9	88381980	88836459	88:38:00	88.84	q21.33	TP0904B08	TP0914A0	4	0.454479	0.224268242358926	3	C5, C6, C18	MAK10, GOLM1, C9orf153		[illegible]	[illegible]
9	126603702	129923235	126:06:00	129.92	q33.3	TP0902A02	TP0907F0	34	3,319,533	0.652757317009808	5	C11, C13, C17 , C18, C21	[illegible]	hsa-mir-181a-2, hsa-mir-181b-2	[illegible]	[illegible]
9	130097491	134813585	130:01:00	134.81	q33.3-q34.13	TP0911B08	TP0905B1	53	4,716,094	0.43378432672301	7	C10, C11, C13, C17 , C18, C22, C24	[illegible]	hsa-mir-199b, hsa-mir-219-2	[illegible]	[illegible]
9	135088447	140969997	135:09:00	140.97	q34.13-q34.3	TP0906G03	TP0914E0	60	588,155	0.585652250846874	6	C11, C13, C17 , C18, C22, C24	[illegible]	hsa-mir-126, hsa-mir-602	[illegible]	[illegible]
10	8689042	9301735	0.38125	9:03	p14	TP1008H02	TP1001B1	7	0.612693	0.490117297793961	3	C5, C9, C10			[illegible]	[illegible]
10	43500191	44199915	43:05:00	44:02:00	q11.21	TP1006B06	TP1012E1	12	0.699724	0.232606312949297	4	C11, C13, C17 , C22	[illegible]		[illegible]	[illegible]
10	133922699	135361373	133.92	135:36:00	q26.3	TP1003F03	TP1009C1	24	1,438,674	0.427762704780059	7	C8, C10, C11, C13, C17 , C22, C24	[illegible]	hsa-mir-202	[illegible]	[illegible]
11	61992	2018246	0:06	2:02	p15.5	TP0714F02	TP1114F0	19	1,956,254	0.880891995178666	7	C8, C10, C11, C13, C17 , C22, C24	[illegible]	hsa-mir-210, hsa-mir-675	[illegible]	[illegible]
11	50540424	56134529	50:54:00	56:13:00	p11.12-q11	TP1112C06	TP1106A1	21	5,594,105	0.548152154428076	4	C4LN, C8, MPC298, MPC960	[illegible]		[illegible]	[illegible]
11	57057410	57596517	57:06:00	57:06:00	q11-q12.1	TP1115B10	TP1109H0	7	0.539107	0.215034241646757	4	C11, C13, C22, C24	[illegible]	hsa-mir-130a	[illegible]	[illegible]
11	60817912	70917945	60.82	70.92	q12.2-q13.4	TP1106H11	TP1114E0	116	10,100,033	0.670209028771735	7	C5, C9, C10, C11, C13, C17 , C18, C22, C24, MPC600	[illegible]	hsa-mir-611, hsa-mir-1908, hsa-mir-1237, hsa-mir-192, hsa-mir-194-2, hsa-mir-612, hsa-mir-548k	[illegible]	[illegible]
11	71885419	76969903	71.89	76.97	q13.4-q13.5	TP1112H02	TP1107B1	51	5,084,484	0.950070532443718	4	C9, C18, C22, MPC600	[illegible]	hsa-mir-139, hsa-mir-326	[illegible]	[illegible]
12	147333	3642457	0:15	0.16944444	p13.33-p13.32	TP1215A06	TP1214D1	37	3,495,124	0.334144553187578	5	C4, C4LN, C17 , C18, C22	[illegible]		[illegible]	[illegible]
12	5878035	8017322	0.26944444	8:02	p13.31	TP1213A12	TP1215B0	26	2,139,287	0.365630780585892	8	C4, C4LN, C10, C11, C13, C17 , C18, C22, C24	[illegible]	hsa-mir-200c, hsa-mir-141	[illegible]	[illegible]
12	47932695	55110124	47.93	55:11:00	q13.11-q13.2	TP1212A04	TP1216C0	93	7,177,429	0.679188261423635	6	C4, C4LN, C9, C17 , C18, C22, C24	[illegible]	hsa-mir-1293, hsa-mir-196a-2, hsa-mir-615, hsa-mir-148b	[illegible]	[illegible]
12	55437659	55884797	55:44:00	55.88	q13.2	TP1204G12	TP0212H0	5	0.447138	0.221596772377812	3	C4, C18, MPC298	[illegible]		[illegible]	[illegible]
12	55941437	75490536	55.94	75:49:00	q13.2-q21.1	TP1209B10	TP1207G0	253	19,549,099	0.470007177742908	6	C4, C4LN, C5, C11, C17 , C18, C21, C22, C24, MPC960	[illegible]	hsa-mir-1228, hsa-mir-616, hsa-mir-26a-2, hsa-let-7i, hsa-mir-548c	[illegible]	[illegible]
12	78954574	79547349	78.95	79:55:00	q21.2	TP1213G08	TP1205G0	4	0.592775	0.287599462074705	3	C4, C4LN, C21	[illegible]		[illegible]	[illegible]
12	79755474	80220795	79.76	80:22:00	q21.2	TP0120C02	TP1201G1	4	0.465321	0.378547892492239	3	C4, C4LN, C11	[illegible]	hsa-mir-1252	[illegible]	[illegible]
12	81005697	90855725	81:01:00	90.86	q21.31-q21.33	TP1208G07	TP1211B0	96	9,850,028	0.419201177145331	3	C4, C4LN, MPC298	[illegible]	hsa-mir-617, hsa-mir-618	[illegible]	[illegible]
12	91419146	91781428	91:42:00	91.78	q21.33	TP1202C02	TP1208B1	5	0.362282	0.287610432239635	3	C4, C4LN, MPC960	[illegible]		[illegible]	[illegible]
12	99266655	100457466	99:27:00	100:46:00	q23.1	TP1212D03	TP1213C0	14	1,190,811	0.380388205808538	3	C4, C4LN, C9	[illegible]		[illegible]	[illegible]
12	109126915	114389563	109:13:00	114:39:00	q24.11-q24.21	TP1204C07	TP1204F0	54	5,262,648	0.459503054811207	4	C4, C11, C17 , C22	[illegible]	hsa-mir-619, hsa-mir-1302-1	[illegible]	[illegible]
12	116160956	117185485	116:16:00	117:19:00	q24.21-q24.22	TP1202E12	TP1209H0	15	1,024,529	0.429826535950693	5	C4, C9, C10, C17 , C22	[illegible]	hsa-mir-620	[illegible]	[illegible]
12	120273279	125929224	120:27:00	125.93	q24.23-q24.32	TP1207A11	TP1213G0	69	5,655,945	0.493913431012256	7	C4, C4LN, C9, C11, C13, C17 , C22, C24	[illegible]		[illegible]	[illegible]
12	130305483	133778461	130:31:00	133.78	q24.33	TP1204H01	TP1216B0	33	3,472,978	0.396119893136318	6	C4, C4LN, C11, C13, C17 , C22	[illegible]		[illegible]	[illegible]
13	111101235	111485176	111:01:00	111:49:00	q34	TP1307G05	TP1302G0	3	0.383941	0.235034077539352	3	C13, C17 , C22	[illegible]		[illegible]	[illegible]
13	113326849	115074090	113:33:00	115:07:00	q34	TP1307H03	TP1303F0	19	1,747,241	0.972921219235886	8	C9, C10, C11, C13, C17 , C18, C22, C24	[illegible]		[illegible]	[illegible]
14	49370874	51944718	49:37:00	51.94	q21.3-q22.1	TP1410D09	TP1403A1	27	2,573,844	0.257065716923351	3	C11, C21, C24	[illegible]		[illegible]	[illegible]
14	63767854	66256713	63.77	66:26:00	q23.2-q23.3	TP1407D09	TP1409B1	28	2,488,859	0.35986169059068	4	C11, C13, C22, C24	[illegible]	hsa-mir-548h-1, hsa-mir-625	[illegible]	[illegible]
14	77066984	78602306	77:07:00	78:06:00	q24.3	TP1402A03	TP1401E0	15	1,535,322	0.31670357188306	4	C11, C13, C22, C24	[illegible]	hsa-mir-1260	[illegible]	[illegible]
14	90153033	97367766	90:15:00	97:37:00	q32.11-q32.2	TP1405F06	TP1407G1	67	7,214,733	0.354651400672771	4	C11, C18, C22, C24	[illegible]		[illegible]	[illegible]
14	99911057	100807862	99.91	100.81	q32.2	TP1406H01	TP1406C0	9	0.896805	0.562965685313456	4	C11, C13, C18, C24	[illegible]	hsa-mir-342, hsa-mir-345	[illegible]	[illegible]
14	102469070	106025562	102:47:00	106:03:00	q32.31-q32.33	TP1410D02	TP0002C0	40	3,556,492	0.479346033013848	6	C11, C13, C17 , C18, C22, C24	[illegible]	hsa-mir-203	[illegible]	[illegible]
15	20828305	22532828	0.89097222	22:53	q11.2	TP1510C04	TP1508F0	13	1,704,523	0.676162263793823	5	C4, C4LN, C9, MPC600, MPC960	[illegible]	hsa-mir-1268	[illegible]	[illegible]
15	63333498	64578831	63:33:00	64:58:00	q22.2-q22.31	TP1501G02	TP1505G0	11	1,245,333	0.320151022313398	3	C9, C17 , C18	[illegible]	hsa-mir-422a	[illegible]	[illegible]
15	69375862	72507429	69:38:00	72:51:00	q23	TP1504G07	TP1504H0	33	3,131,567	0.345575088585933	3	C9, C17 , C18	[illegible]	hsa-mir-629	[illegible]	[illegible]
15	89380675	91640749	89:38:00	91.64	q26.1	TP1510A01	TP1501A0	22	2,260,074	0.364104684186106	5	C11, C13, C17 , C22, C24	[illegible]	hsa-mir-9-3	[illegible]	[illegible]
15	93286541	93765257	93:29:00	93.77	q26.1	TP1506G07	TP1502C0	5	0.478716	0.300150845121623	3	C11, C17 , C22	[illegible]		[illegible]	[illegible]
15	101384065	101878279	101:38:00	101.88	q26.3	TP1502B07	TP1508B0	10	0.494214	0.322364899075056	3	C11, C17 , C22	[illegible]		[illegible]	[illegible]
16	61002	32070788	0:06	32:07:00	p13.3-p11.2	TP1602F03	TP1608D0	335	32,009,786	0.604346472780922	10	C6, C8, C10, C11, C13, C17 , C18, C22, C24, MPC960	[illegible]	hsa-mir-662, hsa-mir-1225, hsa-mir-940, hsa-mir-548h-2, hsa-mir-193b, hsa-mir-365-1, hsa-mir-1972, hsa-mir-484	[illegible]	[illegible]
16	66333595	69532599	66:33:00	69:53:00	q21-q22.1	TP1609G09	TP1604H0	32	3,199,004	0.281469788910422	3	C11, C18, C24	[illegible]	hsa-mir-328	[illegible]	[illegible]
16	85064749	85915715	85:06:00	85.92	q24.1	TP1604F03	TP1605H0	11	0.850966	0.346247575886556	4	C9, C11, C18, C24	[illegible]	hsa-mir-1910	[illegible]	[illegible]
16	87127576	90044587	87:13:00	90:04:00	q24.2-q24.3	TP1610A11	TP1609H0	29	2,917,011	0.587422026433351	6	C11, C13, C17 , C18, C22, C24	[illegible]		[illegible]	[illegible]
17	1002	624682	0	0.04305556	p13.3	TP1710G01	TP1704A0	5	0.62368	0.333618542316237	3	C11, C17 , C22	[illegible]		[illegible]	[illegible]
17	832671	1730138	0.05763889	0.09236111	p13.3	TP1707D10	TP1709C1	16	0.897467	0.341612037608913	3	C11, C17 , C22	[illegible]	hsa-mir-22	[illegible]	[illegible]
17	4326386	5083694	4:33	5:08	p13.2	TP1703G12	TP1710B0	11	0.757308	0.294104245985598	3	C11, C17 , C22	[illegible]		[illegible]	[illegible]
17	6879623	8230711	0.31111111	8:23	p13.1	TP1704F05	TP1707F0	15	1,351,088	0.318138889281235	3	C11, C17 , C22	[illegible]	hsa-mir-195, hsa-mir-497, hsa-mir-324	[illegible]	[illegible]
17	16917282	18377155	0.73055556	18:38	p11.2	TP1706G12	TP1701C0	16	1,459,873	0.408114385076213	5	C11, C17 , C18, C22, C24	[illegible]	hsa-mir-33b	[illegible]	[illegible]

17	25310878	81020949	25:31:00	81:02:00	q11.1-q25.3	TP1709A10	TP1707C0	661	55,710,071	232,960,608,273,114	10	C4, C4LN, C5, C6, C8, C9, C10, C11, C13, C17 , C18, C21, C22, C24, MPC600, MPC298	WSB1, KSR1, LGALS9, NOS2, NLK, PPY2, TMEM97, IFT20, TNFAIP1, POLDIP2, SARM1, VTN, SLC46A1, SLC13A2, FOXN1, UNC119, PIGS, ALDOC, SPAG5, KIAA0100, SDF2, SUPT6H, PROCA1, RAB34, RPL23A, TLCD1, NEK8, TRAF4, ERAL1, FLOT2, DHRS13, PHF12, SEZ6, PIPOX, TIAF1, MYO18A, CRYBA1, NUFIP2, TAOK1, ABHD15, TP53I13, GIT1, ANKRD13B, CORO6, SSH2, EFCAB5, CCDC55, SLC6A4, BLMH, TMIGD1, CPD, GOSR1, TBC1D29, CRLF3, ATAD5, C17orf42, ADAP2, RNF135, NF1, OMG, EVI2B, EVI2A, , RAB11FIP4, C17orf79, UTP6, SUZ12, LRRC37B, RHOT1, RHBDL3, C17orf75, ZNF207, PSMD11, CDK5R1, MYO1D, TMEM98, SPACA3, ACCN1, CCL2, CCL7, CCL11, CCL8, CCL13, CCL1, C17orf102, TMEM132E, CCT6B, ZNF830, LIG3, RFFL, RAD51L3, FNDC8, NLE1, UNC45B, AMAC1, SLFN5, SLFN11, SLFN12, SLFN13, SLFN12L, SLFN14, PEX12, AP2B1, RASL10B, GAS2L2, C17orf50, MMP28, TAF15, C17orf66, CCL5, RDM1, LYZL6, CCL16, CCL14, CCL15, CCL23, CCL18, CCL3, CCL4, TBC1D3F, CCL3L1, CCL4L1, TBC1D3C, CCL4L2, TBC1D3G, ZNHIT3, PIGW, GGNBP2, DHRS11, MRM1, LHX1, AATF, ACACA, C17orf78, TADA2L, DUSP14, AP1GBP1, DDX52, HNF1B, TBC1D3, TBC1D3E, MRPL45, GPR179, SOCS7, C17orf96, MLLT6, CISD3, PCGF2, PSMB3, PIP4K2B, CCDC49, C17orf98, RPL23, LASP1, FBXO47, PLXDC1, CACNB1, RPL19, STAC2, FBXL20, MED1,	hsa-mir-451, hsa-mir-144, hsa-mir-423, hsa-mir-193a, hsa-mir-365-2, hsa-mir-632, hsa-mir-152, hsa-mir-1203, hsa-mir-10a, hsa-mir-196a-1, hsa-mir-142, hsa-mir-454, hsa-mir-301a, hsa-mir-21, hsa-mir-633, hsa-mir-634, hsa-mir-548d-2, hsa-mir-635, hsa-mir-636, hsa-mir-657, hsa-mir-338, hsa-mir-1250	[illegible]	[illegible]
18	20492631	21668532	20:49	0.92152778	q11.2	TP1805A01	TP1804H0	11	1,175,901	0.221902283821002	3	C10, C11, C24	RBBP8, CABLES1, C18orf45, RIOK3, C18orf8, NPC1, ANKRD29, LAMA3, TTC39C		[illegible]	[illegible]
18	43385615	46642103	43:39:00	46.64	q12.3-q21.1	TP1801D04	TP1806B0	33	3,256,488	0.675609712865275	6	C9, C10, C11, C13, C24, MPC298, MPC960	SIGLEC15, KIAA1632, PSTPIP2, ATP5A1, HAUS1, C18orf25, RNF165, LOXHD1, ST8SIA5, PIAS2, KATNAL2, TCEB3CL2, TCEB3CL, TCEB3C, TCEB3B, HDHD2, IER3IP1, SMAD2, ZBTB7C, C18orf12, KIAA0427, SMAD7, DYM		[illegible]	[illegible]
18	76235066	76435024	76:24:00	76:44:00	q23	TP1803B06	TP1801E0	3	0.199958	0.553837676417986	3	C9, C24, MPC298			[illegible]	[illegible]
18	76651842	77875127	76.65	77.88	q23	TP1015G12	TP1804G0	16	1,223,285	0.368114810306816	6	C10, C11, C13, C18, C24, MPC298	SALL3, ATP9B, NFATC1, CTDP1, KCNG2, PQLC1, TXNL4A, C18orf22, ADNP2		[illegible]	[illegible]
19	124843	8687198	0:12	0.38125	p13.3-p13.2	TP1409H01	TP1610G0	90	8,562,355	0.517701771927568	8	C5, C10, C11, C13, C17 , C18, C22, C24, MPC298	PPAP2C, MIER2, THEG, FAM148C, SHC2, ODF3L2, MADCAM1, C19orf20, CDC34, GZMM, BSG, HCN2, POLRMT, FGF22, RNF126, FSTL3, PRSSL1, PALM, C19orf21, PTBP1, AZU1, PRTN3, ELANE, CFD, MED16, C19orf22, KISS1R, ARID3A, WDR18, GRIN3B, C19orf6, CNN2, ABCA7, HMHA1, POLR2E, GPX4, SBNO2, STK11, C19orf26, ATP5D, MIDN, C19orf23, CIRBP, C19orf24, MUM1, EFNA2, NDUFS7, GAMT, DAZAP1, RPS15, APC2, C19orf25, PCSK4, REEP6, ADAMTSL5, PLK5P, MEX3D, MBD3, UQCR, TCF3, ONECUT3, ATP8B3, REXO1, KLF16, FAM108A1, SCAMP4, ADAT3, CSNK1G2, C19orf34, BTBD2, MKNK2, MOBKL2A, C19orf36, AP3D1, DOT1L, PLEKHJ1, SF3A2, AMH, JSRP1, OAZ1, C19orf35, LINGO3, LSM7,TMPRSS9, TIMM13, LMNB2, GADD45B, DIRAS1, SLC39A3, SGTA, THOP1, ZNF554, ZNF555, ZNF556, ZNF77, ZNF57, TLE6, AES, GNA11, GNA15, S1PR4, NCLN, BRUNOL5, NFIC, DOHH, RAX2, FZR1, C19orf28, C19orf71, HMG20B, GIPC3, C19orf64, TBXA2R, C19orf29OS, PIP5K1C, TJP3, APBA3, MRPL54, MATK, ZFR2, ATCAY, ITGB1BP3, DAPK3, EEF2, PIAS4, ZBTB7A, MAP2K2, CREB3L3, SIRT6, ANKRD24, EBI3, CCDC94, SHD, TMIGD2, FSD1, STAP2, MPND, SH3GL1, CHAF1A, UBXN6, , KIAA1881, LRG1, SEMA6B, TNFAIP8L1, C19orf10, FEM1A, TICAM1, M6PRBP1, ARRDC5, C19orf31, KDM4B, PTPRS, ZNRF4, SAFB2, SAFB, C19orf70, HSD11B1L, RPL36, LONP1,	hsa-mir-1909, hsa-mir-1227, hsa-mir-637, hsa-mir-7-3, hsa-mir-220b	[illegible]	[illegible]
19	10189325	11998892	10:19	12	p13.2	TP1901H09	TP1901H0	23	1,809,567	0.398196889052288	4	C11, C17 , C22, C24	C19orf66, ANGPTL6, PPAN, EIF3G, DNMT1, S1PR2, MRPL4, ICAM1, ICAM4, ICAM5, FDX1L, RAVER1, ICAM3, TYK2, CDC37, PDE4A, KEAP1, S1PR5, ATG4D, KRI1, CDKN2D, AP1M2, SLC44A2, ILF3, QTRT1, DNM2, TMED1, C19orf38, CARM1, YIPF2, C19orf52, SMARCA4, LDLR, SPC24, KANK2, DOCK6, TSPAN16, RAB3D, TMEM205, C19orf39, EPOR, RGL3, CCDC151, PRKCSH, ELAVL3, ZNF653, ECSIT, CNN1, ELOF1, ACP5, ZNF627, ZNF823, ZNF441, ZNF491, ZNF440, ZNF439, ZNF69	hsa-mir-1181, hsa-mir-1238, hsa-mir-638, hsa-mir-199a-1	[illegible]	[illegible]
19	16059937	16662066	16:06	0.7125	p13.12-p13.11	TP1903E02	TP1904B1	5	0.602129	0.369027069251275	3	C9, C11, C24	OR10H4, TPM4, RAB8A, HSH2D, CIB3, FAM32A, AP1M1, KLF2, EPS15L1, CALR3, C19orf44, CHERP, SLC35E1		[illegible]	[illegible]
19	17349068	19912960	17:35	0.85486111	p13.11	TP1905E11	TP1908H1	37	2,563,892	0.360955868275793	5	C11, C13, C17 , C18, C24	NR2F6, USHBP1, ANKLE1, ABHD8, MRPL34, DDA1, ANO8, GTPBP3, PLVAP, BST2, FAM125A, TMEM221, NXNL1, SLC27A1, PGLS, FAM129C, GLT25D1, UNC13A, MAP1S, FCHO1, B3GNT3, JAK3, RPL18A, SLC5A5, CCDC124, KCNN1, ARRDC2, IL12RB1, MAST3, PIK3R2, IFI30, MPV17L2, RAB3A, PDE4C, KIAA1683, JUND, LSM4, PGPEP1, GDF15, LRRC25, SSBP4, ISYNA1, ELL, FKBP8, C19orf50, C19orf60, CRLF1, TMEM59L, KLHL26, CRTC1, COMP, UPF1, GDF1, COPE, DDX49, HOMER3, SFRS14, ARMC6, SLC25A42, TMEM161A, MEF2B, RFXANK, NR2C2AP, NCAN, HAPLN4, TM6SF2, SF4, KIAA0892, GATAD2A, TSSK6, NDUFA13, CILP2, PBX4, LPAR2, GMIP, ATP13A1, ZNF101, ZNF14	hsa-mir-640	[illegible]	[illegible]
19	27797078	28296632	27:08:00	28:03:00	q11	TP1906A02	TP1906C0	6	0.499554	0.341459154445495	3	C4, C6, C21			[illegible]	[illegible]
19	30102080	30525340	30:01:00	30:53:00	q12	TP1903A02	TP1906D0	5	0.42326	0.29607361820357	4	C13, C17 , C18, C22	POP4, PLEKHF1, C19orf12, CCNE1, C19orf2		[illegible]	[illegible]
19	38442569	43016302	38:44:00	43:02:00	q13.13-q13.2	TP1908C02	TP1903A0	64	4,573,733	0.407985507594435	4	C11, C17 , C18, C22	SIPA1L3, DPF1, PPP1R14A, SPINT2, C19orf33, YIF1B, KCNK6, C19orf15, PSMD8, GGN, SPRED3, FAM98C, RASGRP4, RYR1, MAP4K1, EIF3K, ACTN4, CAPN12, LGALS7, LGALS7B, LGALS4, ECH1, HNRNPL, RINL, SIRT2, NFKBIB, FBXO17, MRPS12, FBXO27, PAK4, NCCRP1, SYCN, IL28B, IL28A, IL29, LRFN1, GMFG, SAMD4B, PAF1, MED29, ZFP36, PLEKHG2, RPS16, SUPT5H, TIMM50, DLL3, EID2B, EID2, LGALS13, LGALS14, CLC, LEUTX, DYRK1B, FBL, FCGBP, PSMC4, ZNF546, ZNF780B, ZNF780A, MAP3K10, TTC9B, CNTD2, AKT2, C19orf47, PLD3, HIPK4, PRX, SERTAD1, SERTAD3, BLVRB, SPTBN4, SHKBP1, LTBP4, NUMBL, ADCK4, ITPKC, C19orf54, SNRPA, MIA, RAB4B, EGLN2, CYP2A7, CYP2B6, CYP2A7P1, CYP2A13, CYP2F1, CYP2S1, AXL, HNRNPUL1, CCDC97, TGFB1, B9D2, TMEM91, EXOSC5, B3GNT8, ATP5SL, C19orf69, CEACAM21, CEACAM4, CEACAM7, CEACAM5, CEACAM6, CEACAM3, LYPD4, DMRTC2, RPS19, CD79A, ARHGEF1, RABAC1, ATP1A3, GRIK5, ZNF574, POU2F2, DEDD2, ZNF526, GSK3A, ERF, CIC, PAFAH1B3, PRR19, TMEM145, MEGF8, CNFN, LIPE, CXCL17, CEACAM1	hsa-mir-641	[illegible]	[illegible]
19	45106386	51621674	45:11:00	51.62	q13.31-q13.41	TP1903A11	TP1901A0	91	6,515,288	0.639230203170743	6	C11, C13, C17 , C18, C22, C24	PVR, CEACAM19, CEACAM16, BCL3, CBLC, BCAM, PVRL2, TOMM40, APOE, APOC1, APOC4, APOC2, CLPTM1, RELB, SFRS16, ZNF296, GEMIN7, LRRC68, NKPD1, TRAPPC6A, BLOC1S3, EXOC3L2, MARK4, CKM, KLC3, ERCC2, PPP1R13L, CD3EAP, ERCC1, FOSB, RTN2, VASP, OPA3, GPR4, EML2, GIPR, SNRPD2, QPCTL, FBXO46, SIX5, DMPK, DMWD, RSHL1, SYMPK, FOXA3, IRF2BP1, MYPOP, NANOS2, NOVA2, CCDC61, PGLYRP1, IGFL4, IGFL3, IGFL2, HIF3A, PPP5C, CCDC8, PNMAL1, PNMAL2, CALM3, PTGIR, GNG8, DACT3, PRKD2, STRN4, FKRP, SLC1A5, AP2S1, GRLF1, NPAS1, TMEM160, ZC3H4, SAE1, BBC3, CCDC9, C5AR1, GPR77, DHX34, MEIS3, SLC8A2, KPTN, NAPA, ZNF541, GLTSCR1, EHD2, GLTSCR2, SEPW1, TPRX1, CRX, SULT2A1, BSPH1, ELSPBP1, CABP5, CABP3, PLA2G4C, LIG1, C19orf68, CARD8, ZNF114, CCDC114, EMP3, TMEM143, SYNGR4, KDELR1, GRIN2D, GRWD1, KCNJ14, CYTH2, LMTK3, SULT2B1, FAM83E, SPACA4, RPL18, SPHK2, DBP, CA11, NTN5, FUT2, MAMSTR, RASIP1, IZUMO1, FUT1, FGF21, BCAT2, HSD17B14, PLEKHA4, PPP1R15A, TULP2, NUCB1, DHDH, BAX, FTL, GYS1, RUVBL2, LHB, CGB, CGB2, CGB1, NTF6B, CGB5, CGB8, CGB7, NTF4, KCNA7, SNRNP70, LIN7B, C19orf73, PPFIA3, HRC, TRPM4, SLC6A16, CD37, TEAD2, DKKL1, CCDC155, PTH2, SLC17A7, PIH1D1, ALDH16A1, FLT3LG, RPL13A, RPS11, FCGRT, RCN3, NOSIP, PRRG2,	hsa-mir-330, hsa-mir-642, hsa-mir-769, hsa-mir-220c, hsa-mir-150	[illegible]	[illegible]
19	54081620	54741521	54:08:00	54.74	q13.42	TP1905A01	TP1904E1	12	0.659901	0.278532445727914	3	C11, C17 , C18	ZNF331, DPRX, NLRP12, MYADM, PRKCG, CACNG7, CACNG8, CACNG6, VSTM1, NDUFA3, OSCAR, TFPT, PRPF31, CNOT3, LENG1, TMC4, MBOAT7, TSEN34, RPS9, LILRB3	hsa-mir-512-1, hsa-mir-512-1, hsa-mir-1323, hsa-mir-498, hsa-mir-520e, hsa-mir-515-1, hsa-mir-519e, hsa-mir-520f, hsa-mir-515-1, hsa-mir-519c, hsa-mir-1283-1, hsa-mir-520a, hsa-mir-526b, hsa-mir-519b, hsa-mir-525, hsa-mir-523, hsa-mir-518f, hsa-mir-520b, hsa-mir-518b, hsa-mir-526a-1, hsa-mir-520c, hsa-mir-518c, hsa-mir-524, hsa-mir-517a, hsa-mir-519d, hsa-mir-521-2, hsa-mir-520d, hsa-mir-517b, hsa-mir-520g, hsa-mir-516b-2, hsa-mir-526a-2, hsa-mir-518e, hsa-mir-518a-1, hsa-mir-518d, hsa-mir-516b-1, hsa-mir-518a-2, hsa-mir-517c, hsa-mir-520h, hsa-mir-521-1, hsa-mir-522, hsa-mir-519a-1, hsa-mir-527, hsa-mir-516a-1, hsa-mir-1283-2, hsa-mir-516a-2, hsa-mir-519a-2, hsa-mir-371, hsa-mir-372, hsa-mir-373, hsa-mir-935	[illegible]	[illegible]
19	55821629	56148436	55.82	56:15:00	q13.42	TP1908G08	TP1906A0	3	0.326807	0.33085689808496	3	C11, C17 , C18	BRSK1, TMEM224, SUV420H2, COX6B2, FAM71E2, IL11, TMEM190, RPL28, UBE2S, ISOC2, ZNF628, NAT14, SBK2, ZNF579, FIZ1, ZNF524, ZNF784		[illegible]	[illegible]
19	58575280	59077721	58:58:00	59:08:00	q13.43	TP1908A06	TP1908H0	6	0.502441	0.317280040456992	4	C11, C13, C17 , C18	ZNF135, ZSCAN18, ZNF329, ZNF274, ZNF544, ZNF8, ZSCAN22, A1BG, ZNF497, ZNF837, RPS5, ZNF324B, ZNF132, ZNF324, ZNF446, SLC27A5, ZBTB45, TRIM28, CHMP2A, UBE2M, MZF1		[illegible]	[illegible]
20	2869480	8758526	0.14375	0.38611111	p13-p12.3	TP2003E09	TP2004D0	63	5,889,046	0.476713988752898	3	C10, C17 , C21	PTPRA, GNRH2, MRPS26, OXT, AVP, UBOX5, FASTKD5, DDRGK1, ITPA, SLC4A11, C20orf194, ATRN, GFRA4, ADAM33, SIGLEC1, HSPA12B, C20orf27, SPEF1, CENPB, CDC25B, C20orf29, MAVS, PANK2, RNF24, SMOX, ADRA1D, PRNP, PRND, PRNT, RASSF2, SLC23A2, C20orf30, PCNA, CDS2, PROKR2, C20orf196, CHGB, TRMT6, MCM8, CRLS1, LRRN4, FERMT1, BMP2, HAO1, TMX4, PLCB1	hsa-mir-103-2	[illegible]	[illegible]
20	13926859	26046190	0.60625	26:05:00	p12.1-p11.1	TP2002G12	TP2008F0	122	12,119,331	11,441,387,069,432	5	C10, C11, C17 , C18, C21, C22	SEL1L2, MACROD2, FLRT3, KIF16B, SNRPB2, OTOR, PCSK2, BFSP1, DSTN, RRBP1, BANF2, SNX5, C20orf72, OVOL2, CSRP2BP, ZNF133, C20orf12, POLR3F, RBBP9, SEC23B, HARS2, C20orf78, C20orf79, SLC24A3, , RIN2, NAT5, CRNKL1, C20orf26, INSM1, C20orf74, NCRNA00153, XRN2, NKX2-4, NKX2-2, PAX1, FOXA2, SSTR4, THBD, CD93, NXT1, GZF1, NAPB, CSTL1, CST11, CST8, CST9L, CST9, CST3, CST4, CST1, CST2, CST5, GGTLC1, C20orf39, CST7, C20orf3, ACSS1, VSX1, ENTPD6, PYGB, ABHD12, GINS1, NINL, NANP, ZNF337, FAM182B, FAM182A		[illegible]	[illegible]
20	30024259	62964520	30:02:00	62.96	q11.21-q13.33	TP2006B06	TP2004A0	390	32,940,261	145,558,956,277,928	11	C4, C4LN, C5, C9, C10, C11, C13, C17 , C18, C21, C22, C24, MPC600, MPC298	DEFB123, DEFB124, REM1, HM13, ID1, COX4I2, BCL2L1, TPX2, MYLK2, FOXS1, DUSP15, TTLL9, PDRG1, XKR7, C20orf160, HCK, TM9SF4, PLAGL2, POFUT1, KIF3B, ASXL1, C20orf112, COMMD7, DNMT3B, MAPRE1, EFCAB8, SPAG4L, BPIL1, BPIL3, C20orf185, C20orf186, C20orf70, C20orf71, PLUNC, C20orf114, CDK5RAP1, SNTA1, CBFA2T2, NECAB3, C20orf144, C20orf134, E2F1, PXMP4, ZNF341, CHMP4B, RALY, EIF2S2, AHCY, ASIP, ITCH, DYNLRB1, MAP1LC3A, PIGU, TP53INP2, NCOA6, GGT7, ACSS2, GSS, MYH7B, TRPC4AP, EDEM2, PROCR, EIF6, MMP24, FAM83C, UQCC, GDF5OS, GDF5, CEP250, C20orf173, ERGIC3, SPAG4, CPNE1, ROMO1, RBM39, PHF20, SCAND1, C20orf152, EPB41L1, C20orf4, DLGAP4, MYL9, TGIF2, C20orf24, SLA2, NDRG3, DSN1, C20orf117, C20orf118, SAMHD1, RBL1, C20orf132, RPN2, GHRH, MANBAL, SRC, BLCAP, NNAT, CTNNBL1, VSTM2L, KIAA0406, RPRD1B, TGM2, KIAA1755, BPI, LBP, KIAA1219, ADIG, C20orf95, SLC32A1, ACTR5, PPP1R16B, FAM83D, DHX35, MAFB, TOP1, PLCG1, ZHX3, LPIN3, EMILIN3, CHD6, PTPRT, SFRS6, L3MBTL, SGK2, IFT52, MYBL2, GTSF1L, TOX2, JPH2, C20orf111, GDAP1L1, FITM2, R3HDML, HNF4A, C20orf62, TTPAL, SERINC3, PKIG, ADA, WISP2, KCNK15, RIMS4, YWHAB, PABPC1L, TOMM34, STK4, KCNS1, WFDC5, WFDC12, PI3, SEMG1, SEMG2, SLPI, MATN4, RBPJL, SDC4, SYS1, TP53TG5,	hsa-mir-1825, hsa-mir-644, hsa-mir-499, hsa-mir-1289-1, hsa-mir-645, hsa-mir-1302-5, hsa-mir-296, hsa-mir-298, hsa-mir-646, hsa-mir-1257, hsa-mir-1-1, hsa-mir-133a-2, hsa-mir-124-3, hsa-mir-941-1, hsa-mir-941-1, hsa-mir-941-1, hsa-mir-941-1, hsa-mir-1914, hsa-mir-647	[illegible]	[illegible]
21	16196185	16904300	16:02	16:09	q11.2-q21.1	TP0228C12	TP0718H0	6	0.708115	0.421534656541652	3	C9, C21, MPC600	NRIP1		[illegible]	[illegible]
21	39974761	40466852	39.97	40:47:00	q22.2	TP0718H09	TP1410F0	4	0.492091	0.41721562886207	3	C21, C24, MPC600	ERG, ETS2, C21orf104		[illegible]	[illegible]
21	42543050	48102348	42:54:00	48:01:00	q22.2-q22.3	TP0718H11	TP0620H0	72	5,559,298	0.987056350001611	6	C8, C9, C10, C11, C13, C18, C21, C24	BACE2, FAM3B, MX2, MX1, TMPRSS2, RIPK4, PRDM15, C2CD2, ZNF295, UMODL1, C21orf128, ABCG1, TFF3, TFF2, TFF1, TMPRSS3, UBASH3A, RSPH1, SLC37A1, PDE9A, WDR4, NDUFV3, PKNOX1, CBS, U2AF1, CRYAA, C21orf136, SIK1, HSF2BP, RRP1B, PDXK, CSTB, RRP1, AGPAT3, TRAPPC10, C21orf33, C21orf32, ICOSLG, DNMT3L, AIRE, PFKL, C21orf2, TRPM2, LRRC3, C21orf30, C21orf29, C21orf90, KRTAP10-1, KRTAP10-2, KRTAP10-3, KRTAP10-4, KRTAP10-6, KRTAP10-7, KRTAP10-8, KRTAP10-9, KRTAP10-10, KRTAP10-11, KRTAP12-4, KRTAP12-2, KRTAP12-1, KRTAP10-12, UBE2G2, SUMO3, PTTG1IP, ITGB2, C21orf67, C21orf69, C21orf70, ADARB1, POFUT2, C21orf86, COL18A1, NCRNA00175, SLC19A1, PCBP3, COL6A1, COL6A2, FTCD, C21orf56, LSS, MCM3AP, C21orf57, C21orf58, PCNT, DIP2A, S100B, PRMT2		[illegible]	[illegible]
22	16061103	16505264	16:06	16:51	q11.1	TP1410H01	TP0620D1	13	0.444161	0.568164752822507	4	C5, C21, MPC298, MPC960	POTEH, OR11H1		[illegible]	[illegible]
22	19880186	21949089	0.85277778	0.94097222	q11.21	TP0125D11	TP1312E0	19	2,068,903	0.324961348563814	4	C11, C13, C22, C24	TXNRD2, COMT, ARVCF, C22orf25, DGCR8, TRMT2A, RANBP1, ZDHHC8, RTN4R, DGCR6L, GGTLC3, TMEM191B, RIMBP3, USP41, ZNF74, KLHL22, MED15, POM121L4P, TOP3B, SERPIND1, SNAP29, CRKL, AIFM3, LZTR1, THAP7, P2RX6, SLC7A4, POM121L7, GGT2, RIMBP3B, HIC2, RIMBP3C, UBE2L3	hsa-mir-185, hsa-mir-1306, hsa-mir-1286, hsa-mir-649	[illegible]	[illegible]
22	50053636	51186519	50:05:00	51:19:00	q13.33	TP0125G07	TP0001G0	12	1,132,883	0.314039620427027	7	C11, C13, C17 , C18, C22, MPC298, MPC960	BRD1, ZBED4, ALG12, CRELD2, PIM3, IL17REL, TTLL8, MLC1, MOV10L1, PANX2, TRABD, TUBGCP6, HDAC10, MAPK12, MAPK11, PLXNB2, FAM116B, SAPS2, SBF1, ADM2, MIOX, LMF2, NCAPH2, SCO2, TYMP, ODF3B, KLHDC7B, C22orf41, CPT1B, CHKB, MAPK8IP2, ARSA, SHANK3, ACR		[illegible]	[illegible]
23	152443438	155031692	152:44:00	155:03:00	q28	TPX012A12	TPX004C0	31	2,588,254	0.449763874668613	5	C5, C8, C17 , C18, C21, C22, C24, MPC600	MAGEA1, ZNF275, TREX2, HAUS7, BGN, ATP2B3FAM58A, DUSP9, PNCK, SLC6A8, BCAP31, ABCD1, PLXNB3, SRPK3, IDH3G, SSR4, PDZD4, L1CAM, AVPR2, ARHGAP4, ARD1A, RENBP, HCFC1, TMEM187, IRAK1, MECP2, OPN1LW, TEX28P2, OPN1MW, TEX28P1, OPN1MW2, TEX28, TKTL1, FLNA, EMD, RPL10, DNASE1L1, TAZ, ATP6AP1, GDI1, FAM50A, PLXNA3, LAGE3, UBL4A, SLC10A3, FAM3A, G6PD, IKBKG, CXorf52, CTAG1A, CTAG1B, CXorf52B, CTAG2, GAB3, DKC1, MPP1, F8, H2AFB1, F8A1, FUNDC2, MTCP1NB, BRCC3, VBP1, RAB39B, CLIC2, H2AFB2, F8A2, F8A3, H2AFB3, TMLHE, SPRY3	hsa-mir-1184, hsa-mir-1184, hsa-mir-1184	[illegible]	[illegible]

LOSSES (in 3 or more cases)

Chrom	Start	End	Start MB	End MB	Cytobands	Start Probe	End Probe	Probes	Length MB	maxM	max.overlap	Cases	Genes	miRNAs	aCGH CNVs	Full CNVs
1	11754	35407496	0:01	35:41:00	p36.33-p34.3	TP0121D10	TP0114C0	377	35,395,742	-0.738498442024359	12	C4, C4LN, C5, C8, C9, C10, C11, C13, C17 , C18, C22, C24, MPC600, MPC298, MPC960	WASH5P, OR4F5, OR4F29, OR4F16, SAMD11, NOC2L, KLHL17, PLEKHN1, C1orf170, HES4, ISG15, AGRN, C1orf159, TTLL10, TNFRSF18, TNFRSF4, SDF4, B3GALT6, FAM132A, UBE2J2, SCNN1D, ACAP3, PUSL1, CPSF3L, GLTPD1, TAS1R3, DVL1, MXRA8, AURKAIP1, CCNL2, MRPL20, VWA1, ATAD3C, ATAD3B, ATAD3A, C1orf70, SSU72, MIB2, MMP23B, CDC2L2,SLC35E2, NADK, GNB1, CALML6, TMEM52, C1orf222, KIAA1751, GABRD, PRKCZ, C1orf86, SKI, MORN1, RER1, PEX10, PLCH2, PANK4, HES5, TNFRSF14, C1orf93, MMEL1, TTC34, ACTRT2, PRDM16, ARHGEF16, MEGF6, TPRG1L, WDR8, TP73, CCDC27, LRRC47, KIAA0562, DFFB, C1orf174, AJAP1,NPHP4, KCNAB2, CHD5, RPL22, RNF207, ICMT, HES3, GPR153, ACOT7, HES2, ESPN, TNFRSF25, PLEKHG5, NOL9, TAS1R1, ZBTB48, KLHL21, PHF13, THAP3, DNAJC11, CAMTA1, VAMP3, PER3, UTS2, TNFRSF9, PARK7, ERRFI1, SLC45A1, RERE, ENO1, CA6, SLC2A7, SLC2A5, GPR157, H6PD, SPSB1, SLC25A33, TMEM201, PIK3CD, C1orf200, CLSTN1,CTNNBIP1, LZIC, NMNAT1, RBP7, UBE4B, KIF1B, PGD, APITD1, DFFA, PEX14, CASZ1, C1orf127, TARDBP, MASP2, SRM, EXOSC10, FRAP1, ANGPTL7, UBIAD1, PTCHD2, FBXO2, FBXO44, FBXO6, MAD2L2, C1orf187, AGTRAP, C1orf167, MTHFR, CLCN6, NPPA, NPPB, KIAA2013, PLOD1, MFN2, MIIP, TNFRSF8, TNFRSF1B, VPS13D, DHRS3,	hsa-mir-1302-2, hsa-mir-1977, hsa-mir-200b, hsa-mir-200a, hsa-mir-429, hsa-mir-551a, hsa-mir-34a, hsa-mir-1290, hsa-mir-1256, hsa-mir-1976, hsa-mir-552	[illegible]	[illegible]
1	35654872	39486811	35.65	39:49:00	p34.3	TP0105E07	TP0111D1	43	3,831,939	-0.656260623004575	7	C8, C9, C13, C17 , C22, MPC600, MPC960	SFPQ, ZMYM4, KIAA0319L, NCDN, PSMB2, TFAP2E, C1orf216, CLSPN, EIF2C4, EIF2C1, EIF2C3, TEKT2, ADPRHL2, COL8A2, TRAPPC3, MAP7D1, THRAP3, C1orf113, FAM176B, STK40, LSM10, C1orf102, MRPS15, CSF3R, GRIK3, ZC3H12A, C1orf149, SNIP1, DNALI1, GNL2, RSPO1, C1orf109, CDCA8, EPHA10, MANEAL, YRDC, C1orf122, MTF1, INPP5B, SF3A3, FHL3, UTP11L, POU3F1, RRAGC, MYCBP, GJA9, RHBDL2, AKIRIN1		[illegible]	[illegible]
1	41397252	43044104	41:04:00	43:04:00	p34.2	TP0109B08	TP0104B0	14	1,646,852	-0.528057394451977	3	C8, C17 , MPC600	CTPS, SLFNL1, SCMH1, FOXO6, EDN2, HIVEP3, GUCA2B, GUCA2A, FOXJ3, RIMKLA, ZMYND12, PPCS		[illegible]	[illegible]
1	44351052	120529415	44:35:00	120:53:00	p34.1-p12	TP0123H09	TP0110B0	792	76,178,363	-0.683898120270863	11	C4, C4LN, C8, C9, C10, C11, C13, C17 , C18, C22, MPC600, MPC960	ST3GAL3, ARTN, IPO13, DPH2, ATP6V0B, B4GALT2, CCDC24, SLC6A9, KLF17, DMAP1, ERI3, RNF220, TMEM53, KIF2C, RPS8, BEST4, PLK3, TCTEX1D4, PTCH2, EIF2B3, HECTD3, UROD, ZSWIM5, HPDL, MUTYH, TOE1, TESK2, MMACHC, PRDX1, AKR1A1, NASP, CCDC17, GPBP1L1, TMEM69, IPP, MAST2, PIK3R3, TSPAN1, POMGNT1, C1orf190, RAD54L, LRRC41, UQCRH, NSUN4, FAAH, DMBX1, KNCN, MKNK1, MOBKL2C, ATPAF1, KIAA0494, CYP4B1, CYP4A11, CYP4X1, CYP4Z1, CYP4A22, PDZK1IP1, TAL1, STIL, CMPK1, FOXE3, FOXD2, SLC5A9, SPATA6, AGBL4, BEND5, ELAVL4, DMRTA2, FAF1, CDKN2C, C1orf185, RNF11, TTC39A, EPS15, OSBPL9, NRD1, RAB3B, TXNDC12, KTI12, BTF3L4, ZFYVE9, CC2D1B, ORC1L, PRPF38A, ZCCHC11, GPX7, C1orf163, ZYG11B, ZYG11A, ECHDC2, SCP2, PODN, SLC1A7, CPT2, C1orf123, MAGOH, LRP8, DMRTB1, GLIS1, TMEM48, YIPF1, DIO1, HSPB11, LRRC42, LDLRAD1, TMEM59, C1orf83, CDCP2, CYB5RL, MRPL37, SSBP3, C1orf191, ACOT11, FAM151A, C1orf175, PARS2, TTC22, C1orf177, DHCR24, TMEM61, BSND, PCSK9, USP24, PPAP2B, PRKAA2, C1orf168, C8A, C8B, DAB1, OMA1, TACSTD2, MYSM1, JUN, FGGY, HOOK1, CYP2J2, C1orf87, NFIA, TM2D1, INADL, KANK4, USP1, DOCK7, ANGPTL3, ATG4C, FOXD3, ALG6, ITGB3BP, EFCAB7, DLEU2L, PGM1, ROR1, UBE2U, CACHD1, RAVER2, JAK1, AK3L1, DNAJC6,	hsa-mir-101-1, hsa-mir-1262, hsa-mir-186, hsa-mir-760, hsa-mir-137, hsa-mir-553, hsa-mir-197, hsa-mir-320b-1, hsa-mir-942	[illegible]	[illegible]
1	121181443	143544525	121:18:00	143:54:00	p11.2-q21.1	TP0109B09	TP1507G0	19	22,363,082	-0.534569412089262	8	C4, C10, C11, C13, C17 , C18, C22, C24			[illegible]	[illegible]
2	11860437	13351459	0.51805556	13:35	p25.1-p24.3	TP1212G12	TP0207D0	12	1,491,022	-0.403190995735365	3	C5, C17 , C18	LPIN1, TRIB2		[illegible]	[illegible]
2	14604456	15088384	14:06	15:09	p24.3	TP0226F03	TP0214B0	4	0.483928	-0.259616272388105	3	C5, C17 , C18	FAM84A		[illegible]	[illegible]
2	15800132	27623158	15:08	27.62	p24.3-p23.3	TP0212C02	TP0220D1	119	11,823,026	-0.533212857767187	7	C5, C6, C13, C17 , C18, C22, C24, MPC298, MPC960	MYCN, FAM49A, RAD51AP2, VSNL1, SMC6, GEN1, MSGN1, KCNS3, NT5C1B, OSR1, TTC32, WDR35, MATN3, LAPTM4A, SDC1, PUM2, RHOB, HS1BP3, GDF7, C2orf43, APOB,KLHL29, ATAD2B, UBXN2A, C2orf44, FKBP1B, TP53I3, PFN4, C2orf84, ITSN2, NCOA1, C2orf79, CENPO, ADCY3, DNAJC27, EFR3B, POMC, DNMT3A, DTNB, ASXL2, KIF3C, RAB10, FAM59B, HADHA, HADHB, GPR113, C2orf39, OTOF, C2orf70, CIB4, KCNK3, C2orf18, CENPA, DPYSL5, MAPRE3, TMEM214, AGBL5, EMILIN1, KHK, CGREF1, ABHD1, PREB, C2orf53, TCF23, SLC5A6, C2orf28, CAD, SLC30A3, DNAJC5G, TRIM54, UCN, MPV17, GTF3C2, EIF2B4, SNX17, ZNF513, PPM1G	hsa-mir-1301	[illegible]	[illegible]
2	28215006	31923994	28:22:00	31.92	p23.2-p23.1	TP0217G04	TP0207A0	41	3,708,988	-0.433400996729642	3	C5, C18, MPC960	MRPL33, FOSL2, PLB1, PPP1CB, SPDYA, TRMT61B, WDR43, FAM179A, C2orf71, CLIP4, ALK, YPEL5, LBH, LCLAT1, CAPN13, GALNT14, CAPN14, EHD3, XDH, SRD5A2		[illegible]	[illegible]
2	34657446	36143245	34.66	36:14:00	p22.3	TP0223A11	TP0215F0	22	1,485,799	-0.373478103711075	4	C18, C24, MPC298, MPC960			[illegible]	[illegible]
2	39298197	42182467	39:03:00	42:18:00	p22.1-p21	TP0215E07	TP0202F0	29	288,427	-0.371183323486268	4	C13, C18, C22, MPC960	SOS1, CDKL4, MAP4K3, TMEM178, THUMPD2, SLC8A1		[illegible]	[illegible]
2	47147859	48560755	47:15:00	48:56:00	p21-p16.3	TP0201F01	TP0210C0	13	1,412,896	-0.399493583713812	3	C9, C18, MPC960	MCFD2, TTC7A, CALM2, EPCAM, MSH2, KCNK12, MSH6, FBXO11, FOXN2	hsa-mir-559	[illegible]	[illegible]
2	48832073	50087738	48.83	50:09:00	p16.3	TP0208D05	TP0227H0	14	1,255,665	-0.308240865944252	3	C18, C22, MPC960	STON1, LHCGR, FSHR		[illegible]	[illegible]
2	56469773	60685174	56:47:00	60.69	p16.1	TP0220C01	TP0205E0	41	4,215,401	-0.442198844471504	3	C11, C18, C22	CCDC85A, VRK2, FANCL, BCL11A		[illegible]	[illegible]
2	66025873	68050532	66:03:00	68:05:00	p14	TP0220C04	TP0204D0	23	2,024,659	-0.274319181399663	4	C11, C18, C22, C24	MEIS1, ETAA1		[illegible]	[illegible]
2	70731148	71428685	70.73	71:43:00	p13.3	TP0211G08	TP0212G0	8	0.697537	-0.232825720831073	3	C6, C9, C18	TGFA, ADD2, FIGLA, CLEC4F, CD207, VAX2, ATP6V1B1, TEX261, ANKRD53, NAGK, MCEE, MPHOSPH10, PAIP2B		[illegible]	[illegible]
2	76005001	80453533	76:01:00	80:45:00	p12	TP0225A09	TP0217G0	50	4,448,532	-0.251293809024348	3	C11, C18, C22	LRRTM4, REG3G, REG1B, REG1A, REG3A, CTNNA2		[illegible]	[illegible]
2	81275403	85032078	81:28:00	85:03:00	p12-p11.2	TP0208E04	TP0227B0	39	3,756,675	-0.305235339605927	3	C11, C18, C22	SUCLG1, DNAH6		[illegible]	[illegible]
2	87080910	87608239	87:08:00	87.61	p11.2	TP0225F03	TP0211B0	10	0.527329	-0.29856759271697	3	C17 , C18, C24	CD8B, RGPD1, PLGLB1		[illegible]	[illegible]
2	87956698	88258429	87.96	88:26:00	p11.2	TP0213G07	TP0203G0	3	0.301731	-0.325662872917286	3	C17 , C18, C24	PLGLB2, RGPD2		[illegible]	[illegible]
2	88987222	90265836	88.99	90:27:00	p11.2-p11.1	TP0211G09	TP0223G0	9	1,278,614	-0.351079346165815	3	C17 , C18, C24	RPIA, IGKV2-19, IGKC, IGKV4-1, IGKV1-5, IGKV3D-15		[illegible]	[illegible]
2	91877692	95652499	91.88	95.65	q11.1	TP0120C04	TP0209E0	17	3,774,807	-0.440203960007509	4	C17 , C18, C24, MPC960	TEKT4		[illegible]	[illegible]
2	97611213	99159030	97.61	99:16:00	q11.2	TP0207D02	TP0208E1	20	1,547,817	-0.412637329277762	6	C9, C10, C11, C13, C17 , C18, C24	FAM178B, FAHD2B, ANKRD36B, COX5B, ACTR1B, ZAP70, TMEM131, VWA3B, CNGA3, INPP4A		[illegible]	[illegible]
2	99915480	101989117	99.92	101.99	q11.2	TP0202H07	TP0223D0	17	2,073,637	-0.377778554662325	3	C17 , C18, C21	MRPL30, LYG1, TXNDC9, EIF5B, REV1, AFF3, LONRF2, CHST10, NMS, PDCL3, NPAS2, RPL31, TBC1D8, C2orf29, RNF149, CREG2		[illegible]	[illegible]
2	108831404	112758612	108.83	112.76	q12.3-q13	TP0209H11	TP0222A1	39	3,927,208	-0.30129111512823	3	C17 , C18, MPC298	SULT1C3, SULT1C2, SULT1C4, GCC2, LIMS1, RANBP2, CCDC138, EDAR, SH3RF3, ANKRD57, RGPD5, LIMS3, MALL, NPHP1, RGPD6, BUB1, ACOXL, BCL2L11, ANAPC1, MERTK		[illegible]	[illegible]
2	121659718	122097634	121.66	122:01:00	q14.2	TP0218H10	TP0221F1	3	0.437916	-0.226199433641248	3	C17 , C18, MPC298	GLI2, TFCP2L1, CLASP1		[illegible]	[illegible]
2	126717176	127783664	126.72	127.78	q14.3	TP0225F09	TP0227E0	12	1,066,488	-0.218521559624999	3	C9, C17 , C18	GYPC		[illegible]	[illegible]
2	132279581	132994118	132:28:00	132.99	q21.1-q21.2	TP0212A06	TP1011H1	9	0.714537	-0.37250803069263	4	C11, C13, C17 , C18	CCDC74A, C2orf27A, C2orf27B, ZNF72, C21orf99		[illegible]	[illegible]
2	220536177	223352361	220:54:00	223:35:00	q35-q36.1	TP0217D10	TP0215G1	33	2,816,184	-0.344257503949619	4	C10, C17 , C18, C22	EPHA4, PAX3, CCDC140, SGPP2		[illegible]	[illegible]

3	38160054	47681202	38:16:00	47.68	p22.2-p21.31	TP0322D11	TP0315B0	127	9,521,148	-0.472392233553188	3	C5, C18, MPC600	DLEC1, ACAA1, MYD88, OXSR1, SLC22A13, SLC22A14, XYLB, ACVR2B, EXOG, SCN5A, SCN10A, SCN11A, WDR48, GORASP1, TTC21A, CSRNP1, XIRP1, CX3CR1, CCR8, SLC25A38, RPSA, MOBP, MYRIP, EIF1B, ENTPD3, RPL14, ZNF619, ZNF620, ZNF621, CTNNB1, ULK4, TRAK1, CCK, LYZL4, VIPR1, SEC22C, SS18L2, NKTR, ZBTB47, KBTBD5, HHATL, CCDC13, CCBP2, CYP8B1, ZNF662, C3orf39, SNRK, ANO10, ABHD5, C3orf23, ZNF445, ZNF852, ZNF167, ZNF660, ZNF197, ZNF35, ZNF502, ZNF501, KIAA1143, KIF15, TMEM42, TGM4, ZDHHC3, EXOSC7, CLEC3B, CDCP1, LARS2, LIMD1, SACM1L, SLC6A20, LZTFL1, CCR9, FYCO1, CXCR6, XCR1, CCR3, CCR1, CCR2, CCR5, CCRL2, LTF, RTP3, LRRC2, LUZPP1, TDGF1, ALS2CL, TMIE, MYL3, PTH1R, CCDC12, NBEAL2, SETD2, KIF9, KLHL18, PTPN23, SCAP, C3orf75, CSPG5, SMARCC1,	hsa-mir-138-1, hsa-mir-564	[illegible]	[illegible]
3	53313712	56200642	53:31:00	56:02:00	p21.1-p14.3	TP0311C07	TP0314B0	24	288,693	-0.279921931321208	4	C5, C17 , C18, MPC600	DCP1A, CACNA1D, CHDH, IL17RB, ACTR8, CACNA2D3, LRTM1, WNT5A, ERC2		[illegible]	[illegible]
3	59074387	63772647	59:07:00	63.77	p14.2-p14.1	TP0314A07	TP0314G0	50	469,826	-0.508033909417669	3	C5, C18, MPC600	FHIT, NPCDR1, PTPRG, ID2B, C3orf14, FEZF2, CADPS, SYNPR, SNTN		[illegible]	[illegible]
3	63937687	75952765	63.94	75.95	p14.1-p12.3	TP0312C01	TP0319A0	102	12,015,078	-0.551638825454566	6	C5, C6, C8, C13, C18, MPC600, MPC298	ATXN7, PSMD6, PRICKLE2, ADAMTS9, SLC25A26, LRIG1, KBTBD8, SUCLG2, FAM19A1, FAM19A4, C3orf64, TMF1, UBA3, ARL6IP5, LMOD3, FRMD4B, MITF, FOXP1, EIF4E3, GPR27, PROK2, RYBP, SHQ1, GLT8D4, PPP4R2, PDZRN3, CNTN3, FAM86D, FRG2C, ZNF717	hsa-mir-1284, hsa-mir-1324	[illegible]	[illegible]
3	125168241	125760784	125:17:00	125.76	q21.2	TP0303D07	TP0321E1	8	0.592543	-0.322418059253585	7	C9, C10, C13, C18, C24, MPC600, MPC298	SNX4, OSBPL11, OR7E85P, ALG1L, ROPN1B, SLC41A3	hsa-mir-548i-1	[illegible]	[illegible]
3	185208287	190011795	185:21:00	190:01:00	q27.2-q28	TP0319E06	TP0301H1	51	4,803,508	-0.576663238306931	4	C4, C9, C17 , MPC298	TMEM41A, LIPH, SENP2, IGF2BP2, C3orf65, TRA2B, ETV5, CRYGS, TBCCD1, DNAJB11, AHSG, FETUB, HRG, KNG1, EIF4A2, RFC4, ADIPOQ, ST6GAL1, RPL39L, RTP1, MASP1, RTP4, SST, RTP2, BCL6, LPP, TPRG1, TP63, LEPREL1	hsa-mir-28, hsa-mir-944	[illegible]	[illegible]
4	3568752	28378339	3:57	28:38:00	p16.3-p15.1	TP0402F10	TP0406B0	261	24,809,587	-0.781278630381782	9	C4, C4LN, C9, C10, C17 , C18, C24, MPC600, MPC298, MPC960	ADRA2C, OTOP1, TMEM128, LYAR, ZNF509, STX18, MSX1, CYTL1, STK32B, C4orf6, EVC2, EVC, CRMP1, C4orf50, JAKMIP1, WFS1, PPP2R2C, MAN2B2, MRFAP1, S100P, MRFAP1L1, CNO, KIAA0232, TBC1D14, CCDC96, GRPEL1, SORCS2, PSAPL1, AFAP1, ABLIM2, SH3TC1, HTRA3, ACOX3, C4orf23,GPR78, CPZ, HMX1, FAM90A2P, USP17, DEFB131, DRD5, SLC2A9, WDR1, ZNF518B, CLNK, HS3ST1,RAB28, NKX3-2, BOD1L, CPEB2, C1QTNF7, FBXL5, BST1, CD38, FGFBP1, FGFBP2, TAPT1, LDB2, QDPR, CLRN2, LAP3, MED28, FAM184B, C4orf30, NCAPG, LCORL, SLIT2, PACRGL, KCNIP4, GPR125, PPARGC1A, DHX15, SOD3, CCDC149, LGI2, SEPSECS, PI4K2B, ZCCHC4, ANAPC4, SLC34A2, RBPJ, CCKAR, TBC1D19, STIM2	hsa-mir-95, hsa-mir-548i-2, hsa-mir-572, hsa-mir-218-1, hsa-mir-573	[illegible]	[illegible]
4	36814270	37559005	36.81	37:56:00	p14	TP0405B07	TP0406A0	6	0.744735	-0.330947858654515	3	C18, C22, MPC600	KIAA1239, C4orf19		[illegible]	[illegible]
4	49175196	49621245	49:18:00	49.62	p11	TP0419D11	TP0417D0	3	0.446049	-0.439536243349119	4	C4, C13, C17 , C18			[illegible]	[illegible]
4	189943203	190899780	189.94	190:09:00	q35.2	TP0409F03	TP0528A0	15	0.956577	-0.364322129217799	9	C8, C11, C13, C17 , C18, C24, MPC600, MPC298, MPC960	HSP90AA4P, FRG1		[illegible]	[illegible]
5	34060230	34334851	34:06:00	34:33:00	p13.2	TP0501H04	TP0501D0	5	0.274621	-0.2860585871791	3	C11, C13, C18	AMACR		[illegible]	[illegible]
5	75069234	75551949	75:07:00	75:55:00	q13.3	TP0525B09	TP0503C0	13	0.482715	-0.230187089217258	3	C10, C11, C18	SV2C		[illegible]	[illegible]
6	39976474	40932220	39.98	40.93	p21.2-p21.1	TP0614D04	TP0611H0	12	0.955746	-0.398539918503312	3	C9, C10, C17	LRFN2		[illegible]	[illegible]
6	57184980	64970197	57:18:00	64.97	p11.2-q12	TP0613F02	TP0613D0	67	7,785,217	-0.361314509174775	4	C11, C17 , C18, C24	PRIM2, KHDRBS2, FKBP1C, LGSN, PTP4A1, PHF3, EYS		[illegible]	[illegible]
6	72212393	72935149	72:21:00	72.94	q13	TP0614B12	TP0606B0	8	0.722756	-0.27374027206286	3	C17 , C18, C21	RIMS1		[illegible]	[illegible]
6	74665118	135431965	74.67	135:43:00	q13-q23.3	TP0607C05	TP0607G1	687	60,766,847	-0.498110261021648	8	C4, C4LN, C10, C17 , C18, C21, MPC600, MPC298, MPC960	COL12A1, COX7A2, TMEM30A, FILIP1, SENP6, MYO6, IMPG1, HTR1B, IRAK1BP1, PHIP, HMGN3, LCA5, SH3BGRL2, ELOVL4, TTK, BCKDHB, FAM46A, IBTK, TPBG, UBE2CBP, DOPEY1, PGM3, RWDD2A, ME1, PRSS35, SNAP91, RIPPLY2, CYB5R4, MRAP2, KIAA1009, TBX18, NT5E, SNX14, SYNCRIP, FAM165A HTR1E, CGA, ZNF292, GJB7, C6orf162, C6orf163, C6orf164, C6orf165, SLC35A1, RARS2, ORC3L, AKIRIN2, SPACA1, CNR1, RNGTT, PNRC1, PM20D2, GABRR1, GABRR2, UBE2J1, RRAGD, ANKRD6, MDN1, CASP8AP2, GJA10, BACH2, MAP3K7, EPHA7, MANEA, FUT9, KIAA0776, FHL5, GPR63, NDUFAF4, KLHL32, C6orf167, POU3F2, FBXL4, C6orf168, COQ3, SFRS18, USP45, CCNC, PRDM13, MCHR2, SIM1, ASCC3, GRIK2, HACE1, C6orf220, LIN28B, BVES, C6orf112, POPDC3, PREP, PRDM1, ATG5, AIM1, RTN4IP1, QRSL1, C6orf203, BEND3, PDSS2, SOBP, SCML4, SEC63, OSTM1, NR2E1, SNX3, LACE1, FOXO3, ARMC2, SESN1, C6orf182, C6orf184, C6orf185, CD164, PPIL6, SMPD2, MICAL1, ZBTB24, AKD1, FIG4, GPR6, WASF1, CDC40, C6orf186, DDO, SLC22A16, CDC2L6, AMD1, GTF3C6, BXDC1, SLC16A10, KIAA1919, REV3L, TRAF3IP2, FYN, WISP3, TUBE1, C6orf225, LAMA4, MARCKS, HDAC2, HS3ST5, FRK, NT5DC1, COL10A1, TSPYL1, DSE, FAM26F, BET3L, FAM26E, FAM26D, RWDD1, RSPH4A, ZUFSP, KPNA5, FAM162B, GPRC6A, RFX6, VGLL2,	hsa-mir-2113, hsa-mir-587, hsa-mir-548b, hsa-mir-588	[illegible]	[illegible]
6	135848447	148106097	135.85	148:11:00	q23.3-q24.3	TP0607D05	TP0606F0	131	1,225,765	-0.431154453057806	4	C10, C21, C22, MPC298, MPC960	PDE7B, FAM54A, BCLAF1, MAP7, MAP3K5, PEX7, SLC35D3, IL20RA, IL22RA2, IFNGR1, OLIG3, TNFAIP3, PERP, KIAA1244, PBOV1, HEBP2, NHSL1, CCDC28A, ECT2L, REPS1, C6orf115, HECA, TXLNB, CITED2, NMBR, VTA1, GPR126, HIVEP2, AIG1, ADAT2, PEX3, FUCA2, PHACTR2, LTV1, FAM164B, PLAGL1, SF3B5, STX11, UTRN, EPM2A, FBXO30, SHPRH, GRM1, RAB32, C6orf103, STXBP5, SAMD5		[illegible]	[illegible]
6	150212675	150944914	150:21:00	150.94	q25.1	TP0605G01	TP0616C1	6	0.732239	-0.367201995044145	3	C9, C10, MPC960	RAET1G, ULBP2, ULBP1, RAET1L, ULBP3, PPP1R14C, IYD, PLEKHG1		[illegible]	[illegible]
6	151333499	151661042	151:33:00	151.66	q25.1	TP0608A03	TP0603A1	3	0.327543	-0.239858049063745	3	C9, C21, MPC960	MTHFD1L, AKAP12		[illegible]	[illegible]
6	152680933	154810310	152.68	154.81	q25.2	TP0606G08	TP0609H0	31	2,129,377	-0.331553829109149	4	C10, C21, C22, MPC960	SYNE1, MYCT1, VIP, FBXO5, MTRF1L, RGS17, OPRM1, IPCEF1, MAGI1		[illegible]	[illegible]
6	155682407	157530394	155.68	157:53:00	q25.3	TP0616G03	TP0612B0	16	1,847,987	-0.327521051579514	4	C10, C21, C22, MPC960	NOX3, ARID1B	hsa-mir-1202	[illegible]	[illegible]
6	159401361	161653997	159:04:00	161.65	q25.3-q26	TP0608B10	TP0606E0	30	2,252,636	-0.443656310986548	5	C10, C17 , C18, C21, C24, MPC960	RSPH3, TAGAP, FNDC1, SOD2, WTAP, ACAT2, TCP1, MRPL18, PNLDC1, MAS1, IGF2R, SLC22A1, SLC22A2, SLC22A3, LPAL2, LPA, PLG, MAP3K4, AGPAT4		[illegible]	[illegible]
6	163176914	165969091	163:18:00	165.97	q26-q27	TP0602D11	TP0603H0	33	2,792,177	-0.429460497185528	4	C10, C21, MPC298, MPC960	PACRG, QKI, C6orf118, PDE10A		[illegible]	[illegible]
6	166317079	171008924	166:32:00	171:01:00	q27	TP0607B04	TP0603G0	49	4,691,845	-0.45025890768301	5	C10, C21, C22, MPC298, MPC960	T, PRR18, SFT2D1, BRP44L, RPS6KA2, RNASET2, FGFR1OP, CCR6, GPR31, TCP10L2, UNC93A, TTLL2, TCP10, C6orf123, C6orf124, MLLT4, C6orf54, KIF25, FRMD1, DACT2, SMOC2, THBS2, WDR27, C6orf120, PHF10, TCTE3, C6orf70, DLL1, FAM120B, PSMB1, TBP, PDCD2	hsa-mir-1913	[illegible]	[illegible]
7	57519332	61265855	57:52:00	61:27:00	p11.1-q11.21	TP0717B04	TP0717E0	12	3,746,523	-0.314262847980405	5	C4, C17 , C18, C24, MPC960	ZNF716		[illegible]	[illegible]
7	76123925	76537446	76:12:00	76:54:00	q11.23	TP0718B03	TP0707C0	5	0.413521	-0.315611782839889	4	C8, C13, C21, MPC298	UPK3B, POMZP3		[illegible]	[illegible]
7	85995652	87489385	86	87:49:00	q21.11-q21.12	TP0709F02	TP0709A0	15	1,493,733	-0.380791846624878	3	C4, C4LN, MPC298	GRM3, KIAA1324L, DMTF1, C7orf23, CROT, ABCB4, ABCB1, RUNDC3B, SLC25A40		[illegible]	[illegible]
7	91582890	102852516	91:58:00	102.85	q21.2-q22.1	TP0710G05	TP0704G0	104	11,269,626	-0.497346294213793	6	C4LN, C5, C6, C9, C18, MPC298, MPC960	AKAP9, CYP51A1, KRIT1, ANKIB1, GATAD1, PEX1, C7orf64, FAM133B, CDK6, SAMD9, SAMD9L, HEPACAM2, CCDC132, CALCR, GNGT1, TFPI2, GNG11, BET1, COL1A2, CASD1, SGCE, PPP1R9A, PON1, PON3, PON2, ASB4, PDK4, DYNC1I1, SLC25A13, SHFM1, DLX6, DLX5, ACN9, TAC1, ASNS, OCM2, LMTK2, BHLHA15, TECPR1, BRI3, BAIAP2L1, NPTX2, TMEM130, TRRAP, SMURF1, KPNA7, ARPC1B, PDAP1, BUD31, PTCD1, CPSF4, ZNF789, ZNF394, ZKSCAN5, C7orf38, ZNF655, ZNF498, CYP3A5, CYP3A7, CYP3A5P1, CYP3A4, CYP3A43, TRIM4, GJC3, AZGP1, AZGP1P1, ZKSCAN1, ZSCAN21, ZNF3, COPS6, MCM7, AP4M1, TAF6, CNPY4, MBLAC1, C7orf59, C7orf43, GAL3ST4, GPC2, STAG3, GATS, PVRIG, PMS2L1, PILRB, PILRA, ZCWPW1, MEPCE, C7orf47, C7orf61, TSC22D4, C7orf51, AGFG2, LRCH4, FBXO24, PCOLCE, MOSPD3, TFR2, ACTL6B, GNB2, GIGYF1, POP7, EPO, ZAN, EPHB4, SLC12A9, TRIP6, SRRT, UFSP1, ACHE, MUC3B, MUC3A, MUC12, MUC17, TRIM56, SERPINE1, AP1S1, VGF, C7orf52, MOGAT3, PLOD3, ZNHIT1, CLDN15, FIS1, RABL5, EMID2, MYL10, CUX1, SH2B2, PRKRIP1, ORAI2, ALKBH4, LRWD1, POLR2J, RASA4B, POLR2J3, SPDYE2, RASA4, POLR2J2, FAM185A, FBXL13, LRRC17, ARMC10, NAPEPLD, DPY19L2P2	hsa-mir-1285-1, hsa-mir-653, hsa-mir-489, hsa-mir-591, hsa-mir-25, hsa-mir-93, hsa-mir-106b, hsa-mir-548o	[illegible]	[illegible]
7	128594848	128873691	128:59:00	128.87	q32.1	TP0719D05	TP0704F0	3	0.278843	-0.264255811344613	4	C5, C11, C22, MPC298	TNPO3, TSPAN33, SMO, AHCYL2		[illegible]	[illegible]
7	131385701	132388700	131:39:00	132:39:00	q32.3	TP0719C07	TP0719G0	11	1,002,999	-0.566185980382243	3	C5, C9, MPC298	PLXNA4		[illegible]	[illegible]
7	140856480	148405680	140.86	148:41:00	q34-q36.1	TP0711D11	TP0706A0	78	75,492	-0.589690496511213	4	C5, C11, C17 , C18, MPC298	AGK, KIAA1147, WEE2, SSBP1, TAS2R3, TAS2R4, TAS2R5, OR9A4, CLEC5A, TAS2R38, MGAM, TRBV5-4, TRBV6-2, TRBC1, PRSS1, TRBC2, EPHB6, TRPV6, TRPV5, C7orf34, KEL, OR9A2, OR6V1, OR6W1P, PIP, TAS2R39, TAS2R40, GSTK1, TMEM139, CASP2, CLCN1, FAM131B, ZYX, EPHA1, TAS2R60, TAS2R41, FAM115C, FAM115A, OR2F2, OR2F1, OR6B1, OR2A5, OR2A25, OR2A12, OR2A2, OR2A14, ARHGEF5L, OR2A42, OR2A20P, OR2A9P, OR2A1, ARHGEF5, NOBOX, TPK1, CNTNAP2,C7orf33, CUL1	hsa-mir-548f-4	[illegible]	[illegible]
7	149547547	150563724	149:55:00	150:56:00	q36.1	TP0719C12	TP0703C0	12	1,016,177	-0.546337684796276	3	C5, C18, MPC298	ZNF862, ATP6V0E2, LRRC61, C7orf29, RARRES2, REPIN1, ZNF775, GIMAP8, GIMAP7, GIMAP4, GIMAP6, GIMAP2, GIMAP1, GIMAP5, TMEM176B, TMEM176A, ABP1		[illegible]	[illegible]
7	152630716	155434639	152.63	155:43:00	q36.2-q36.3	TP0707B04	TP0705B0	37	2,803,923	-0.523630464300857	3	C5, C9, MPC298	DPP6, PAXIP1, HTR5A, INSIG1, BLACE, EN2, CNPY1		[illegible]	[illegible]
8	47943	35647117	0:05	35.65	p23.3-p12	TP0803D02	TP0802G0	359	35,599,174	-0.728363770686917	11	C4LN, C5, C6, C9, C10, C11, C13, C18, C21, C24, MPC600, MPC298, MPC960	OR4F21, ZNF596, FAM87A, FBXO25, C8orf42, ERICH1, C8orf68, CLN8, ARHGEF10, KBTBD11, MYOM2, CSMD1, MCPH1, ANGPT2, AGPAT5, XKR5, DEFB1, DEFA6, DEFA4, DEFA1, DEFT1P, DEFA3, DEFA5, FAM90A3, FAM90A13, FAM90A5, FAM90A20, DEFB108P2, DEFB103A, SPAG11B, DEFB104B, DEFB106B, DEFB105B, DEFB107B, FAM90A23, FAM90A22, FAM90A15, FAM90A10, FAM90A8, FAM90A16, FAM90A9, DEFB107A, DEFB105A, DEFB106A, DEFB104A, SPAG11A, DEFB103B, DEFB4, DEFB108P1, FAM66E, FAM90A11, FAM90A12, MFHAS1, ERI1, PPP1R3B, TNKS, MSRA, RP1L1, C8orf74, SOX7, XKR6, C8orf16, C8orf8, MTMR9, AMAC1L2, TDH, C8orf12, FAM167A, BLK, C8orf14, GATA4, C8orf49, NEIL2, FDFT1, CTSB,DEFB134, ZNF705D, AM86B1, DEFB130, ZNF705F, FAM66A, FAM86C, OR7E8P, LONRF1, DLC1, C8orf48, SGCZ, TUSC3, MSR1, FGF20, EFHA2, ZDHHC2, CNOT7, VPS37A, MTMR7, SLC7A2, PDGFRL, MTUS1, FGL1, PCM1, ASAH1, NAT1, NAT2, PSD3, SH2D4A, CSGALNACT1, INTS10, LPL, SLC18A1, ATP6V1B2, LZTS1, GFRA2, DOK2, XPO7, NPM2, FGF17, EPB49, FAM160B2, NUDT18, HR, REEP4, LGI3, SFTPC, BMP1, PHYHIP, POLR3D, PIWIL2, SLC39A14, PPP3CC, SORBS3, PDLIM2, KIAA1967, BIN3, EGR3, RHOBTB2, TNFRSF10B, TNFRSF10C, TNFRSF10D, TNFRSF10A, CHMP7, LOXL2, ENTPD4, SLC25A37, NKX3-1, STC1, ADAM28, ADAMDEC1,	hsa-mir-596, hsa-mir-548i-3, hsa-mir-597, hsa-mir-124-1, hsa-mir-598, hsa-mir-383, hsa-mir-320a, hsa-mir-548h-4	[illegible]	[illegible]
9	1578285	6508614	1:58	6:51	p24.3-p24.1	TP0906C12	TP0904D0	44	4,930,329	-0.313733769739529	3	C4, C18, MPC960	SMARCA2, VLDLR, KCNV2, KIAA0020, CARM1L, RFX3, GLIS3, SLC1A1, C9orf68, PPAPDC2, CDC37L1, AK3, RCL1, JAK2, INSL6, INSL4, RLN2, RLN1, C9orf46, CD274, PDCD1LG2, KIAA1432, ERMP1, KIAA2026, MLANA, RANBP6, IL33, TPD52L3, UHRF2	hsa-mir-101-2	[illegible]	[illegible]
9	6986235	8064098	0.31875	8:06	p24.1	TP0911G05	TP0906H0	10	1,077,863	-0.30461830876486	3	C4, C18, MPC960	KDM4C, C9orf123		[illegible]	[illegible]
9	39131199	71087969	39:13:00	71:09:00	p11.1-q21.11	TP0904G01	TP0909B0	70	3,195,677	-0.688931341574396	9	C4, C10, C11, C13, C17 , C18, C22, C24, MPC960	CNTNAP3, FAM75A1, ZNF658B, FAM75A2, FAM74A1,FAM75A3, FAM74A3, ZNF658, FAM74A2, FAM75A4, FAM75A5, ANKRD20A2, CBWD7, FOXD4L2, ANKRD20A1, FAM75A6, CNTNAP3B, FAM27C,FAM27A, FAM27E2, FAM27E1, FAM27D1, FAM75A7, FAM27B, ANKRD20A3, FOXD4L6, CBWD6, ANKRD20A4, CCDC29, FOXD4L5, FOXD4L4, CBWD5, CBWD3, FOXD4L3, PGM5	hsa-mir-1299	[illegible]	[illegible]
9	74547890	75025333	74:55:00	75:03:00	q21.13	TP0905G03	TP0904B0	5	0.477443	-0.233983429947895	3	C4, C9, MPC960	C9orf85, C9orf57, GDA, ZFAND5		[illegible]	[illegible]
9	77732575	88076059	77.73	88:08:00	q21.13-q21.33	TP0902G12	TP0904G0	113	10,343,484	-0.302611435255011	4	C4, C21, C22, MPC960	OSTF1, PCSK5, RFK, GCNT1, PRUNE2, FOXB2, VPS13A, GNA14, GNAQ, CEP78, PSAT1, CHCHD9, TLE4, TLE1, FAM75B, RASEF, FRMD3, C9orf103, UBQLN1, GKAP1, KIF27, C9orf64, HNRNPK, RMI1, SLC28A3, NTRK2	hsa-mir-7-1	[illegible]	[illegible]
9	88848388	118010849	88.85	118:01:00	q21.33-q33.1	TP0910C02	TP0906G0	304	29,162,461	-0.584108231057697	4	C4, C4LN, C21, MPC960	C9orf153, ISCA1, ZCCHC6, GAS1, C9orf170, DAPK1, CTSL1, C9orf79, CCRK, SPIN1, NXNL2, C9orf47, S1PR3, SHC3, C9orf33, CKS2, SECISBP2, SEMA4D, GADD45G, DIRAS2, SYK, AUH, NFIL3, ROR2, SPTLC1, IARS, NOL8, CENPP, OGN, OMD, ASPN, ECM2, IPPK, BICD2, ANKRD19, ZNF484, FGD3, SUSD3, C9orf89, NINJ1, WNK2, C9orf129, FAM120AOS, FAM120A, PHF2, BARX1, PTPDC1, ZNF169, FAM22F, HIATL1, FBP2, FBP1, C9orf118, C9orf3, FANCC, PTCH1, C9orf102, HSD17B3, SLC35D2, ZNF367, HABP4, CDC14B, C9orf21, ZNF510, HIATL2, FAM22G, CTSL2, ZNF322B, KIAA1529, TDRD7, TMOD1, C9orf97, NCBP1, XPA, FOXE1, C9orf156, HEMGN, ANP32B, NANS, TRIM14, CORO2A, TBC1D2, GABBR2, ANKS6, GALNT12, COL15A1, TGFBR1, ALG2, SEC61B, KRT8P11, NR4A3, STX17, ERP44, INVS, TEX10, C9orf30, BAAT, MRPL50, ZNF189, ALDOB, C9orf125, RNF20, GRIN3A, PPP3R2, C9orf107, CYLC2, SMC2, OR13F1, OR13C4, OR13C3, OR13C8, OR13C5, OR13C2, OR13C9, OR13D1, NIPSNAP3A, NIPSNAP3B, ABCA1, SLC44A1, FSD1L, FKTN, TAL2, TMEM38B, ZNF462, RAD23B, KLF4, ACTL7B, ACTL7A, IKBKAP, C9orf6, CTNNAL1, C9orf5, C9orf4, EPB41L4B, PTPN3, AKAP2, C9orf152, TXN, TXNDC8, SVEP1, MUSK, LPAR1, OR2K2, KIAA0368, ZNF483, PTGR1, C9orf29, DNAJC25, C9orf84, UGCG, SUSD1, ROD1, HSDL2, C9orf147, KIAA1958, C9orf80, SNX30, SLC46A2, ZFP37, SLC31A2,	hsa-let-7a-1, hsa-let-7f-1, hsa-let-7d, hsa-mir-23b, hsa-mir-27b, hsa-mir-24-1, hsa-mir-1302-8, hsa-mir-32, hsa-mir-455	[illegible]	[illegible]
9	118285259	123018773	118:29:00	123:02:00	q33.1-q33.2	TP0909C12	TP0907B0	54	4,733,514	-0.368647647482156	4	C4, C21, C22, MPC960	PAPPA, ASTN2, TRIM32, TLR4, DBC1	hsa-mir-147	[illegible]	[illegible]
9	129893441	132834685	129.89	132.83	q33.3-q34.11	TP0909A07	TP0911F1	33	2,941,244	-0.324886001519644	3	C6, C21, MPC960	RALGPS1, GARNL3, SLC2A8, ZNF79, RPL12, LRSAM1, FAM129B, STXBP1, TTC16, C9orf117, TOR2A, SH2D3C, CDK9, FPGS, ENG, AK1, ST6GALNAC6, ST6GALNAC4, PIP5KL1, DPM2, FAM102A, NAIF1, SLC25A25, PTGES2, LCN2, C9orf16, CIZ1, DNM1, GOLGA2, C9orf119, TRUB2, COQ4, SLC27A4, TMSL4, URM1, CERCAM, ODF2, GLE1, SPTAN1, WDR34, SET, PKN3, ZDHHC12, ZER1, TBC1D13, ENDOG, C9orf114, CCBL1, LRRC8A, PHYHD1, DOLK, NUP188, SH3GLB2, FAM73B, DOLPP1, CRAT, PPP2R4, IER5L, METTL11A, C9orf50, ASB6, PRRX2, PTGES, TOR1B, TOR1A, C9orf78, USP20, FNBP1, GPR107	hsa-mir-199b, hsa-mir-219-2	[illegible]	[illegible]
9	134356547	138809014	134:36:00	138.81	q34.13-q34.3	TP0913A05	TP0903A0	47	4,452,467	-0.370608348504124	4	C6, C10, C21, MPC960	BAT2L, POMT1, UCK1, RAPGEF1, MED27, NTNG2, SETX, TTF1, C9orf171, BARHL1, DDX31, GTF3C4, C9orf98, C9orf9, TSC1, GFI1B, GTF3C5, CEL, RALGDS, GBGT1, OBP2B, ABO, SURF6, MED22, RPL7A, SURF1, SURF2, SURF4, C9orf96, REXO4, ADAMTS13, C9orf7, SLC2A6, ADAMTSL2, FAM163B, DBH, SARDH, VAV2, BRD3, WDR5, RXRA, COL5A1, C9orf104, FCN2, FCN1, OLFM1, C9orf62, KIAA0649, C9orf116, MRPS2, LCN1, OBP2A, PAEP, GLT6D1, LCN9, SOHLH1, KCNT1, CAMSAP1		[illegible]	[illegible]
10	38442818	43282670	38:44:00	43:28:00	p11.1-q11.21	TP1011E09	TP1011E1	12	4,839,852	-0.389639741586082	7	C10, C11, C13, C17 , C18, C22, C24	HSD17B7P2, CCNYL2, ZNF33B, BMS1		[illegible]	[illegible]
10	46154423	48278407	46:15:00	48:28:00	q11.22	TP1014F09	TP1013F0	23	2,123,984	-0.364791817889089	3	C13, C17 , C18	ANUBL1, FAM21C, AGAP4, PTPN20A, FRMPD2L2, FAM35B, SYT15, GPRIN2, ANXA8L1, PPYR1, FAM25B, BMS1P2, CTSLL7, FAM25HP,FAM21B, ASAH2C, BMS1P6, FAM25G, ANXA8		[illegible]	[illegible]
10	48482920	49656551	48:48:00	49.66	q11.22	TP1006B12	TP1011E1	11	1,173,631	-0.40886466882901	3	C13, C17 , C18	PTPN20B, FRMPD2L1, CTGLF9P, FAM25C, BMS1P7, FRMPD2, MAPK8, ARHGAP22		[illegible]	[illegible]
10	77233029	80676967	77:23:00	80.68	q22.2-q22.3	TP1001A09	TP1002A0	37	3,443,938	-0.288705955535715	5	C4, C6, C11, C13, C17 , C18	C10orf11, KCNMA1, DLG5, POLR3A, RPS24	hsa-mir-606	[illegible]	[illegible]
10	87355729	87863547	87:36:00	87.86	q23.1	TP1010F08	TP1003E0	3	0.507818	-0.245854648577534	3	C4LN, C17 , C18	GRID1		[illegible]	[illegible]
10	106092441	108402383	106:09:00	108:04:00	q25.1	TP1008H06	TP1008D0	23	2,309,942	-0.333479863238431	7	C4, C11, C13, C17 , C18, C22, C24	ITPRIP, CCDC147, SORCS3, SORCS1		[illegible]	[illegible]
10	109725582	111965589	109.73	111.97	q25.1-q25.2	TP1007A06	TP1002F0	18	2,240,007	-0.283983301167086	5	C4, C11, C13, C18, C24	XPNPEP1, ADD3		[illegible]	[illegible]
10	125070763	127159234	125:07:00	127:16:00	q26.13	TP1013F02	TP1001E0	19	2,088,471	-0.363035088222611	3	C4, C6, C18	GPR26, CPXM2, CHST15, OAT, FAM53B, METTL10, FAM175B, ZRANB1, CTBP2		[illegible]	[illegible]
11	3197300	3800429	3:02	3:08	p15.4	TP1107C03	TP1112H0	7	0.603129	-0.335002066664507	3	C18, C21, MPC600	MRGPRG, MRGPRE, ZNF195, ART5, ART1, CHRNA10, NUP98		[illegible]	[illegible]
11	3974832	8766484	0.19236111	0.38680556	p15.4	TP1110A06	TP1109H0	46	4,791,652	-0.471070655631733	5	C10, C11, C17 , C18, C21	STIM1, RRM1, OR52B4, TRIM21, OR52K2, OR52K3P, OR52K1, OR52M1, C11orf40, OR52I2, OR52I1, TRIM68, OR51D1, OR51E1, OR51E2, OR51C1P, MMP26, OR51F1, OR52R1, OR51F2, OR51S1, OR51H1P, OR51T1, OR51A6P, OR51A7, OR51G2, OR51G1, OR51A4, OR51A2, OR51L1, OR52J3, OR52E2, OR52A4, OR52A5, OR52A1, OR51V1, HBB, HBG1, HBG2, HBE1, OR51B4, OR51B5, OR51B2, OR51B6, OR51M1, OR51J1, OR51Q1, OR51I1, OR51I2, OR52D1, UBQLN3, UBQLNL, OR52H1, OR52B6, TRIM34, TRIM5, TRIM22, OR56B1, OR52N4, OR52N5, OR52N1, OR52N2, OR52E6, OR52E8, OR52E4, OR52E5, OR56A3, OR52L1, OR56A4, OR56A1, OR52L2P, OR56B4, OR52W1, C11orf42, FAM160A2, CNGA4, CCKBR, PRKCDBP, SMPD1, APBB1, HPX, TRIM3, ARFIP2, FXC1, DNHD1, RRP8, ILK, TAF10, TPP1, DCHS1, MRPL17, OR2AG2, OR2AG1, OR6A2, OR10A5, OR10A2, OR10A4, OR2D2, OR2D3, ZNF215, ZNF214, NLRP14, RBMXL2, SYT9, OLFML1, PPFIBP2, CYB5R2, OVCH2, OR10AB1P, OR5P2, OR5P3, OR10A6, OR10A3, NLRP10, EIF3F, TUB, RIC3, LMO1, STK33, TRIM66, RPL27A, ST5	hsa-mir-302e	[illegible]	[illegible]
11	10220418	15983078	10:22	0.69305556	p15.4-p15.2	TP1113B12	TP1101C0	61	576,266	-0.452046262040821	3	C10, C18, C21	SBF2, ADM, AMPD3, RNF141, LYVE1, MRVI1, CTR9, EIF4G2, ZBED5, GALNTL4, USP47, DKK3, MICAL2, MICALCL, PARVA, TEAD1, RASSF10, ARNTL, BTBD10, PTH, FAR1, SPON1, RRAS2, COPB1, PSMA1, PDE3B, CYP2R1, CALCB, CALCA, INSC		[illegible]	[illegible]
11	36397807	37966735	36:04:00	37.97	p13-p12	TP1106G01	TP1111D0	17	1,568,928	-0.332596586349698	4	C10, C11, C18, C24	TRAF6, RAG1, RAG2, C11orf74		[illegible]	[illegible]
11	42108542	43263138	42:11:00	43:26:00	p12	TP1110E03	TP1116A0	17	1,154,596	-0.299671451969172	4	C10, C11, C18, C24			[illegible]	[illegible]
11	43711970	45729837	43.71	45.73	p11.2	TP1115A05	TP1104E0	20	2,017,867	-0.39774345440204	3	C8, C10, C18	HSD17B12, ALKBH3, ACCSL, ACCS, EXT2, ALX4, CD82, TSPAN18, TP53I11, PRDM11, SYT13, CHST1		[illegible]	[illegible]

11	48449190	50069997	48:45:00	50:07:00	p11.2-p11.12	TP1112D12	TP1116B0	16	1,620,807	-0.329725285152273	4	C4LN, C10, C13, C17 , C22	OR4A4P, UBTFL7, FOLH1, TYRL, OR4C13, OR4C12		[illegible]	[illegible]
11	60753062	61122636	60.75	61:12:00	q12.2	TP1102C11	TP1109E0	6	0.369574	-0.533778002064088	3	C9, C18, MPC600, MPC298	CD6, CD5, VPS37C, PGA3, PGA5, VWCE, DDB1, DAK, CYBASC3		[illegible]	[illegible]
11	78357434	109959084	78:36:00	109.96	q14.1-q22.3	TP1107B06	TP1102C0	318	3,160,165	-0.567389485127687	7	C5, C9, C10, C11, C13, C17 , C18, C22, MPC600, MPC298	ODZ4, RPS28, FAM181B, PRCP, C11orf82, RAB30, PCF11, ANKRD42, CCDC90B, DLG2, TMEM126B, TMEM126A, CREBZF, CCDC89, SYTL2, CCDC83, PICALM, EED, C11orf73, CCDC81, ME3, PRSS23, FZD4, TMEM135, RAB38, CTSC, GRM5, TYR, NOX4, FOLH1B, TRIM77, UBTFL2, TRIM49, TRIM64, TRIM53, UBTFL1, NAALAD2, CHORDC1, FAT3, MTNR1B, SLC36A4, CCDC67, C11orf75, KIAA1731, TAF1D, C11orf54, MED17, C11orf90, HEPHL1, PANX1, FOLR4, GPR83, MRE11A, ANKRD49, FUT4, PIWIL4, AMOTL1, CWC15, KDM4D, SFRS2B, ENDOD1, SESN3, FAM76B, CEP57, MTMR2, MAML2, CCDC82, JRKL, CNTN5, TMEM133, PGR, TRPC6, ANGPTL5, KIAA1377, C11orf70, YAP1, BIRC3, BIRC2, TMEM123, MMP7, MMP20, MMP27, MMP8, MMP10, MMP1, MMP3, MMP12, MMP13, DCUN1D5, DYNC2H1, PDGFD, DDI1, CASP12, CASP4, CASP5, CASP1, CARD16, CARD18, GRIA4, KIAA1826, KBTBD3, AASDHPPT, GUCY1A2, CWF19L2, ALKBH8, ELMOD1, SLN, SLC35F2, RAB39, CUL5, ACAT1, NPAT, ATM, C11orf65, KDELC2, EXPH5, DDX10, C11orf87	hsa-mir-708, hsa-mir-1261, hsa-mir-1304, hsa-mir-548l	[illegible]	[illegible]
11	111007830	111506625	111:01:00	111:51:00	q23.1	TP1104F04	TP1107F0	3	0.498795	-0.454410614595764	3	C5, C22, MPC298	C11orf53, C11orf92, POU2AF1, BTG4, C11orf88, LAYN, SIK2	hsa-mir-34b, hsa-mir-34c	[illegible]	[illegible]
11	112033622	134927296	112:03:00	134.93	q23.1-q25	TP1110F05	TP1115G0	239	22,893,674	-0.5583562860281	9	C5, C9, C10, C13, C17 , C18, C22, MPC600, MPC298	IL18, TEX12, BCO2, PTS, C11orf34, NCAM1, TTC12, ANKK1, DRD2, TMPRSS5, ZW10, USP28, HTR3B, HTR3A, ZBTB16, NNMT, C11orf71, REXO2, FAM55A, FAM55D, FAM55B, CADM1, BUD13, ZNF259, APOA5, APOA4, APOC3, APOA1, PAFAH1B2, SIDT2, TAGLN, PCSK7, RNF214, BACE1, CEP164, DSCAML1, FXYD2, FXYD6, TMPRSS13, IL10RA, TMPRSS4, SCN4B, SCN2B, AMICA1, MPZL3, MPZL2, CD3E, CD3D, CD3G, UBE4A, ATP5L, MLL, TTC36, TMEM25, C11orf60, ARCN1, PHLDB1, TREH, DDX6, CXCR5, BCL9L, UPK2, FOXR1, CCDC84, RPS25, TRAPPC4, SLC37A4, HYOU1, VPS11, HMBS, H2AFX, DPAGT1, C2CD2L, HINFP, ABCG4, NLRX1, PDZD3, CCDC153, CBL, MCAM, RNF26, C1QTNF5, MFRP, USP2, THY1,PVRL1, TRIM29, OAF, POU2F3, TMEM136, ARHGEF12, GRIK4, TBCEL, TECTA, SC5DL, SORL1, BLID, UBASH3B, CRTAM, C11orf63, BSX, HSPA8, GRAMD1B, SCN3B, ZNF202, OR6X1, OR6M1, TMEM225, OR8D4, OR4D5, OR6T1, OR10S1, OR10G6, OR10G8, OR10G9, OR10G7, OR10D4P, VWA5A, OR10D3P, OR8F1P, OR8G1, OR8D1, OR8D2, OR8B2, OR8B3, OR8B4, OR8B8, OR8B12, OR8A1, PANX3, TBRG1, SIAE, SPA17, NRGN, VSIG2, ESAM, C11orf61, ROBO3, ROBO4, HEPN1, HEPACAM, CCDC15, SLC37A2, TMEM218, PKNOX2, FEZ1, EI24, STT3A, CHEK1, ACRV1, PATE1, PATE2, PATE3, PATE4, HYLS1, PUS3, DDX25, CDON, RPUSD4, FAM118B, SRPR, FOXRED1, TIRAP,	hsa-mir-125b-1, hsa-let-7a-2, hsa-mir-100	[illegible]	[illegible]
12	108204105	108956476	108:02:00	108.96	q23.3	TP1205A08	TP1208C1	9	0.752371	-0.359492623536723	4	C5, C9, C18, C21	WSCD2, CMKLR1, FICD, SART3, ISCU		[illegible]	[illegible]
12	114136517	115400526	114:14:00	115:04:00	q24.13-q24.21	TP1210D05	TP1214C1	16	1,264,009	-0.34446958680182	3	C5, C9, C21	RBM19, TBX5, TBX3		[illegible]	[illegible]
12	125669423	127942345	125.67	127.94	q24.31-q24.32	TP1208F05	TP1201C1	26	2,272,922	-0.325441640782529	3	C5, C18, C21	TMEM132B		[illegible]	[illegible]
13	41647101	51052239	41.65	51:05:00	q14.11-q14.3	TP1308B01	TP1312D0	109	9,405,138	-0.506458434177139	3	C5, MPC298, MPC960	WBP4, KBTBD6, KBTBD7, MTRF1, NARG1L, C13orf15, KIAA0564, DGKH, AKAP11, TNFSF11, C13orf30, EPSTI1, DNAJC15, ENOX1,CCDC122, C13orf31,SERP2, TSC22D1, NUFIP1, KIAA1704, GTF2F2, KCTD4, TPT1, SLC25A30, COG3, SPERT, SIAH3, ZC3H13, CPB2, LCP1, LRRC63, C13orf18, LRCH1, ESD, HTR2A, SUCLA2, NUDT15, MED4, ITM2B, RB1, P2RY5, RCBTB2, CYSLTR2, FNDC3A, MLNR, CDADC1, CAB39L, SETDB2, PHF11, RCBTB1, ARL11, EBPL, KPNA3, C13orf1, TRIM13, KCNRG, DLEU1	hsa-mir-16-1, hsa-mir-15a	[illegible]	[illegible]
13	92696925	114297005	92:07:00	114:03:00	q31.3-q34	TP1308D11	TP1310F0	219	2,160,008	-0.560162820622276	5	C4LN, C5, C8, C13, C21, MPC298	GPC5, GPC6, DCT, TGDS, GPR180, SOX21, ABCC4, CLDN10, DZIP1, DNAJC3, UGCGL2, HS6ST3, OXGR1, MBNL2, RAP2A, IPO5, FARP1, RNF113B, STK24, SLC15A1, DOCK9, UBAC2, GPR18, GPR183, TM9SF2, CLYBL, ZIC5, ZIC2, PCCA, A2LD1, TMTC4, NALCN, ITGBL1, FGF14, TPP2, C13orf39, C13orf27, KDELC1, BIVM, ERCC5, SLC10A2, DAOA, EFNB2, ARGLU1, FAM155A, LIG4, ABHD13, TNFSF13B, MYO16, IRS2, COL4A1, COL4A2, RAB20, CARKD, CARS2, ING1, C13orf29, ANKRD10, ARHGEF7, C13orf16, SOX1, C13orf28, TUBGCP3, C13orf35, ATP11A, MCF2L, F7, F10, PROZ, PCID2, CUL4A, LAMP1, GRTP1, ADPRHL1, DCUN1D2, TMCO3, TFDP1	hsa-mir-623, hsa-mir-1267	[illegible]	[illegible]
14	56581135	61121896	56:58:00	61:12:00	q22.3-q23.1	TP1405B03	TP1404A0	40	4,540,761	-0.46248005685578	3	C4, C8, MPC298	PELI2, C14orf101, OTX2, EXOC5, MUDENG, NAT12, C14orf105, SLC35F4, C14orf37, ACTR10, PSMA3, ARID4A, TOMM20L, TIMM9, KIAA0586, DACT1, DAAM1, GPR135, C14orf149, C14orf100, C14orf38, RTN1, LRRC9, C14orf135, DHRS7, PPM1A, C14orf39, SIX6, SIX1		[illegible]	[illegible]
14	61854263	107246329	61.85	107:25:00	q23.1-q32.33	TP1405H12	TP1410D1	443	45,392,066	-0.653779701399081	6	C4, C4LN, C5, C9, C13, C18, MPC600, MPC298	PRKCH, HIF1A, SNAPC1, SYT16, KCNH5, RHOJ, GPHB5, PPP2R5E, RPL31P5, WDR89, SGPP1, SYNE2, ESR2, MTHFD1, AKAP5, ZBTB25, ZBTB1, HSPA2, C14orf50, PLEKHG3, SPTB, FNTB, GPX2, RAB15, MAX, RPL21P7,FUT8, GPHN, FAM71D, MPP5, ATP6V1D, EIF2S1, PLEK2, C14orf83, PLEKHH1, PIGH, ARG2, VTI1B, RDH11, RDH12, ZFYVE26, RAD51L1, ZFP36L1, C14orf181, ACTN1, WDR22, EXD2, GALNTL1, ERH, SLC39A9, C14orf162, KIAA0247, SFRS5, SLC10A1, SMOC1, SLC8A3, COX16, SYNJ2BP, ADAM21, ADAM20, MED6, TTC9, MAP3K9, PCNX, C14orf56, SIPA1L1, RGS6, C14orf57, DPF3, WDR21A, ZFYVE1, RBM25, PSEN1, PAPLN, NUMB, HEATR4, ACOT1, ACOT2, ACOT4, ACOT6, DNAL1, PNMA1, C14orf43, PTGR2, ZNF410, FAM161B, COQ6, ENTPD5, C14orf45, ALDH6A1, LIN52, VSX2, ABCD4, C14orf115, TMEM90A, NPC2, ISCA2, LTBP2, KIAA0317, FCF1, YLPM1, PROX2, DLST, RPS6KL1, PGF, EIF2B2, MLH3, ACYP1, FAM164C, NEK9, TMED10, FOS, JDP2, BATF, FLVCR2, C14orf1, TTLL5, TGFB3, C14orf179, C14orf118, ESRRB, VASH1, ANGEL1, C14orf166B, C14orf4, KIAA1737, ZDHHC22, TMEM63C, NGB, POMT2, GSTZ1, TMED8, C14orf174, C14orf148, C14orf133, AHSA1, ISM2, SPTLC2, ALKBH1, C14orf156, SNW1, C14orf178, ADCK1, NRXN3, C14orf145, TSHR, GTF2A1, STON2, SEL1L, FLRT2, GALC, GPR65, KCNK10, SPATA7, PTPN21, ZC3H14, EML5, TTC8, FOXN3, C14orf143, TDP1, KCNK13, PSMC1, C14orf102, CALM1, TTC7B, RPS6KA5, C14orf159, GPR68, CCDC88C, SMEK1, CATSPERB, TC2N, FBLN5, TRIP11, ATXN3, NDUFB1, CPSF2, SLC24A4, RIN3, LGMN, GOLGA5, CHGA, ITPK1, MOAP1, C14orf109, C14orf142, UBR7, BTBD7, KIAA1409, COX8C, PRIMA1, FAM181A, ASB2, C14orf48, OTUB2, DDX24, IFI27L1, IFI27, IFI27L2,	hsa-mir-548h-1, hsa-mir-625, hsa-mir-1260, hsa-mir-342, hsa-mir-345, hsa-mir-770, hsa-mir-493, hsa-mir-337, hsa-mir-665, hsa-mir-431, hsa-mir-433, hsa-mir-127, hsa-mir-432, hsa-mir-136, hsa-mir-370, hsa-mir-379, hsa-mir-411, hsa-mir-299, hsa-mir-380, hsa-mir-1197, hsa-mir-323, hsa-mir-758, hsa-mir-329-1, hsa-mir-329-2, hsa-mir-494, hsa-mir-543, hsa-mir-495, hsa-mir-376c, hsa-mir-654, hsa-mir-300, hsa-mir-1185-1, hsa-mir-1185-2, hsa-mir-381, hsa-mir-487b, hsa-mir-539, hsa-mir-889, hsa-mir-544, hsa-mir-655, hsa-mir-487a, hsa-mir-382, hsa-mir-134, hsa-mir-668, hsa-mir-485, hsa-mir-453, hsa-mir-154, hsa-mir-496, hsa-mir-377, hsa-mir-541, hsa-mir-409, hsa-mir-412, hsa-mir-369, hsa-mir-410, hsa-mir-656, hsa-mir-1247, hsa-mir-203	[illegible]	[illegible]
15	20013225	31472757	20:01	31:47:00	q11.2-q13.3	TP0001C01	TP1504G1	150	11,459,532	-0.475586943065276	6	C8, C11, C13, C18, C21, MPC298, MPC960	VSIG7, BCL8, OR11K1P, OR4M2, OR4N4, VSIG6, TUBGCP5, CYFIP1, NIPA2, NIPA1, WHAMML1, GOLGA9P, HERC2P2, GOLGA8E, MKRN3, NDN, C15orf2, SNRPN, SNURF, C15orf49, UBE3A, ATP10A, GABRB3, GABRG3, OCA2, HERC2, GOLGA8F, GOLGA8G, HERC2P2, APBA2, NDNL2, TJP1, FAM7A3, CHRFAM7A, FAM7A2, ARHGAP11B, MTMR15, MTMR10, TRPM1	hsa-mir-1268, hsa-mir-211	[illegible]	[illegible]
15	32466659	40549368	32:47:00	40:55:00	q13.3-q15.1	TP1510C10	TP1502B0	98	8,082,709	-0.539054576864788	4	C8, C18, C21, MPC600	FAM7A1, ARHGAP11A, SCG5, GREM1, FMN1, TMCO5B, RYR3, AVEN, CHRM5, C15orf24, PGBD4, C15orf29, TMEM85, SLC12A6, NOP10, C15orf55, LPCAT4, GOLGA8A, GOLGA8B, GJD2, ACTC1, AQR, ZNF770, ATPBD4, C15orf41, MEIS2, TMCO5A, SPRED1, FAM98B, RASGRP1, C15orf53, C15orf54, THBS1, FSIP1, GPR176, EIF2AK4, SRP14, BMF, BUB1B, PAK6, C15orf56	hsa-mir-1233, hsa-mir-1233	[illegible]	[illegible]
15	42386370	42977332	42:39:00	42.98	q15.1-q15.2	TP1504B09	TP1510C1	7	0.590962	-0.348540104260268	3	C8, C21, MPC600	PLA2G4D, PLA2G4F, VPS39, TMEM87A, GANC, CAPN3, ZFP106, SNAP23, LRRC57, HAUS2, STARD9	hsa-mir-627	[illegible]	[illegible]
16	20378076	20682254	20:38	0.88055556	p12.3	TP1609B03	TP1608H0	4	0.304178	-0.343901038616275	3	C4, C9, C11	PDILT, ACSM5, ACSM2A, ACSM2B, ACSM1		[illegible]	[illegible]
16	32062731	33784671	32:06:00	33.78	p11.2-p11.1	TP1611F01	TP1608H1	15	172,194	-0.568274252098773	5	C10, C18, C21, C22, C24	IGHV2OR16-5, TP53TG3		[illegible]	[illegible]
16	47387507	52620069	47:39:00	52.62	q12.1-q12.2	TP1607C07	TP1607D1	67	5,232,562	-0.39941625921642	6	C4, C6, C9, C13, C21, C22	ITFG1, PHKB, ABCC12, ABCC11, LONP2, SIAH1, N4BP1, CBLN1, C16orf78, ZNF423, TMEM188, HEATR3, PAPD5, ADCY7, BRD7, NKD1, SNX20, NOD2, CYLD, SALL1, TOX3		[illegible]	[illegible]
16	53753107	89051718	53.75	89:05:00	q12.2-q24.3	TP1605F08	TP1610E0	476	35,298,611	-0.523103335964998	10	C4, C4LN, C5, C6, C8, C9, C10, C13, C17 , C21, C22, MPC600	FTO, IRX3, IRX5, IRX6, MMP2, LPCAT2, SLC6A2, CES4, CES1, CES7, GNAO1, AMFR, NUDT21, OGFOD1, BBS2, MT4, MT3, MT2A, MT1E, , MT1M, MT1A, MT1DP, MT1CP, MT1B, MT1F, MT1G, MT1H, MT1X, NUP93, SLC12A3, HERPUD1, CETP, NLRC5, CPNE2, RSPRY1, ARL2BP, PLLP, CCL22, CX3CL1, CCL17, CIAPIN1, COQ9, POLR2C, DOK4, CCDC102A, GPR114, GPR56, GPR97, CCDC135, KATNB1, KIFC3, CNGB1, TEPP, ZNF319, C16orf57, MMP15, C16orf80, CSNK2A2, CCDC113, GINS3, NDRG4, SETD6, CNOT1, SLC38A7, GOT2, CDH8, CDH11, CDH5, TK2, CKLF, CMTM1, CMTM2, CMTM3, CMTM4, DYNC1LI2, CCDC79, NAE1, CA7, CDH16, RRAD, CES2, CES3, CES8, CBFB, C16orf70, B3GNT9, TRADD, FBXL8, HSF4, NOL3, EXOC3L, E2F4, ELMO3, LRRC29, TMEM208, FHOD1, SLC9A5, PLEKHG4, KCTD19, LRRC36, TPPP3, ZDHHC1, HSD11B2, ATP6V0D1, AGRP, FAM65A, CTCF, RLTPR, ACD, PARD6A, C16orf48, C16orf86, GFOD2, RANBP10, TSNAXIP1, CENPT, THAP11, NUTF2, EDC4, NRN1L, PSKH1, CTRL, PSMB10, LCAT, SLC12A4, DPEP3, DPEP2, DDX28, DUS2L, NFATC3, ESRP2, PLA2G15, SLC7A6, SLC7A6OS, PRMT7, SMPD3, ZFP90, CDH3, CDH1, TMCO7, HAS3, CHTF8, CIRH1A, SNTB2, VPS4A, PDF, COG8, NIP7, TMED6, TERF2, CYB5B, NFAT5, NQO1, NOB1, WWP2, CLEC18A, PDXDC2, PDPR, CLEC18C, EXOSC6, AARS, DDX19B, DDX19A, ST3GAL2, FUK,	hsa-mir-138-2, hsa-mir-328, hsa-mir-1538, hsa-mir-140, hsa-mir-1972, hsa-mir-1910	[illegible]	[illegible]
17	1002	1917776	0	0.10555556	p13.3	TP1710G01	TP1706C0	33	1,916,774	-0.429471406863677	4	C5, C8, MPC600, MPC298	DOC2BL, RPH3AL, C17orf97, FAM101B, VPS53, FAM57A, GEMIN4, GLOD4, RNMTL1, NXN, TIMM22, ABR, BHLHA9, TUSC5, YWHAE, CRK, MYO1C, INPP5K, PITPNA, SLC43A2, SCARF1, RILP, PRPF8, TLCD2, WDR81, SERPINF2, SERPINF1, SMYD4, RPA1, RTN4RL1	hsa-mir-22	[illegible]	[illegible]
17	2589101	15687248	2:59	0.67291667	p13.3-p12	TP1704D07	TP1705D1	132	13,098,147	-0.463431610017653	5	C5, C8, C9, C13, MPC600, MPC298	KIAA0664, GARNL4, OR1D4, OR1D2, OR1G1, OR1A2, OR1A1, OR3A2, OR3A1, OR1E1, OR3A3, OR1E2, SPATA22, ASPA, TRPV3, , SHPK, CTNS, TAX1BP3, TMEM93, P2RX5, ITGAE, GSG2, C17orf85, CAMKK1, P2RX1, ATP2A3, ZZEF1, CYB5D2, ANKFY1, SPNS3, SPNS2, MYBBP1A, GGT6, SMTNL2, ALOX15, PELP1, ARRB2, MED11, CXCL16, ZMYND15, TM4SF5, VMO1, GLTPD2, PSMB6, PLD2, MINK1, CHRNE, GP1BA, SLC25A11, RNF167, PFN1, ENO3, SPAG7, CAMTA2, INCA1, KIF1C, GPR172B, ZFP3, ZNF232, USP6, ZNF594, C17orf87, RABEP1, NUP88, RPAIN, , C1QBP, DHX33, DERL2, MIS12, NLRP1, WSCD1, AIPL1, FAM64A, PITPNM3, KIAA0753, TXNDC17, MED31, C17orf100, SLC13A5, XAF1, FBXO39, TEKT1, ALOX12, RNASEK, C17orf49, BCL6B, SLC16A13, SLC16A11, CLEC10A, ASGR2, ASGR1, DLG4, ACADVL, DVL2, PHF23, GABARAP, DULLARD, C17orf81, CLDN7, SLC2A4, YBX2, EIF5A, GPS2, NEURL4, ACAP1, KCTD11, TMEM95, TNK1, PLSCR3, C17orf61, NLGN2, SPEM1, C17orf74, TMEM102, FGF11, CHRNB1, ZBTB4, AMAC1L3, POLR2A, TNFSF12, SENP3, EIF4A1, CD68, MPDU1, SOX15, FXR2, SAT2, SHBG, ATP1B2, TP53, WRAP53, EFNB3, DNAH2, KDM6B, TMEM88, LSMD1, CYB5D1, CHD3, KCNAB3, TRAPPC1, CNTROB, GUCY2D, ALOX15B, ALOX12B, ALOXE3, HES7, PER1, VAMP2, TMEM107, C17orf59, AURKB, C17orf68, PFAS, SLC25A35, RANGRF, ARHGEF15, ODF4,	hsa-mir-1253, hsa-mir-195, hsa-mir-497, hsa-mir-324, hsa-mir-744, hsa-mir-548h-3	[illegible]	[illegible]
17	18276146	18755715	18:28	0.80277778	p11.2	TP1704E10	TP1704D1	6	0.479569	-0.291119914574587	5	C10, C13, C18, C22, MPC600	EVPLL, LGALS9C, CCDC144B, TBC1D28, TRIM16L, FBXW10, FAM18B, PRPSAP2		[illegible]	[illegible]
17	20165776	20506788	20:17	20:51	p11.2	TP1710C03	TP1702F0	6	0.341012	-0.313581809813124	4	C10, C13, C22, MPC600	CYTSB, CCDC144C, FAM106B, CDRT15L2		[illegible]	[illegible]
17	21179534	21991358	21:18	0.94375	p11.2-p11.1	TP1709A01	TP1706D0	13	0.811824	-0.271701008103438	3	C10, C18, MPC600	MAP2K3, KCNJ12, C17orf51,FAM27L		[illegible]	[illegible]
17	22017418	25461298	22:02	25:46:00	p11.1-q11.1	TP1703H09	TP1702A0	4	344,388	-0.289971553057978	3	C10, C18, C24			[illegible]	[illegible]
17	31312257	32596575	31:31:00	32:06:00	q11.2-q12	TP1705B12	TP1702E0	15	1,284,318	-0.361465470696483	3	C9, C10, C18	SPACA3, ACCN1, CCL2		[illegible]	[illegible]
18	3783133	7070811	0.17916667	7:07	p11.31	TP1807F09	TP1807B0	32	3,287,678	-0.323103250390508	3	C21, C22, MPC600	DLGAP1, C18orf18, ZFP161, EPB41L3, L3MBTL4, ARHGAP28, LAMA1		[illegible]	[illegible]
18	7440708	8166659	7:44	8:17	p11.23	TP1805E03	TP1116G0	10	0.725951	-0.269205706336844	3	C21, C22, MPC600	PTPRM		[illegible]	[illegible]
18	13751585	15305632	0.59375	15:31	p11.21-p11.1	TP1806A09	TP1808A0	15	1,554,047	-0.348324777662306	7	C4LN, C10, C11, C13, C17 , C18, C22, C24	RNMT, MC5R, MC2R, ZNF519, NF1L5, POTEC, ANKRD30B		[illegible]	[illegible]
18	21990478	27572989	0.94375	27:57:00	q11.2-q12.1	TP1802C03	TP1803B1	61	5,582,511	-0.475465909857226	5	C4LN, C17 , C18, C22, MPC600	IMPACT, HRH4, ZNF521, SS18, PSMA8, TAF4B, KCTD1, AQP4, CHST9, CDH2		[illegible]	[illegible]
18	30254705	30646709	30:25:00	30.65	q12.1	TP1801G05	TP1806F0	3	0.392004	-0.406895870340568	3	C22, MPC600, MPC298	KLHL14, C18orf34		[illegible]	[illegible]
18	33926155	43152274	33.93	43:15:00	q12.2-q12.3	TP1805D06	TP1804C1	86	9,226,119	-0.373054752542388	6	C9, C13, C17 , C18, C22, MPC600	FHOD3, C18orf10, KIAA1328, BRUNOL4, PIK3C3, RIT2, SYT4, SETBP1		[illegible]	[illegible]
18	48678935	56446495	48.68	56:45:00	q21.2-q21.32	TP1802D02	TP1807A1	82	776,756	-0.340801874853017	4	C17 , C18, C22, MPC600	MEX3C, DCC, MBD2, POLI, STARD6, C18orf54, C18orf26, RAB27B, CCDC68, TCF4, TXNL1, WDR7, ST8SIA3, ONECUT2, FECH, NARS, ATP8B1, ALPK2, MALT1	hsa-mir-122	[illegible]	[illegible]
18	70956549	74025238	70.96	74:03:00	q22.3-q23	TP1805G05	TP1808E1	30	3,068,689	-0.390341184146749	4	C8, C18, C22, MPC600	FBXO15, C18orf55, CYB5A, C18orf51, CNDP2, CNDP1, ZNF407, C18orf33, ZADH2, TSHZ1, C18orf62		[illegible]	[illegible]
18	75115336	76176890	75:12:00	76:18:00	q23	TP1801A01	TP0914G1	10	1,061,554	-0.545942556394517	4	C17 , C18, C22, MPC600			[illegible]	[illegible]
19	6249888	10815611	6:25	0.47361111	p13.3-p13.2	TP1901G06	TP1904A0	60	4,565,723	-0.782449647199018	10	C4, C4LN, C5, C9, C13, C17 , C21, C22, MPC600, MPC960	MLLT1, ACER1, CLPP, ALKBH7, PSPN, GTF2F1, KHSRP, SLC25A41, SLC25A23, CRB3, DENND1C, TUBB4, TNFSF9, CD70, TNFSF14, C3, GPR108, TRIP10, SH2D3A, VAV1, EMR1, EMR4P,MBD3L2, ZNF557, INSR, ARHGEF18, PEX11G, C19orf45, ZNF358, MCOLN1, PNPLA6, KIAA1543, XAB2, PCP2, STXBP2, RETN, C19orf59, TRAPPC5, FCER2, CLEC4G, CD209, CLEC4M, EVI5L, LRRC8E, MAP2K7, SNAPC2, CTXN1, TIMM44, ELAVL1, CCL25, FBN3, LASS4, CD320, NDUFA7, RPS28P9, KANK3, ANGPTL4, RAB11B, Mar-02, HNRNPM, PRAM1, ZNF414, MYO1F, ADAMTS10, ACTL9, OR2Z1, ZNF558, MBD3L1, MUC16, OR1M1, OR7G2, OR7G1, OR7G3, ZNF317, OR7D2, OR7D4, OR7E24, ZNF699, ZNF559, ZNF177, ZNF266, ZNF560, ZNF426, ZNF121, ZNF561, ZNF562, ZNF846, FBXL12, UBL5, PIN1, OLFM2, COL5A3, RDH8, C3P1, C19orf66, ANGPTL6, PPAN, EIF3G, DNMT1, S1PR2, MRPL4, ICAM1, ICAM4, ICAM5, FDX1L, RAVER1, ICAM3, TYK2, CDC37, PDE4A, KEAP1, S1PR5, ATG4D, KRI1, CDKN2D, AP1M2, SLC44A2,ILF3, QTRT1	hsa-mir-220b, hsa-mir-1181, hsa-mir-1238	[illegible]	[illegible]
19	13119211	13509577	13:12	13:51	p13.2	TP1905D03	TP1905E1	4	0.390366	-0.253628612109254	3	C5, C9, MPC600	NFIX, LYL1, TRMT1, NACC1, STX10, IER2, CACNA1A		[illegible]	[illegible]
19	15532145	16176047	15:53	16:18	p13.12	TP1901E12	TP1903C0	6	0.643902	-0.199176164017033	3	C9, C22, MPC600	WIZ, RASAL3, PGLYRP2, CYP4F22, CYP4F8, CYP4F3, CYP4F12, OR10H2, OR10H3, OR10H5, OR10H1, ZNF861P, CYP4F2, CYP4F11, OR10H4	hsa-mir-1470	[illegible]	[illegible]
19	28315720	30137501	28:32:00	30:14:00	q11-q12	TP1906H12	TP1901C0	24	1,821,781	-0.373210417187873	3	C5, C17 , MPC600	UQCRFS1, VSTM2B, POP4		[illegible]	[illegible]
19	30495914	32906559	30:05:00	32.91	q12-q13.11	TP1904D05	TP1903C0	31	2,410,645	-0.306553499449653	3	C5, C17 , MPC600	C19orf2, ZNF536, TSHZ3, ZNF507, DPY19L3		[illegible]	[illegible]
19	43221207	43933334	43:22:00	43.93	q13.2-q13.31	TP1903F11	TP1901H1	6	0.712127	-0.338546562820541	5	C5, C10, C17 , C18, MPC600	PSG3, PSG8, PSG1, PSG6, PSG7, PSG5, PSG9, CD177, CD177P, TEX101		[illegible]	[illegible]
19	48293156	48738890	48:29:00	48.74	q13.33	TP1509H07	TP1901A0	5	0.445734	-0.251819656037249	4	C5, C13, C17 , MPC600	TPRX1, CRX, SULT2A1, BSPH1, ELSPBP1, CABP5, CABP3, PLA2G4C, LIG1, C19orf68, CARD8		[illegible]	[illegible]
19	54894238	55290565	54.89	55:29:00	q13.42	TP1905D11	TP0003B0	6	0.396327	-0.399313081964781	4	C5, C11, C24, MPC600	TTYH1, LENG8, LENG9, CDC42EP5, LAIR2, KIR3DX1, LILRB1, LILRA1,LILRB4, KIR2DL4, KIR2DL1		[illegible]	[illegible]
20	25907484	29603716	25.91	29:06:00	p11.1-q11.21	TP1212E11	TP2006B1	9	3,696,232	-0.314344392303772	5	C11, C13, C18, C22, C24	FAM182A, C20orf191	hsa-mir-663	[illegible]	[illegible]
21	10907345	15586283	0.47986111	15:59	q11.1-q11.2	TP0815E06	TP0125H0	15	4,678,938	-0.418029068289686	8	C10, C11, C13, C17 , C18, C22, C24, MPC600	TPTE, BAGE, POTED, LIPI		[illegible]	[illegible]
21	40946068	42691546	40.95	42.69	q22.2-q22.3	TP0420H11	TP0718H1	23	1,745,478	-0.332097897904458	4	C9, C17 , C22, MPC600	B3GALT5, C21orf88, IGSF5, PCP4, DSCAM, BACE2, FAM3B		[illegible]	[illegible]
22	16061103	17579409	16:06	17:58	q11.1	TP1410H01	TP1116E0	22	1,518,306	-0.574628473379673	8	C4LN, C8, C10, C11, C13, C17 , C18, C22, C24	POTEH, OR11H1, KCNMB3L, CCT8L2, XKR3, GAB4, IL17RA		[illegible]	[illegible]
22	17724207	50994394	0.75833333	50.99	q11.1-q13.33	TP1015H09	TP0001B0	333	33,270,187	-0.598960083452294	8	C4, C4LN, C5, C8, C9, C10, C17 , C21, MPC600	CECR1, CECR2, SLC25A18, ATP6V1E1, BCL2L13, BID, C22orf37, MICAL3, PEX26, TUBA8, USP18, DGCR6, PRODH, DGCR2, DGCR14, TSSK2, GSC2, SLC25A1, CLTCL1, HIRA, MRPL40, UFD1L, CDC45L, CLDN5, RPL7AP70, Sep-05, GP1BB, TBX1, GNB1L, C22orf29, TXNRD2, COMT, ARVCF, C22orf25, DGCR8, TRMT2A, RANBP1, ZDHHC8, RTN4R, DGCR6L, GGTLC3, TMEM191B, RIMBP3, USP41, ZNF74, KLHL22, MED15, POM121L4P, TOP3B, SERPIND1, SNAP29, CRKL, AIFM3, LZTR1, THAP7, P2RX6, SLC7A4, POM121L7, GGT2, RIMBP3B, HIC2, RIMBP3C, UBE2L3, YDJC, CCDC116, SDF2L1, PPIL2, YPEL1, MAPK1, PPM1F, IGLV4-69, IGLV8-61, IGLV4-60, IGLV6-57, IGLV11-55, , IGLV10-54, VPREB1, IGLV5-52, IGLV7-46, IGLV5-45, IGLV1-44, IGLV7-43, IGLV1-40, IGLV5-37, IGLV1-36, ZNF280B, ZNF280A, PRAME, IGLV2-33, IGL@, GGTLC2, IGLV3-27, IGLV3-25, IGLV2-23, IGLV3-22, IGLV3-21, IGLV3-19, IGLV2-18, IGLV3-16, IGLV2-14, IGLV3-12, IGLV2-11, IGLV4-3, IGLV3-1, IGL@ , RTDR1, GNAZ, RAB36, BCR, IGLL1, C22orf43, RGL4, ZNF70, VPREB3, C22orf15, CHCHD10, MMP11,SMARCB1, DERL3, SLC2A11, GSTT2B, DDTL, DDT, GSTT2, GSTT1, CABIN1, SUSD2, GGT5, CYTSA, ADORA2A, UPB1, C22orf13, SNRPD3, GGT1, C22orf36, PIWIL3, SGSM1, TMEM211, CRYBB3, CRYBB2,	hsa-mir-648, hsa-mir-185, hsa-mir-1306, hsa-mir-1286, hsa-mir-649, hsa-mir-301b, hsa-mir-130b, hsa-mir-650, hsa-mir-548j, hsa-mir-658, hsa-mir-659, hsa-mir-1281, hsa-mir-33a, hsa-mir-1249, hsa-let-7a-3, hsa-let-7b	[illegible]	[illegible]
23	2971434	48446501	0.15069444	48:45:00	p22.33-p11.23	TPX015A10	TPX002E1	435	45,475,067	-0.760911699842324	7	C5, C6, C9, C11, C13, C17 , MPC600, MPC298	ARSF, CXorf28, MXRA5,PRKX, NLGN4X, VCX3A, HDHD1A, STS, VCX, PNPLA4, VCX2, VCX3B, KAL1, FAM9A, FAM9B, TBL1X, GPR143, SHROOM2, WWC3, CLCN4, MID1, HCCS, ARHGAP6, AMELX, MSL3, FRMPD4, PRPS2, TLR7, TLR8, TMSB4X, FAM9C, ATXN3L, EGFL6, TCEANC, RAB9A, TRAPPC2, OFD1, GPM6B, GEMIN8, GLRA2, FANCB, MOSPD2, ASB9, ASB11, PIGA, FIGF, PIR, BMX, ACE2, TMEM27, CA5BP, CA5B, ZRSR2, AP1S2, GRPR, MAGEB17, CTPS2, S100G, SYAP1, CXorf15, RBBP7, REPS2, NHS, SCML1, RAI2, BEND2, SCML2, CDKL5, RS1, PPEF1, PHKA2, GPR64, PDHA1, MAP3K15, SH3KBP1, CXorf23, MAP7D2, EIF1AX, RPS6KA3, CNKSR2, KLHL34, SMPX, MBTPS2, YY2, SMS, PHEX, ZNF645, DDX53, PTCHD1, PRDX4, ACOT9, SAT1, APOO, CXorf58, KLHL15, EIF2S3, ZFX, PDK3, PCYT1B, POLA1, ARX, MAGEB18, MAGEB6, MAGEB5, MAGEB10, WDR42B, IL1RAPL1, MAGEB2, MAGEB3, MAGEB4, MAGEB1, NR0B1, CXorf21, GK, MAP3K7IP3, CXorf29, FTHL17, DMD, FAM47A, TMEM47, FAM47B, MAGEB16, CXorf22, CXorf59, CXorf30, FAM47C, PRRG1, LANCL3, XK, CYBB, DYNLT3, CXorf27, SYTL5, SRPX, RPGR, OTC, TSPAN7, MID1IP1, BCOR, ATP6AP2, CXorf38, MED14, USP9X, DDX3X, NYX, CASK, GPR34, GPR82, MAOA, MAOB, NDP, EFHC2, FUNDC1, DUSP21, KDM6A, CXorf36, ZNF673, ZNF674, CHST7, SLC9A7, RP2, CXorf31, PHF16, RGN, NDUFB11, RBM10,	hsa-mir-651, hsa-mir-548f-5, hsa-mir-221, hsa-mir-222	[illegible]	[illegible]
23	49070185	54654547	49:07:00	54.65	p11.23-p11.22	TPX001G12	TPX009A1	44	5,584,362	-0.404895475826235	6	C9, C11, C13, C17 , C24, MPC600	CACNA1F, CCDC22, FOXP3, PPP1R3F, GAGE10, GAGE12C, GAGE12J, GAGE2D, GAGE13, GAGE2C, GAGE2B, GAGE12B, GAGE12F, GAGE12D, GAGE12E, GAGE12G, GAGE12H, GAGE1, GAGE2A, PAGE1, PAGE4, CLCN5, AKAP4, CCNB3, SHROOM4, BMP15, NUDT10, CXorf67, NUDT11, GSPT2, MAGED1, MAGED4B, MAGED4, XAGE2, XAGE1B, XAGE2B, XAGE1C, XAGE1D, XAGE1E, SSX8, SSX7, SSX2B, SPANXN5, XAGE5, XAGE3, FAM156A, GPR173, TSPYL2, KDM5C, IQSEC2, SMC1A, RIBC1, HSD17B10, HUWE1, PHF8, FAM120C, WNK3, TSR2, FGD1, GNL3L	hsa-mir-532, hsa-mir-188, hsa-mir-500, hsa-mir-362, hsa-mir-501, hsa-mir-660, hsa-mir-502, hsa-mir-98, hsa-let-7f-2	[illegible]	[illegible]

23	54874167	152884368	54.87	152.88	p11.21-q28	TPX006G05	TPX001F1	940	98,010,201	-0.540360585806094	6	C6, C8, C9, C11, C13, C17 , MPC600, MPC298, MPC960	TRO, PFKFB1, APEX2, ALAS2, PAGE2B, PAGE2, FAM104B, PAGE5, PAGE3, MAGEH1, RRAGB, KLF8, UBQLN2, SPIN3, SPIN2B, SPIN2A, FAAH2, ZXDB, ZXDA, SPIN4, ARHGEF9, FAM123B, ASB12, MTMR8, ZC4H2, ZC3H12B, LAS1L, MSN, VSIG4, HEPH, EDA2R, AR, OPHN1, YIPF6, STARD8, EFNB1, PJA1, FAM155B, EDA, OTUD6A, IGBP1, DGAT2L6, AWAT1, P2RY4, ARR3, RAB41, PDZD11, KIF4A, GDPD2, DLG3, TEX11, SLC7A3, SNX12, FOXO4, CXorf65, IL2RG, MED12, NLGN3, GJB1, ZMYM3, NONO, ITGB1BP2, TAF1, OGT, ACRC, CXCR3, RPS26P11, NHSL2, PIN4, ERCC6L, RPS4X, CITED1, HDAC8, PHKA1, DMRTC1, DMRTC1B, CXorf50, PABPC1L2B, PABPC1L2A, NAP1L6, NAP1L2, CDX4, CHIC1, FXYD8, BMP2KL, ZCCHC13, SLC16A2, RLIM, KIAA2022, ABCB7, UPRT, ZDHHC15, MAGEE2, CXorf26, MAGEE1, FGF16, ATRX, MAGT1, COX7B, ATP7A, PGAM4, PGK1, TAF9B, CYSLTR1, ZCCHC5, LPAR4, P2RY10, GPR174, ITM2A, TBX22, FAM46D, BRWD3, NSBP1, SH3BGRL, POU3F4, CYLC1, RPS6KA6, HDX, APOOL, SATL1, ZNF711, POF1B, CHM, DACH2, KLHL4, CPXCR1, TGIF2LX, PABPC5, PCDH11X, NAP1L3, FAM133A, DIAPH2, RPA4, PCDH19, TNMD, TSPAN6, SRPX2, SYTL4, CSTF2, NOX1, XKRX, ARL13A, TRMT2B, TMEM35, CENPI, DRP2, TAF7L, TIMM8A, BTK, RPL36A, GLA, ARMCX1, ARMCX6, ARMCX3, ARMCX2, NXF5, ZMAT1, TCEAL2, TCEAL6, BEX5, NXF2, NXF2B, TMSB15A, ARMCX5, GPRASP1, GPRASP2, BHLHB9, RAB40AL, BEX1, NXF3, BEX4, TCEAL8, TCEAL5, BEX2, TCEAL7, WBP5, NGFRAP1, RAB40A, TCEAL4, TCEAL3, TCEAL1, MORF4L2, GLRA4, TMEM31, PLP1, RAB9B, H2BFWT, H2BFM, MCART6, CXorf39, ESX1, IL1RAPL2, TEX13A, NRK, SERPINA7, MUM1L1, CXorf57, RNF128, TBC1D8B, RIPPLY1, CLDN2, MORC4, RBM41, NUP62CL, CXorf41, MYCL2, FRMPD3, PRPS1, TSC22D3, NCBP2L, MID2, TEX13B, VSIG1, PSMD10, ATG4A, COL4A6, COL4A5, IRS4, GUCY2F, NXT2, KCNE1L, ACSL4, TMEM164, AMMECR1, GNG5P2, CHRDL1, PAK3, CAPN6, DCX, ALG13, TRPC5,	hsa-mir-1468, hsa-mir-223, hsa-mir-421, hsa-mir-374b, hsa-mir-545, hsa-mir-374a, hsa-mir-325, hsa-mir-548i-4, hsa-mir-361, hsa-mir-548m, hsa-mir-652, hsa-mir-1912, hsa-mir-1298, hsa-mir-1911, hsa-mir-448, hsa-mir-1277, hsa-mir-766, hsa-mir-220a, hsa-mir-363, hsa-mir-92a-2, hsa-mir-19b-2, hsa-mir-20b, hsa-mir-106a, hsa-mir-450b, hsa-mir-450a-1, hsa-mir-450a-2, hsa-mir-542, hsa-mir-503, hsa-mir-424, hsa-mir-934, hsa-mir-504, hsa-mir-505, hsa-mir-320d-2, hsa-mir-890, hsa-mir-888, hsa-mir-892a, hsa-mir-892b, hsa-mir-891b, hsa-mir-891a, hsa-mir-513c, hsa-mir-513b, hsa-mir-513a-1, hsa-mir-513a-2, hsa-mir-506, hsa-mir-507, hsa-mir-508, hsa-mir-509-2, hsa-mir-509-3, hsa-mir-509-2, hsa-mir-510, hsa-mir-514-1, hsa-mir-514-2, hsa-mir-514-2, hsa-mir-224, hsa-mir-452, hsa-mir-105-1, hsa-mir-767, hsa-mir-105-2	V_34417, V_34578, V_34509, V_3251, V_3266, V_3252, V_3267, V_3268, V_3253, V_3254, V_3269, V_3255, V_3256, V_3270, V_3257, V_3271, V_3272, V_3258, V_3273, V_3259, V_8307, V_8308, V_8309, V_8310, V_8913, V_8914, V_8311, V_8312, V_8313, V_8314, V_38778, V_38768, V_38930, V_38946, V_38758, V_38760, V_108005, V_108006, V_38763, V_38762, V_38782, V_38928, V_108004, V_38767, V_38761, V_38001, V_38929, V_7792, V_7793, V_7795, V_7797, V_7799, V_7800, V_7756, V_7757, V_7758, V_7759, V_7760, V_7761, V_7762, V_7763, V_7764, V_7765, V_7766, V_7767, V_7768, V_7769, V_23330, V_31552, V_31553, V_33165, V_31554, V_33166, V_33167, V_33168, V_33169, V_31555, V_33170, V_33171, V_33172, V_33173, V_33174, V_31556, V_31557, V_33175, V_31558, V_31559, V_31560, V_33176, V_31561, V_33177, V_31562, V_31563, V_33178, V_33179, V_31564, V_33180, V_31565, V_31566, V_33181, V_31567, V_31568, V_31569, V_33182, V_33183, V_31570, V_33184, V_33185, V_31571, V_31572, V_31573, V_31574, V_67980, V_67981, V_67982, V_67983, V_67984, V_67985, V_67986, V_67987, V_67988, V_67989, V_67990, V_67991, V_67992, V_67993, V_67995, V_67996, V_67994, V_67997, V_67998, V_68002, V_68000, V_68001, V_68003, V_68004, V_68005, V_68006, V_68008, V_68009, V_68010, V_68011, V_68014, V_68015, V_68016, V_68017, V_68018, V_68021, V_68023, V_68022, V_68024, V_68027, V_68028, V_68029, V_68030, V_68032, V_68033, V_68034, V_68035, V_68036, V_68037, V_68039, V_68040, V_68041, V_68042, V_68044, V_68045, V_68046, V_68047, V_68048, V_68050, V_68051, V_68052, V_68053, V_68054, V_68055, V_68056, V_68057, V_68058, V_59550, V_59551, V_59552, V_59543, V_59544, V_59545, V_4150, V_0243, V_4151, V_4152, V_2279, V_4153, V_4154, V_4155, V_0244, V_4156, V_0245, V_0246, V_0247, V_0248, V_4157, V_4158, V_0249, V_4159, V_4160, V_4161, V_0250, V_4162, V_0251, V_4163, V_4164, V_4166, V_4167, V_4168, V_0252, V_4169, V_0253, V_4170, V_4171, V_4172, V_0254, V_4173, V_2130, V_2280, V_4174, V_0826, V_2281, V_4175, V_0827, V_2282, V_0255, V_4176, V_4177, V_4178, V_83529, V_96700, V_83530, V_96701, V_83531, V_96704, V_83534, V_96705, V_96706, V_83535, V_96707, V_96708, V_96709, V_96710, V_96711, V_83537, V_83538, V_83539, V_83540, V_83542, V_96712, V_96715, V_83541, V_96716, V_96714, V_83543, V_96713, V_83544, V_83545, V_96717, V_83547, V_83548, V_83549, V_83550, V_83551, V_96721, V_96722, V_83552, V_96723, V_83553, V_96724, V_96725, V_83557, V_96726, V_83563, V_83564, V_83565, V_96730, V_83566, V_83567, V_96731, V_83569, V_96732, V_83572, V_83570, V_96733, V_96734, V_83573, V_96735, V_83576, V_96736, V_83578, V_96740, V_96741, V_83583, V_96745, V_96746, V_83584, V_96752, V_96753, V_96754, V_96755, V_96756, V_83589, V_96757, V_83596, V_96763, V_96764, V_83598, V_96765, V_96766, V_83603, V_96770, V_96773, V_83609, V_96777, V_96779, V_96780, V_83610, V_96778, V_96781, V_96782, V_96783, V_83611, V_96784, V_96785, V_96788, V_96789, V_83613, V_96790, V_83614, V_96791, V_96793, V_83615, V_96794, V_96795, V_96797, V_96799, V_96796, V_96800, V_96798, V_83616, V_83617, V_96801, V_96802, V_96803, V_96805, V_83621, V_96807, V_96809, V_83622, V_96812, V_96814, V_96815, V_83623, V_83624, V_96816, V_83625, V_96818, V_83627, V_96821, V_96822, V_83632, V_96823, V_83633, V_96824, V_83635, V_96825, V_96830, V_96831, V_96832, V_96833, V_83642, V_96839, V_96840, V_83648, V_96845, V_96846, V_83649, V_96847, V_96848, V_96850, V_96851,	V_23256, V_105896, V_107527, V_73984, V_83529, V_96700, V_83530, V_96701, V_83531, V_63100, V_44414, V_44415, V_44416, V_43647, V_62973, V_37384, V_37385, V_37047, V_107528, V_23921, V_73985, V_73986, V_38778, V_37048, V_96704, V_103252, V_53011, V_59550, V_67980, V_22863, V_38768, V_83534, V_96705, V_96706, V_4150, V_37049, V_37050, V_37051, V_7792, V_0661, V_37052, V_37053, V_83535, V_96707, V_73987, V_107529, V_31552, V_96708, V_31553, V_96709, V_96710, V_96711, V_83537, V_83538, V_1848, V_83539, V_0243, V_7793, V_73988, V_4151, V_33165, V_83540, V_67981, V_83542, V_96712, V_96715, V_37054, V_83541, V_96716, V_96714, V_83543, V_96713, V_31554, V_83544, V_53013, V_83545, V_96717, V_53014, V_115307, V_9344, V_83547, V_1849, V_83548, V_83549, V_83550, V_83551, V_96721, V_96722, V_83552, V_96723, V_83553, V_96724, V_1850, V_73990, V_73991, V_22834, V_23922, V_96725, V_73989, V_103385, V_104555, V_22884, V_23923, V_23924, V_107977, V_63102, V_63101, V_59880, V_62974, V_9345, V_53015, V_83557, V_96726, V_4152, V_67982, V_2279, V_67983, V_38930, V_7795, V_73993, V_73994, V_73995, V_37055, V_3251, V_4153, V_83563, V_9346, V_73996, V_38946, V_107978, V_73997, V_1851, V_107981, V_73998, V_103253, V_44417, V_105897, V_53017, V_34417, V_115309, V_67984, V_83564, V_83565, V_96730, V_115310, V_83566, V_1852, V_4154, V_3266, V_53019, V_33166, V_9347, V_83567, V_96731, V_107530, V_103254, V_37056, V_22769, V_67985, V_83569, V_96732, V_83572, V_83570, V_115311, V_33167, V_53020, V_53021, V_53022, V_53023, V_22600, V_103255, V_53024, V_53025, V_53026, V_96733, V_115312, V_115313, V_74000, V_96734, V_83573, V_96735, V_83576, V_96736, V_74002, V_83578, V_96740, V_103386, V_7797, V_37057, V_67986, V_96741, V_53027, V_53028, V_53029, V_53030, V_53031, V_53032, V_53033, V_67987, V_67988, V_104556, V_74003, V_6739, V_83583, V_96745, V_96746, V_83584, V_96752, V_4155, V_33168, V_53034, V_37386, V_33169, V_104557, V_67989, V_67990, V_106629, V_31555, V_33170, V_33171, V_33172, V_53035, V_53036, V_103257, V_74005, V_96753, V_96754, V_74006, V_37387, V_96755, V_96756, V_83589, V_96757, V_37388, V_74008, V_74009, V_74010, V_115314, V_37389, V_115315, V_37390, V_115316, V_107982, V_74011, V_9348, V_37058, V_67991, V_103258, V_83596, V_96763, V_96764, V_107531, V_83598, V_96765, V_67992, V_0244, V_53037, V_96766, V_74012, V_8307, V_83603, V_96770, V_3252, V_1853, V_53038, V_38758, V_103259, V_104558, V_106315, V_115317, V_67993, V_106631, V_96773, V_4156, V_0245, V_74013, V_83609, V_96777, V_96779, V_96780, V_83610, V_96778, V_53039, V_103260, V_62977, V_59648, V_67995, V_67996, V_37059, V_105898, V_59551, V_104559, V_115318, V_67994, V_105899, V_106632, V_96781, V_6740, V_3267, V_8308, V_74015, V_96782, V_96783, V_74016, V_107983, V_37060,
23	153266863	153647227	153:27:00	153.65	q28	TPX007E04	TPX017B0	5	0.380364	-0.521380507410593	3	C4LN, MPC600, MPC298	IRAK1, MECP2, OPN1LW, TEX28P2, OPN1MW, TEX28P1, OPN1MW2, TEX28, TKTL1, FLNA, EMD, RPL10, DNASE1L1, TAZ		V_31575, V_83863, V_83864, V_97104, V_83868, V_97108, V_83869, V_74085	V_83863, V_83864, V_97104, V_74085, V_83868, V_97108, V_37014, V_37363, V_37364, V_0673, V_83869, V_31575, V_37365, V_0674
23	153782867	155186928	153.78	155:19:00	q28	TPX010B11	TPX014F0	16	1,404,061	-0.363013544519161	5	C11, C13, C17 , C22, MPC298	IKBKG, CXorf52, CTAG1A, CTAG1B, CXorf52B, CTAG2, GAB3, DKC1, MPP1, F8, H2AFB1, F8A1, FUNDC2, MTCP1NB, BRCC3, VBP1, RAB39B, CLIC2, H2AFB2, F8A2, F8A3, H2AFB3, TMLHE, SPRY3, VAMP7	hsa-mir-1184, hsa-mir-1184, hsa-mir-1184	V_68060, V_68061, V_68063, V_68064, V_68066, V_68067, V_68068, V_0828, V_0825, V_83870, V_97109, V_97110, V_83872, V_97111, V_97127, V_97128, V_83879, V_97130, V_97129, V_97132, V_97133, V_83884, V_83883, V_83885, V_97134, V_115349, V_9357, V_52965, V_52966, V_52967, V_23331, V_74086, V_74087, V_74090, V_74092	V_74086, V_74087, V_37366, V_68060, V_83870, V_23331, V_52965, V_97109, V_97110, V_83872, V_68061, V_97111, V_105890, V_52966, V_103296, V_44407, V_74090, V_37367, V_37368, V_9357, V_1870, V_52967, V_37015, V_68063, V_97127, V_37016, V_37017, V_97128, V_37018, V_83879, V_68064, V_37019, V_105891, V_104572, V_115349, V_97130, V_97129, V_0828, V_103297, V_22709, V_68066, V_44408, V_68067, V_97132, V_97133, V_83884, V_83883, V_0825, V_74092, V_83885, V_97134, V_68068

AMPLIFICATIONS (in 2 or more cases)

Chrom	Start	End	Start MB	End MB	Cytobands	Start Probe	End Probe	Probes	Length MB	maxM	max.overlap	Cases	Genes	miRNAs	aCGH CNVs	Full CNVs
1	38415142	41009944	38:42:00	41:01:00	p34.3-p34.2	TP0125A04	TP0120G0	31	2,594,802	114,815,243,545,795	2	C4, MPC960	SF3A3, FHL3, UTP11L, POU3F1, RRAGC, MYCBP, GJA9, RHBDL2, AKIRIN1, NDUFS5, MACF1, BMP8A, PABPC4, HEYL, NT5C1A, HPCAL4, PPIE, BMP8B, OXCT2, TRIT1, MYCL1, MFSD2, CAP1, PPT1, RLF, TMCO2, ZMPSTE24, COL9A2, SMAP2, ZNF643, ZNF642, DEM1, ZNF684		V_2305, V_8322, V_103399, V_38178, V_38179, V_38180, V_31620, V_63490, V_63496, V_63507, V_63526, V_63534, V_63543, V_63552, V_58388, V_58389, V_58390, V_3289, V_4219, V_84177, V_84178, V_74449, V_84179, V_84182, V_84181, V_84183, V_74452, V_74453, V_84184, V_84185, V_115807, V_115818, V_115829, V_115840, V_115851, V_115862, V_53124, V_9881, V_48318, V_48320, V_48321	V_115807, V_63490, V_38178, V_63496, V_84177, V_48318, V_84178, V_74449, V_84179, V_115818, V_115829, V_58388, V_63507, V_115840, V_2305, V_38179, V_31620, V_9881, V_84182, V_84181, V_84183, V_74452, V_23108, V_106723, V_33926, V_63526, V_63534, V_74453, V_84184, V_48320, V_58389, V_63543, V_53124, V_3289, V_5487, V_8322, V_48321, V_4219, V_60817, V_115851, V_38993, V_84185, V_97338, V_38180, V_103399, V_63552, V_58390, V_115862
8	33813206	41855494	33.81	41.86	p12-p11.21	TP0811B04	TP0809A0	93	8,042,288	144,034,104,782,479	5	C9, C10, C18, C21, M	RPL10AP3, UNC5D, KCNU1, ZNF703, ERLIN2, PROSC, GPR124, BRF2, RAB11FIP1, GOT1L1, ADRB3, EIF4EBP1, ASH2L, STAR, LSM1, BAG4, DDHD2, PPAPDC1B, WHSC1L1, LETM2, FGFR1, C8orf86, TACC1, PLEKHA2, HTRA4, TM2D2, ADAM9, ADAM32, ADAM5P, ADAM3A, ADAM18, ADAM2, IDO1, C8orf4, ZMAT4, SFRP1, GOLGA7, GINS4, AGPAT6, NKX6-3, ANK1, MYST3	hsa-mir-486	V_2749, V_9090, V_9091, V_103788, V_103789, V_103790, V_103791, V_38715, V_38716, V_38096, V_38717, V_7683, V_7684, V_7685, V_7686, V_7687, V_31439, V_33030, V_33031, V_33032, V_65105, V_65107, V_65108, V_65109, V_65110, V_65111, V_65112, V_65113, V_65114, V_65116, V_65117, V_65119, V_59470, V_59471, V_59472, V_59473, V_59476, V_4589, V_3728, V_95365, V_82560, V_95366, V_82561, V_82568, V_95373, V_95375, V_82572, V_95376, V_95377, V_82573, V_95378, V_95379, V_82574, V_95380, V_95381, V_82575, V_82576, V_95382, V_82578, V_95385, V_95387, V_95388, V_95389, V_82580, V_95391, V_95392, V_82581, V_95393, V_82582, V_95394, V_82583, V_95395, V_106294, V_112155, V_112156, V_112158, V_112161, V_112162, V_112166, V_112168, V_112169, V_106595, V_107920, V_10250, V_10251, V_52517, V_52518, V_52521, V_52523, V_52528, V_52529, V_52530, V_52531, V_52532, V_52533, V_70332, V_70333	V_9090, V_107456, V_100315, V_100316, V_65105, V_95365, V_65107, V_100317, V_82560, V_95366, V_82561, V_65108, V_70332, V_59470, V_38715, V_103788, V_112155, V_65109, V_82568, V_95373, V_65110, V_9091, V_107458, V_100320, V_1196, V_100321, V_62792, V_43242, V_112156, V_95375, V_82572, V_95376, V_95377, V_36812, V_82573, V_95378, V_22601, V_38716, V_103789, V_65111, V_65112, V_23872, V_6610, V_112158, V_6611, V_1778, V_4589, V_62793, V_70333, V_65113, V_95379, V_59471, V_52517, V_52518, V_59472, V_2749, V_36815, V_105114, V_112161, V_59473, V_36816, V_105798, V_65114, V_106294, V_37462, V_7683, V_82574, V_95380, V_106595, V_31439, V_1779, V_103790, V_106668, V_33030, V_38096, V_1462, V_107920, V_3728, V_1197, V_1463, V_1198, V_112162, V_1460, V_1199, V_1200, V_1461, V_1465, V_95381, V_1464, V_112166, V_82575, V_82576, V_95382, V_7684, V_82578, V_95385, V_33031, V_52521, V_52523, V_100322, V_65116, V_6612, V_100323, V_36817, V_65117, V_33032, V_95387, V_38717, V_103791, V_1466, V_1780, V_10250, V_52528, V_52529, V_52530, V_52531, V_52532, V_52533, V_22754, V_62795, V_95388, V_95389, V_7685, V_7686, V_39524, V_82580, V_95391, V_95392, V_105799, V_105116, V_112168, V_59476, V_23044, V_65119, V_82581, V_95393, V_100324, V_82582, V_95394, V_7687
8	46851417	73266692	46.85	73:27:00	q11.1-q13.3	TP0816G07	TP0809C1	263	26,415,275	120,138,809,984,784	3	C4, C5, C6, C21	KIAA0146, CEBPD, PRKDC, MCM4, UBE2V2, EFCAB1, SNAI2, C8orf22, SNTG1, PXDNL, PCMTD1,ST18, FAM150A, RB1CC1, NPBWR1, OPRK1, ATP6V1H, RGS20, TCEA1, LYPLA1, MRPL15, SOX17, RP1, XKR4, TMEM68, TGS1, LYN, RPS20, MOS, PLAG1, CHCHD7, SDR16C5, SDR16C6, PENK, IMPAD1, C8orf71, FAM110B, UBXN2B, CYP7A1, SDCBP, NSMAF, TOX, CA8, RAB2A, RLBP1L1, ASPH, NKAIN3, GGH, TTPA, YTHDF3, IFITM8P, BHLHE22, CYP7B1, ARMC1, MTFR1, PDE7A, DNAJC5B, TRIM55, CRH, RRS1, ADHFE1, C8orf46, MYBL1, VCPIP1, C8orf44, SGK3, PTTG3, C8orf45, TCF24, LRRC67, COPS5, CSPP1, ARFGEF1, CPA6, PREX2, C8orf34, RPS15AP25, SULF1, SLCO5A1, PRDM14, NCOA2, TRAM1, LACTB2, XKR9, EYA1, MSC, TRPA1	hsa-mir-124-2	V_2750, V_2751, V_2752, V_2753, V_2754, V_2755, V_2756, V_2757, V_2758, V_8601, V_9093, V_8602, V_8603, V_8604, V_8605, V_8606, V_8607, V_103792, V_103793, V_103794, V_103795, V_103796, V_103798, V_103799, V_103800, V_103801, V_37813, V_37765, V_38026, V_38718, V_37943, V_37766, V_38082, V_38719, V_37854, V_106660, V_37922, V_38723, V_38724, V_37998, V_7688, V_7689, V_7691, V_7694, V_7696, V_7697, V_7701, V_7703, V_7704, V_7705, V_31440, V_31441, V_33033, V_33034, V_31442, V_33035, V_33036, V_33037, V_33038, V_33039, V_33040, V_33041, V_31443, V_31444, V_31445, V_31446, V_33042, V_33043, V_33044, V_33045, V_33046, V_65124, V_65125, V_65126, V_65131, V_65132, V_65133, V_65136, V_65138, V_65140, V_65141, V_65142, V_65143, V_65144, V_65147, V_65148, V_65149, V_65150, V_65151, V_65152, V_65154, V_65157, V_65158, V_65159, V_65160, V_65162, V_65163, V_59478, V_59482, V_59484, V_59488, V_59489, V_59490, V_4590, V_3729, V_3730, V_4591, V_3731, V_3732, V_4592, V_4593, V_3733, V_3734, V_3735, V_3736, V_4594, V_4595, V_4596, V_4597, V_4598, V_95400, V_95402, V_95401, V_82589, V_82590, V_82591, V_95403, V_95404, V_82592, V_82593, V_95405, V_82594, V_82595, V_95407, V_82596, V_95406, V_95408, V_82597, V_95409, V_95410, V_95412, V_95413, V_95415, V_95417, V_95418, V_82601, V_95419, V_95424, V_95425, V_95427, V_95430, V_95431, V_95432, V_82607, V_82608, V_95434, V_95435, V_82609, V_95437, V_95439, V_95438, V_95442, V_95443, V_95444, V_95445, V_82613, V_95446, V_95447, V_95448, V_82614, V_82615, V_82616, V_95449, V_95450,	V_70338, V_105120, V_95400, V_52548, V_70339, V_62803, V_70340, V_95402, V_95401, V_70341, V_52549, V_52550, V_4590, V_37614, V_52551, V_52552, V_52553, V_29793, V_52554, V_52555, V_52556, V_53645, V_82589, V_22743, V_53187, V_65124, V_62804, V_82590, V_82591, V_95403, V_95404, V_70342, V_52558, V_82592, V_82593, V_95405, V_70343, V_82594, V_82595, V_95407, V_82596, V_95406, V_95408, V_65125, V_5255, V_100332, V_62806, V_70344, V_106596, V_43426, V_23875, V_2750, V_9601, V_65126, V_107460, V_103792, V_31440, V_7688, V_37813, V_9600, V_52559, V_107921, V_36818, V_70347, V_70346, V_70354, V_70355, V_70353, V_107922, V_70351, V_107923, V_70356, V_70357, V_70352, V_107925, V_70358, V_70349, V_70350, V_107926, V_70348, V_70359, V_37299, V_3729, V_110166, V_29451, V_37300, V_29452, V_7689, V_3730, V_31441, V_82597, V_95409, V_95410, V_6617, V_112173, V_59478, V_4591, V_70360, V_95412, V_95413, V_100335, V_59482, V_6618, V_70361, V_2751, V_8601, V_3731, V_112174, V_37765, V_112175, V_100336, V_44348, V_95415, V_107461, V_23876, V_100337, V_95417, V_9093, V_2752, V_10252, V_3732, V_0633, V_36819, V_105802, V_62807, V_37463, V_100338, V_112177, V_43643, V_95418, V_39526, V_44349, V_65131, V_33033, V_38026, V_1201, V_1781, V_82601, V_33034, V_65132,
8	73704795	146236289	73:07:00	146:24:00	q13.3-q24.3	TP0809E01	TP0816E0	711	72,531,494	175,338,821,929,454	7	C4, C4LN, C5, C8, C	KCNB2, TERF1, C8orf84, RPL7, RDH10, STAU2, UBE2W, TCEB1, TMEM70, LY96, JPH1, GDAP1, PI15, CRISPLD1, HNF4G, ZFHX4, PXMP3, PKIA, FAM164A, IL7, STMN2, HEY1, MRPS28, TPD52, ZBTB10, ZNF704, PAG1, FABP5, PMP2, FABP9, FABP4, FABP12, IMPA1, SLC10A5, ZFAND1, CHMP4C, SNX16, HNRNPA1P4, RALYL, LRRCC1, E2F5, CA13, CA1, CA3, CA2, REXO1L1, PSKH2, ATP6V0D2, SLC7A13, WWP1, FAM82B, CPNE3, CNGB3, CNBD1, WDR21C, MMP16, RIPK2, OSGIN2, NBN, DECR1, CALB1, TMEM64, TMEM55A, OTUD6B, LRRC69, SLC26A7, RUNX1T1, C8orf83, FAM92A1, RBM12B, C8orf39, TMEM67, PPM2C, CDH17, GEM, RAD54B, KIAA1429, DPY19L4, INTS8, CCNE2, C8orf38, TP53INP1, PLEKHF2, C8orf37, GDF6, UQCRB, MTERFD1, PTDSS1, SDC2, TSPYL5, MTDH, LAPTM4B, MATN2, RPL30, C8orf47, HRSP12, POP1, NIPAL2, KCNS2, STK3,VPS13B, COX6C, RGS22, FBXO43, POLR2K, SPAG1, RNF19A, ANKRD46, SNX31, PABPC1, RPS26P6, YWHAZ, ZNF706, NACAP1, GRHL2, NCALD, RRM2B, UBR5, ODF1, KLF10, AZIN1, ATP6V1C1, C8orf56, BAALC, FZD6, CTHRC1, SLC25A32, WDSOF1, RIMS2, TM7SF4, DPYS, LRP12, ZFPM2, OXR1, ABRA, ANGPT1, RSPO2, EIF3E, TTC35, TMEM74, TRHR, NUDCD1, ENY2, PKHD1L1, EBAG9, KCNV1, CSMD3, TRPS1, EIF3H, UTP23, RAD21, C8orf85, SLC30A8, MED30, EXT1, SAMD12, TNFRSF11B, COLEC10, MAL2, NOV,	hsa-mir-599, hsa-mir-875, hsa-mir-1273, hsa-mir-548a-3, hsa-mir-2053, hsa-mir-548d-1, hsa-mir-1204, hsa-mir-1205, hsa-mir-1207, hsa-mir-1208, hsa-mir-30b, hsa-mir-30d, hsa-mir-151, hsa-mir-937, hsa-mir-661, hsa-mir-939, hsa-mir-1234	V_34662, V_2759, V_2760, V_2761, V_2762, V_2763, V_2764, V_2765, V_2766, V_2767, V_2768, V_2769, V_2770, V_2771, V_2772, V_2773, V_2774, V_2775, V_2776, V_2777, V_2778, V_2779, V_9094, V_8608, V_8609, V_8610, V_9095, V_9096, V_9097, V_8611, V_8612, V_9098, V_8613, V_8614, V_9099, V_8615, V_9100, V_103802, V_103803, V_103804, V_103805, V_103806, V_103807, V_103809, V_103810, V_103812, V_103813, V_103814, V_103815, V_103816, V_103817, V_103820, V_103821, V_103822, V_103823, V_103824, V_103825, V_103826, V_103827, V_103828, V_103829, V_37984, V_38726, V_37942, V_37768, V_38727, V_38729, V_38696, V_37914, V_38697, V_38698, V_38699, V_38011, V_38700, V_38030, V_37762, V_37763, V_38701, V_37923, V_38702, V_37902, V_38703, V_38705, V_37764, V_38706, V_38117, V_7707, V_7708, V_7712, V_7713, V_7659, V_7660, V_7662, V_7663, V_7665, V_7668, V_7669, V_7670, V_7671, V_7672, V_7673, V_31447, V_33047, V_33048, V_31449, V_33049, V_33050, V_33051, V_33052, V_33053, V_33054, V_33055, V_31450, V_31451, V_33056, V_31452, V_33057, V_33058, V_33059, V_31453, V_31454, V_31455, V_31456, V_33060, V_31457, V_31458, V_31459, V_65166, V_65167, V_65168, V_65169, V_65170, V_65171, V_65172, V_65174, V_65175, V_65176, V_65178, V_65179, V_65182, V_65183, V_65184, V_65185, V_65186, V_65187, V_65189, V_65191, V_65192, V_65193, V_65194, V_65195, V_65196, V_65197, V_65200, V_65201, V_65202, V_65203, V_65204, V_65205, V_65207, V_65209, V_65210, V_65211, V_65213, V_65215, V_65216, V_65217, V_65218, V_65219, V_65220, V_65224, V_65225, V_65226, V_65227, V_65229, V_65230, V_65232, V_65233, V_65234, V_65235, V_65236, V_65237, V_65239, V_65240, V_65241, V_65242, V_65243, V_65244, V_65245, V_65247, V_65248, V_65249, V_65251, V_65252, V_65253, V_65254, V_65256, V_65257, V_65258, V_65259, V_65261, V_65262, V_65263, V_65264, V_65266, V_65267, V_65268, V_65270, V_65271, V_65272, V_65273, V_65274, V_65275, V_65276, V_65277, V_65279, V_65280, V_65281, V_65283, V_65284, V_65285, V_65286, V_65288, V_65289, V_65291, V_65292, V_65293, V_65294, V_65296, V_59491, V_59493, V_59494, V_59495, V_59497, V_59498, V_59499, V_59500, V_59440, V_59441, V_59442, V_59443, V_59444, V_59445, V_59449, V_59450, V_59451, V_59452, V_59453, V_59454, V_59455, V_59456, V_59457, V_4599, V_3737, V_0119, V_4600, V_4601, V_3738, V_4602,	V_36863, V_100387, V_22682, V_105814, V_43609, V_39528, V_6635, V_106094, V_23878, V_44358, V_107475, V_100389, V_52618, V_52619, V_6636, V_65166, V_95497, V_95498, V_100390, V_6637, V_65167, V_44359, V_100391, V_110169, V_95500, V_52620, V_52621, V_65168, V_36865, V_36866, V_95502, V_65169, V_37468, V_95503, V_103802, V_82645, V_37984, V_36867, V_36868, V_100392, V_59491, V_100393, V_82646, V_95504, V_22544, V_62821, V_105815, V_105124, V_112203, V_65170, V_82647, V_95505, V_44360, V_95507, V_106297, V_112204, V_100394, V_70392, V_70390, V_100395, V_52622, V_100396, V_4599, V_52623, V_100397, V_95508, V_52624, V_7707, V_43272, V_106095, V_23879, V_39529, V_6638, V_100400, V_6639, V_95509, V_70393, V_95510, V_6640, V_95511, V_65171, V_95512, V_100401, V_62823, V_9094, V_65172, V_95513, V_95517, V_38726, V_65174, V_9605, V_65175, V_95521, V_3737, V_95522, V_7708, V_2759, V_70394, V_82656, V_95524, V_0119, V_65176, V_95525, V_105817, V_6641, V_47902, V_6642, V_112205, V_65178, V_105125, V_59493, V_95532, V_1793, V_100403, V_6643, V_100404, V_22775, V_95533, V_65179, V_95535, V_95534, V_82662, V_70397, V_95536, V_95537, V_82663, V_100405, V_100406, V_70398, V_105126, V_82665, V_95538, V_52630, V_52631, V_100407, V_52632, V_52633, V_53474, V_112206, V_95539, V_107476, V_36874, V_29795, V_29794, V_29796, V_29797, V_100409, V_53427, V_8608, V_59494, V_100410, V_95540, V_36875, V_82666, V_95541, V_105127, V_112208, V_22795, V_59495, V_65182, V_37469, V_1467, V_1794, V_103803, V_31447, V_37942, V_52634, V_36876, V_65183, V_95542, V_112210, V_4600, V_65184, V_100411, V_6644, V_4601, V_3738, V_4602, V_7712, V_52637, V_112211, V_37768, V_82667, V_95543, V_70400, V_36877, V_105129, V_0636, V_36878, V_0637, V_36879, V_36880, V_107933, V_36881, V_4603, V_107934, V_70401, V_105130, V_0638, V_36882, V_4604, V_0639, V_36883, V_0640, V_36884, V_4605,
17	36563876	39502562	36:56:00	39:05:00	q12-q21.2	TP1707B02	TP1709D0	34	2,938,686	229,054,079,831,212	3	C18, C24, MPC600	C17orf96, MLLT6, CISD3, PCGF2, PSMB3, PIP4K2B, CCDC49, C17orf98, RPL23, LASP1, FBXO47, PLXDC1, CACNB1, RPL19, STAC2, FBXL20, MED1, CDK12, NEUROD2, PPP1R1B, STARD3, TCAP, PNMT, PERLD1, ERBB2, C17orf37, GRB7, IKZF3, ZPBP2, GSDMB, ORMDL3, GSDMA, PSMD3, CSF3, MED24, THRA, NR1D1, MSL1, CASC3, RAPGEFL1, WIPF2, CDC6, RARA, GJD3, TOP2A, IGFBP4, TNS4, CCR7, SMARCE1, KRT222P, KRT24, KRT25, KRT26, KRT27, KRT28, KRT10, TMEM99, KRT12, KRT20, KRT23, KRT39, KRT40, KRTAP3-3, KRTAP3-2, KRTAP3-1, KRTAP1-5, KRTAP1-4, KRTAP1-3, KRTAP1-1, KRTAP2-2, KRTAP2-1, KRTAP2-4, KRTAP4-9, KRTAP4-8, KRTAP4-11, KRTAP4-12, KRTAP4-6, KRTAP4-5, KRTAP4-4, KRTAP4-3, KRTAP4-2, KRTAP9-2, KRTAP9-3, KRTAP9-8, KRTAP9-6, KRTAP9-7, KRTAP16-1, KRTAP17-1, KRT33A		V_9286, V_104123, V_38355, V_38356, V_38006, V_106650, V_38806, V_7158, V_7159, V_30828, V_32177, V_32178, V_30829, V_67187, V_67188, V_67190, V_67192, V_67193, V_67194, V_67195, V_67196, V_67197, V_58791, V_4034, V_2224, V_5006, V_4035, V_4036, V_0205, V_88452, V_77589, V_88453, V_77590, V_77591, V_88454, V_77592, V_77593, V_88455, V_88456, V_77594, V_88457, V_77595, V_88458, V_88459, V_77596, V_88460, V_77597, V_77598, V_77599, V_77601, V_77602, V_77603, V_77604, V_88461, V_88462, V_88464, V_88465, V_88466, V_77605, V_88463, V_77600, V_77606, V_88467, V_107576, V_106196, V_114397, V_114399, V_114401, V_114402, V_106426, V_106427, V_49852, V_49853, V_49854, V_49855, V_49856, V_49857, V_72920, V_72924, V_72925, V_72927, V_72926, V_72928, V_72929	V_4034, V_2224, V_88452, V_77589, V_72920, V_67187, V_30828, V_88453, V_114397, V_67188, V_109457, V_114399, V_67190, V_5006, V_49852, V_102451, V_114401, V_77590, V_77591, V_49853, V_88454, V_49854, V_49855, V_77592, V_39200, V_107576, V_102452, V_49856, V_4035, V_67192, V_39201, V_49857, V_72924, V_43948, V_72925, V_102455, V_67193, V_38355, V_77593, V_88455, V_88456, V_77594, V_88457, V_77595, V_9286, V_72927, V_72926, V_109490, V_67194, V_88458, V_88459, V_106426, V_38356, V_77596, V_88460, V_77597, V_32177, V_4036, V_35452, V_22862, V_72928, V_72929, V_67195, V_77598, V_77599, V_77601, V_77602, V_77603, V_77604, V_88461, V_88462, V_88464, V_88465, V_88466, V_109501, V_32178, V_106196, V_106427, V_67196, V_7158, V_77605, V_88463, V_102457, V_38006, V_106650, V_35453, V_109512, V_77600, V_35454, V_0499, V_35455, V_58791, V_77606, V_88467, V_109523, V_23198, V_67197, V_105983, V_114402, V_7159, V_38806, V_104123, V_102458, V_30829, V_109535, V_0205
20	45213999	55159095	45:21:00	55:16:00	q13.12-q13.31	TP2005G05	TP2005H0	115	9,945,096	137,084,421,921,317	3	C4, C4LN, MPC600	SLC13A3, TP53RK, SLC2A10, EYA2, ZMYND8, NCOA3, SULF2, PREX1, ARFGEF2, CSE1L, STAU1, DDX27, ZNFX1, KCNB1, PTGIS, B4GALT5, SLC9A8, SPATA2, RNF114, SNAI1, TMEM189, CEBPB, PTPN1, FAM65C, PARD6B, BCAS4, ADNP, DPM1, MOCS3, KCNG1, NFATC2, ATP9A, SALL4, ZFP64, TSHZ2, ZNF217, BCAS1, CYP24A1, PFDN4, DOK5, CBLN4, MC3R, C20orf108, AURKA, CSTF1, CASS4, C20orf43, GCNT7, C20orf106, C20orf107	hsa-mir-645, hsa-mir-1302-5	V_3219, V_3220, V_3221, V_3222, V_8887, V_8888, V_104192, V_104193, V_38444, V_38445, V_7308, V_7309, V_7310, V_7311, V_32425, V_32426, V_31041, V_67703, V_67704, V_67705, V_67707, V_67708, V_67709, V_67710, V_67711, V_67712, V_67714, V_67715, V_4103, V_2251, V_5139, V_5140, V_5141, V_0226, V_5142, V_5143, V_90590, V_90591, V_79224, V_79225, V_79226, V_79227, V_90596, V_90594, V_79228, V_79229, V_90592, V_90597, V_79231, V_79232, V_90598, V_79233, V_90599, V_79230, V_79234, V_90601, V_79236, V_79237, V_79238, V_79235, V_90603, V_79240, V_90606, V_79242, V_90608, V_90612, V_90613, V_90610, V_79241, V_90614, V_90609, V_79243, V_90611, V_90615, V_90616, V_90617, V_79244, V_90607, V_90618, V_90622, V_114925, V_114926, V_114928, V_114929, V_114932, V_114934, V_114935, V_114936, V_114938, V_106479, V_53893, V_9825, V_10583, V_50671, V_50678, V_50679, V_50680, V_50681, V_50682, V_50684, V_50685, V_50686, V_50687, V_50688, V_50689, V_50690, V_50696, V_50697, V_50698, V_73521, V_73522, V_73524, V_73523, V_73525, V_73526, V_73527, V_73530, V_73533, V_73532, V_73534, V_73535, V_73537, V_73538, V_73539	V_8887, V_114925, V_3219, V_7308, V_107131, V_50671, V_3220, V_7309, V_38444, V_104192, V_114926, V_110411, V_102979, V_90590, V_90591, V_79224, V_6114, V_6115, V_4103, V_32425, V_79225, V_79226, V_0538, V_35927, V_2251, V_23627, V_59771, V_37232, V_35928, V_0539, V_35929, V_73521, V_104459, V_23628, V_5139, V_73522, V_1996, V_79227, V_90596, V_90594, V_67703, V_79228, V_79229, V_90592, V_90597, V_32426, V_79231, V_79232, V_90598, V_107132, V_73524, V_35930, V_0540, V_35931, V_79233, V_90599, V_79230, V_73523, V_59645, V_110412, V_102981, V_102983, V_22989, V_79234, V_90601, V_79236, V_79237, V_79238, V_79235, V_5140, V_47899, V_102984, V_114928, V_39294, V_90603, V_5141, V_110413, V_6116, V_7310, V_6117, V_7311, V_8888, V_110415, V_102985, V_3221, V_114929, V_31041, V_6118, V_6119, V_50678, V_67704, V_50679, V_50680, V_9825, V_50681, V_107133, V_110417, V_102987, V_44050, V_6120, V_67705, V_106479, V_73525, V_73526, V_73527, V_114932, V_0226, V_102988, V_102989, V_5142, V_102990, V_5143, V_47945, V_50682, V_67707, V_6121, V_50684, V_1524, V_35932, V_67708, V_79240, V_90606, V_79242, V_90608, V_90612, V_90613, V_90610, V_79241, V_90614, V_90609, V_79243, V_90611, V_90615, V_90616, V_90617, V_79244, V_90607, V_90618, V_73530, V_104460, V_114934,
20	55929883	57147285	55.93	57:15:00	q13.31-q13.32	TP2006B01	TP2001F0	9	1,217,402	145,558,956,277,928	2	C4, C4LN	RAE1, RBM38, CTCFL, PCK1, ZBP1, PMEPA1, C20orf85, ANKRD60, PPP4R1L, RAB22A, VAPB, APCDD1L		V_7312, V_31042, V_67718, V_4104, V_90623, V_114943, V_114944, V_73543	V_7312, V_90623, V_114943, V_73543, V_4104, V_31042, V_67718, V_6126, V_114944
20	58579298	59535518	58:58:00	59:54:00	q13.33	TP2001E10	TP2001C0	10	0.95622	104,220,600,393,435	2	C4, C4LN	CDH26, C20orf197	hsa-mir-646	V_38446, V_38447, V_67721, V_67722, V_67724, V_67725, V_59032, V_90634, V_79256, V_53131, V_10586, V_10587, V_50700, V_50701, V_73546, V_73547	V_59032, V_103006, V_73546, V_67721, V_103008, V_73547, V_10586, V_50700, V_53131, V_67722, V_38446, V_90634, V_67724, V_38447, V_10587, V_50701, V_79256, V_67725, V_103009

aCGH: microarray-comparative genomic hybridization; Chr: chromosome; CNV: copy number variation; MB: megabase.

Supplementary Table S6. Summary of mRNA-sequencing statistics

Sample ID	Total sequencing per lane (GB)	Aligned reads per lane (GB)
MPC08	1.43	1.77
MPC10	1.92	2.67
MPC70	1.33	1.89
MPC71	1.37	1.88
MPC72	1.35	1.83

Supplementary Table S7. Recurrent alterations identified through re-analysis of public datasets (n=185)

Index case			Recurrence					
**Fusion partner gene**	**Index case**	**Index case in frame**	**Sample characteristics**	**Sample phenotype**	**In frame**	**DNA or RNA**	**Reference**	**Number of samples sequenced in study**
BCAS4	MPC10	Y	Cell line MCF7	ER+ HER2-	Y	RNA	Robinson et al; Edgren et al	89; 5
C17orf57	MPC71	N	Primary breast cancer	ER-HER2+	N	DNA	Nik-Zainal et al	21
CDK12	MPC71	N	Cell line HCC1954	ER- HER2+	Unknown	DNA	Stephens et al	24
CDK12	MPC71	N	Cell line HCC2218	ER- HER2+	Unknown	DNA	Stephens et al	24
CDK12	MPC71	N	Primary breast cancer	ER- HER2+	N	DNA	Nik-Zainal et al	21
CDK12	MPC71	N	Cell line MDA-MB-361	ER+ HER2+	Y	RNA	Robinson et al	89
CDK12	MPC71	N	Primary breast cancer	HER2+	Y	DNA	Banerji et al	22
FAF1	MPC10	N	Primary breast cancer	ER- HER2-	N	DNA	Nik-Zainal et al	21
LASP1	MPC71	N	Primary breast cancer	ER+ HER2-	N	DNA	Nik-Zainal et al	21
LASP1	MPC71	N	Primary breast cancer	Unknown	Unknown	DNA	Stephens et al	24
NSF	MPC71	N	Primary breast cancer	ER+ HER2+	Unknown	DNA	Nik-Zainal et al	21
RAE1	MPC10	N	Cell line BT474	ER+ HER2+	N	RNA	Robinson et al	89
RAE1	MPC10	N	Primary breast cancer	ER+ HER2-	N	RNA	Robinson et al	89
USH2A	MPC10	N	Primary breast cancer	ER+ HER2-	N	DNA	Nik-Zainal et al	21
USH2A	MPC10	N	Primary breast cancer	Unknown	N	DNA	Ellis et al	46
